# Systematic Structure–Activity Relationship Study of Nalfurafine Analogues toward Development of Potentially Nonaddictive Pain Management Treatments

**DOI:** 10.1021/acs.jmedchem.4c00646

**Published:** 2024-05-30

**Authors:** Celsey M. St. Onge, Piyusha P. Pagare, Yi Zheng, Michelle Arriaga, David L. Stevens, Rolando E. Mendez, Justin L. Poklis, Matthew S. Halquist, Dana E. Selley, William L. Dewey, Matthew L. Banks, Yan Zhang

**Affiliations:** † Department of Medicinal Chemistry, Virginia Commonwealth University, 800 E. Leigh Street, Richmond, Virginia 23219, United States; ‡ Department of Pharmacology and Toxicology, Virginia Commonwealth University, 410 North 12th Street, Richmond, Virginia 23298, United States; § Department of Pharmaceutics, Virginia Commonwealth University, 410 North 12th Street, Richmond, Virginia 23298, United States; ∥ Institute for Drug and Alcohol Studies, 203 East Cary Street, Richmond, Virginia 23298, United States

## Abstract

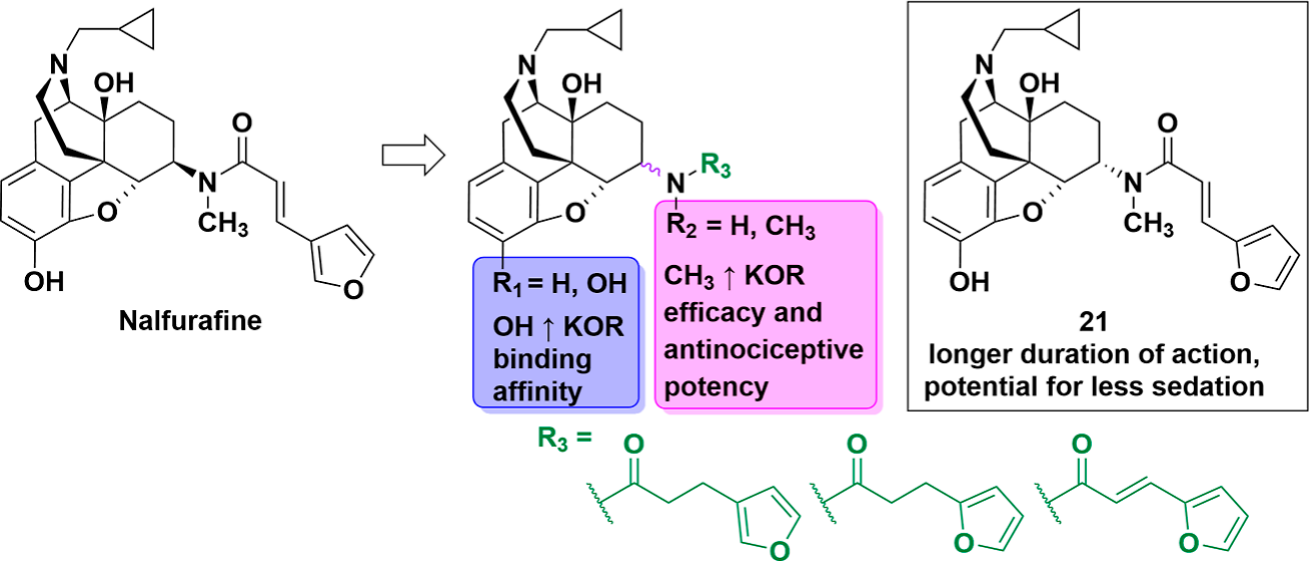

Despite the availability of numerous pain medications, the current array of Food and Drug Administration-approved options falls short in adequately addressing pain states for numerous patients and consequently worsens the opioid crisis. Thus, it is imperative for basic research to develop novel and nonaddictive pain medications. Toward addressing this clinical goal, nalfurafine (NLF) was chosen as a lead and its structure–activity relationship (SAR) systematically studied through design, syntheses, and *in vivo* characterization of 24 analogues. Two analogues, **21** and **23**, showed longer durations of action than NLF in a warm-water tail immersion assay, produced *in vivo* effects primarily mediated by KOR and DOR, penetrated the blood–brain barrier, and did not function as reinforcers. Additionally, **21** produced fewer sedative effects than NLF. Taken together, these results aid the understanding of NLF SAR and provide insights for future endeavors in developing novel nonaddictive therapeutics to treat pain.

## Introduction

As of 2022, there were 87 Food and Drug Administration (FDA)-approved compounds for the treatment of pain.^1^ However, despite this medication abundance, many patients still experience inadequate pain relief with currently available treatments.^2−5^ Opioids are typically used for more severe pain; however, they are intended for short-term use because their long-term use may lead to substance use disorders.^6^ Further, many pain medications interact with other commonly coprescribed medications resulting in either potentially dangerous drug–drug interactions or decreasing pain management efficacy. In addition, certain patients may not respond well to current pain treatments. Thus, despite the availability of the currently FDA-approved treatments for pain, there are still significant limitations in their efficacy, safety, and suitability for numerous patient populations. Developing a nonaddictive pain medication can help address these gaps and provide more pain management options for patients and healthcare providers to address the multitude of painful conditions.

Opioid ligands have been exploited for numerous years in pursuit of therapeutics to ameliorate drug misuse and pain. Beyond the “one drug one target” approach, this search has included development of bivalent and dual-acting ligands. These approaches fall into the category of polypharmacology wherein a single drug is designed to interact with multiple targets with the goal of simultaneously increasing therapeutic efficacy while decreasing the undesirable effect profile.^7^ This concept was first proposed by Bryan Roth in 2004; he acknowledged that “magic bullets” or drugs selective for single targets have been mostly unsuccessful for treating highly complex central nervous system (CNS) disorders and that “magic shotguns” or selectively nonselective drugs for multiple targets may be more beneficial.^8^

Specifically, a dual-acting ligand is one molecule that is intentionally selective for two receptors. Several dual-acting ligands have been developed for the opioid receptor subtypes. First, dual-acting mu opioid receptor (MOR)/kappa opioid receptor (KOR) ligands such as buprenorphine, pentazocine, and nalbuphine have been approved for pain management.^9−15^ Second, MOR/delta opioid receptor (DOR) dual-acting ligands have been proposed to produce synergistic analgesic effects improving the analgesia while decreasing the likelihood of development of dependence or tolerance and studied preclinically.^16,17^ Third, MOR/nociceptin orphanin FQ opioid peptide receptor (NOP) dual-acting ligands are thought to enhance analgesic effects and reduce the likelihood of developing tolerance and dependence while having lower abuse potential.^18^ Of note, cebranopadol, a pan agonist for the four opioid receptors, is in clinical trials for the treatment of cancer pain and substance use disorders.^19,20^ Additionally, MOR agonists/dopamine D3 (D3R) antagonists/partial agonists have been identified as analgesics with reduced opioid misuse liability and might have potential as therapeutics for neuropathic pain and inflammation.^21,22^

Alternatively, dual-acting ligands that do not target the MOR can be developed to decrease the potential for abuse liability of analgesics. KOR and DOR agonists have both been demonstrated to have antinociceptive properties, meaning they can alleviate or prevent the sensation of pain.^23,24^ The similar mechanisms of action by which KOR and DOR modify painful stimuli imply that they may produce either complementary or synergistic antinociception.^24−26^ KOR/DOR dual-agonist small molecules with antinociceptive capacity have been synthesized as proof-of-concept by multiple research groups.^27−29^ Although there are no FDA-approved selective KOR/DOR dual-acting ligands currently, this comparatively understudied chemical profile provides an opportunity for further exploration. Hence, we hypothesized that dual-functioning KOR/DOR agonists might serve as nonaddictive candidate pain management medications and that simultaneous KOR and DOR activation may provide enhanced antinociception allowing for antinociceptive doses below the threshold for adverse effects at either receptor alone.

Nalfurafine (NLF), also known as TRK-820, was first synthesized by the Nagase lab in 1998 and was clinically approved in Japan as a second-line antipruritic to treat uremic pruritis in end-stage renal disease patients on hemodialysis in 2009 (Remitch 2.5 μg/day).^30−35^ Nalfurafine has additionally been studied for uremic pruritis in clinical trials which have thus far not yielded its approval in the United States.^36−43^ Prior to the discovery of its antipruritic indication, NLF was originally developed as an analgesic for postoperative pain and it exhibited antinociceptive activity in a variety of pain models.^30,44−49^ While other KOR agonists have been limited by adverse side effects such as psychotomimesis, dysphoria, and sedation, NLF does not result in dysphoria or psychotomimesis at potential therapeutic doses. In general, it has been found that antinociceptive effects are produced at doses smaller than those that produce hypolocomotion, conditioned place aversion, and motor incoordination.^44,47,49−55^ One suggested explanation for NLF’s lack of dysphoric properties is its potential as a biased KOR agonist with greater bias in humans than rodents,^49,56−58^ but see also with further explorations.^47,59,60^ This may be a dose/efficacy difference that has not been systematically studied as it has been with biased MOR agonists. Additionally, interpretational limits are presented by the methods used to calculate bias, *e.g.*, use of MAPK activation as an end point or use of different cell systems to measure GTPγS and β-arrestin2 activation.^57,60−63^ Though NLF might not be suitable for repurposing due to its narrow therapeutic window, it may serve as a lead compound for pain management due to its intriguing pharmacological profile and mild side effects.

**Figure 1:**
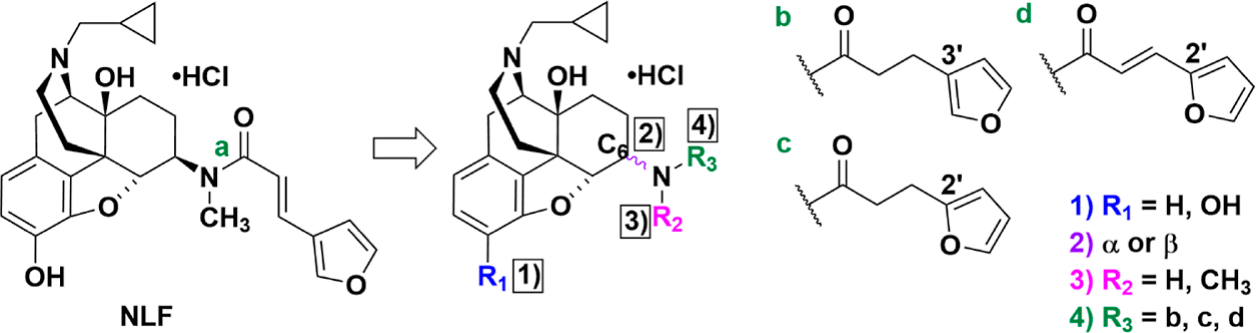
Newly designed ligands to study SAR of NLF. Four points of modifications are color-coded.

Several NLF structure–activity relationship (SAR) explorations have been performed and reported.^59,64−73^ However, systematic and comprehensive SAR reports on NLF are still missing from literature resources leading to numerous knowledge gaps.^59,64−76^ To this end, we recently reported 7 NLF analogues that maintained the same carboxamide side chain at the C_6_-position of the epoxymorphinan skeleton as NLF itself.^28^ Herein, we further vary the C_6_-position carboxamide side chain to explore the SAR of this lead with a more robust data set (Figure 1). Thus, the SAR of NLF was systematically studied *via* the design and synthesis of a series of 24 analogues. Subsequently, these analogues were subjected to cellular and behavioral pharmacological testing in a tier wise manner.

## Results and Discussion

### Molecular Design

Rational molecular design was based around variation of four key components of the NLF molecular structure (Figure 1). (1) Compounds either maintain or lack the 3-hydroxy group present on NLF. (2) Compounds vary in their configurational arrangement, taking either the 6α or 6β isomeric form. The 3-hydroxy functional group may be crucial for recognition of binding sites of three opioid receptors, and combined with the C_6_ conformation may govern selectivity, as demonstrated by us and others.^34,77^ (3) The amide nitrogen atom is secondary or tertiary *via* the presence of a methyl group. This may verify whether the *N*-methyl is important for opioid agonist activity. Additionally, it may influence the hydrophobicity of the ligands as well. (4) The carboxamide side chain (R_3_) at the C_6_ position in NLF is an α,β-unsaturated carbonyl group, a known Michael acceptor. Due to their inherent reactivity, Michael acceptors are often associated with risks and classified as pan-assay interference compounds (PAINS). However, despite this general concern, NLF has demonstrated safety and minimal side effects in patients. Taking into consideration the presence of a highly reactive group, the carboxamide side chain is varied in saturation and attachment points on the furan ring. Hence, the linker between the furan ring and amide carbon atoms is either conjugated *via* a *trans* double bond or saturated (*i.e.*, single bond). This also enables assessment of the degree of rigidity that is tolerated and/or flexibility that is needed for binding. It has been proposed that the structural distance of the furan ring (address) from the epoxymorphinan skeleton (message) is essential for the message and this distance should be fixed at a 6-carbon length, thus the linker length was not modified.^69^ The furan ring attachment point is varied between the 2′ and 3′ position; this variation probes the binding pocket for the optimal binding interaction. Collectively, these variations may result in changes in affinity, efficacy, and biophysical properties, thus expanding the SAR profile. Of note, the 17-cyclopropylmethyl and 14-hydroxy group were not varied because their modification has been previously found to decrease opioid binding affinity.^64^ Taken together, these variations resulted in 24 NLF analogues; four of the 24 compounds were previously reported by us, and one was reported by others, while all five of these were studied in a different perspective herein.^69,78^

#### Physiochemical Property Prediction

The Advanced Chemistry Development Inc. (ACD) Percepta software was applied to predict the physiochemical properties (*e.g.*, cLogP, cpKa, cLogD, and TPSA) of designed ligands to assess their drug-likeness.^79^ According to Table S1, the designed ligands meet all criteria for Lipinski’s rule of five and fall within the range of optimal LogD values (1.8–3.1) for drug molecules.^80,81^ Further, base p*K*_a_ values are below 10.5, ideal for CNS permeability and supportive of potential for oral delivery.^82^ Finally, all TPSA values are under 96, suggesting good absorption.^83^ Overall, none of the compounds required flagging for exclusion, thus all compounds were synthesized for further testing.

### Chemical Synthesis

**Scheme 1:**
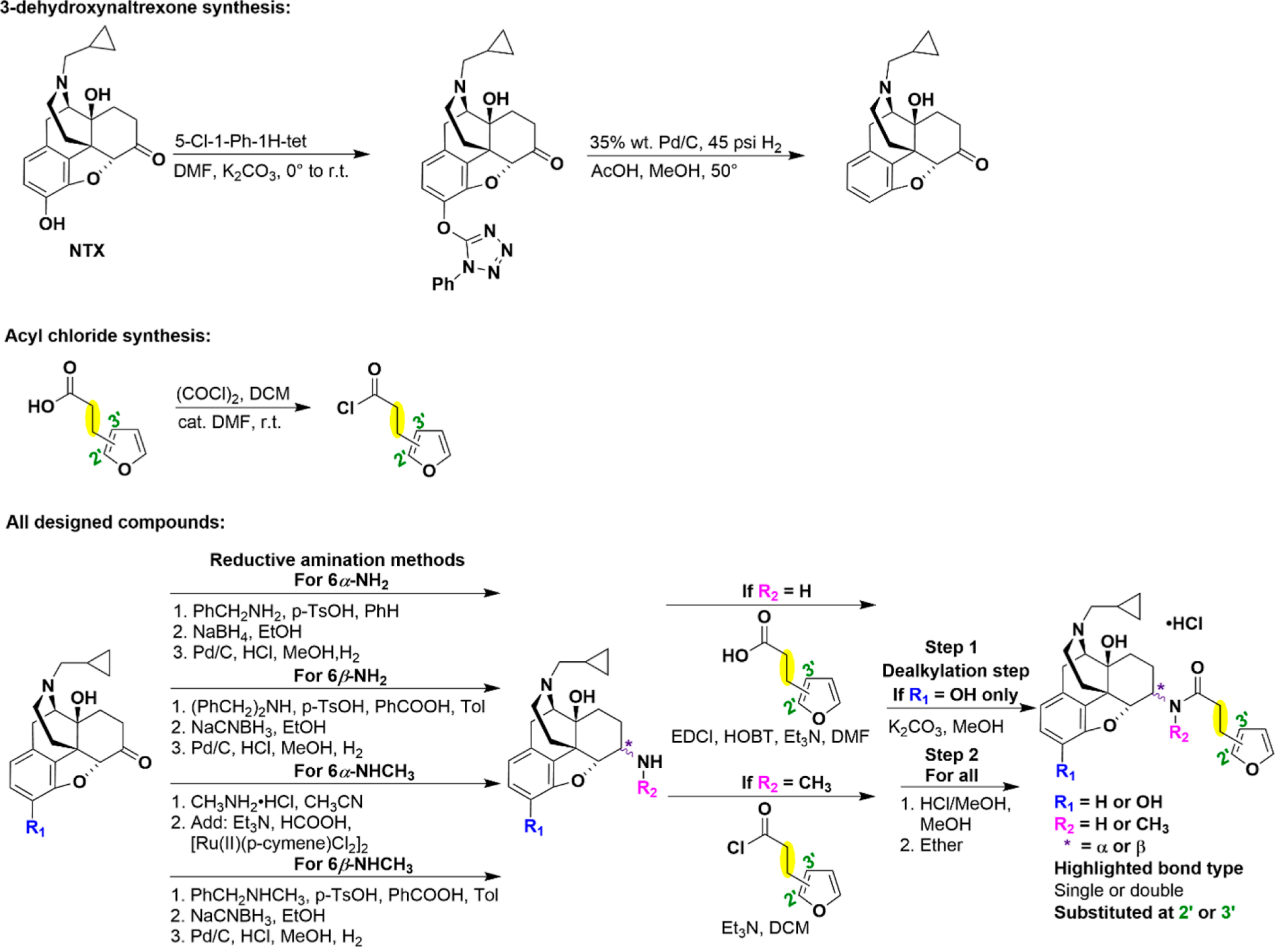
Overall Synthetic Route to Produce Designed Ligands

All 24 designed ligands were synthesized according to previously reported procedures as depicted in Scheme 1. Briefly, 3-dehydroxynaltrexone was first synthesized from naltrexone.^84^ Naltrexone and 3-dehydroxynaltrexone were then subjected to four different stereoselective reductive amination methods followed by catalytic hydrogenation under acidic conditions (where applicable) to yield eight different naltrexamines: namely, 6α-naltrexamine, 6α-3-dehydroxynaltrexamine, 6β-naltrexamine, 6β-3-dehydroxynaltrexamine, 6α-*N*-methyl-naltrexamine, 6α-*N*-methyl-3-dehydroxynaltrexamine, 6β-*N*-methyl-naltrexamine, and 6β-*N*-methyl-3-dehydroxynaltrexamine.^85−97^ Then primary amines were coupled to carboxylic acids utilizing the EDCI/HOBt coupling reaction while secondary amines were coupled to acyl chlorides under basic conditions. Acyl chlorides were prepared fresh from their carboxylic acid counterparts prior to coupling if they were commercially unavailable.^98^ For derivatives containing α,β-unsaturated side chains, the stereochemistry of the commercially purchased carboxylic acids or acyl chlorides was confirmed to be *E* (*trans*). Hydrolysis under basic conditions was performed on the 12 3- hydroxy derivatives due to the possibility of esterification at the hydroxy group. All final compounds were then transformed into their respective hydrochloride salt forms and characterized by ^1^H NMR, ^13^C NMR, HRMS, HPLC, and MP. Additionally, the retention of *E*-stereochemistry of the final compounds was also confirmed by the coupling constant in their respective ^1^H NMR.

### *In Vitro* Pharmacological Studies

#### Radioligand Binding Assays

Competitive radioligand binding assays characterize both the binding affinity and the selectivity of synthesized ligands. These assays were carried out according to previously published methods on Chinese hamster ovary (CHO) cell membrane homogenate expressing the canonical opioid receptors, namely the MOR, KOR, and DOR.^86−88,90,99−101^ Briefly, [^3^H]naloxone was used to label the MOR and [^3^H]diprenorphine labeled the KOR and DOR. As demonstrated in Table 1, the synthesized ligands had a range of binding affinities for the opioid receptors. Half of the compounds (**1**, **5**, **6**, **7**, **8**, **13**, **15**, **16**, **17**, **21**, **23**, and **24**) had subnanomolar binding for the KOR. Additionally, half of the compounds had subnanomolar binding for the MOR (**5**, **6**, **7**, **8**, **13**, **14**, **15**, **16**, **21**, **22**, **23**, and **24**), while three compounds (**5**, **13**, and **21**) had subnanomolar binding for the DOR.

**Table 1:**
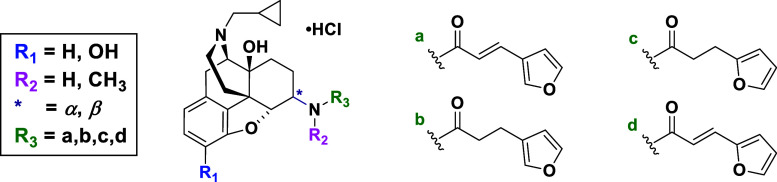
Binding Affinity and Selectivity for the KOR, MOR, and DOR as Determined by Radioligand Binding Assays

Compd.	Compd. variations				radioligand binding affinity (*K*_*i*_, nM)			selectivity ratio	
	R_1_	R_2_	α/β	R_3_	KOR	MOR	DOR	μ/κ	δ/κ
NLF^a^	OH	CH_3_	β	a	0.19 ± 0.01	0.72 ± 0.09	115.9 ± 20.9	4	610
NMF^a^	OH	CH_3_	α	a	0.12 ± 0.02	0.47 ± 0.06	0.64 ± 0.11	4	5
1	H	CH_3_	α	b	0.48 ± 0.07	7.74 ± 0.69	48.58 ± 6.51	16	101
2	H	H	α	b	161.4 ± 11.8	54.52 ± 2.01	153.9 ± 26.5	0	1
3	H	CH_3_	β	b	34.48 ± 6.27	43.81 ± 2.85	890.8 ± 52.5	1	26
4	H	H	β	b	8.37 ± 1.44	17.26 ± 1.68	749.4 ± 59.1	2	90
5	OH	CH_3_	α	b	0.13 ± 0.02	0.18 ± 0.02	0.59 ± 0.06	1	5
6^b^	OH	H	α	b	0.78 ± 0.04	0.44 ± 0.10	3.54 ± 0.50	1	5
7	OH	CH_3_	β	b	0.28 ± 0.05	0.53 ± 0.06	8.42 ± 0.90	2	30
8^b^	OH	H	β	b	0.47 ± 0.03	0.21 ± 0.01	32.70 ± 3.47	0	70
9	H	CH_3_	α	c	1.00 ± 0.18	14.14 ± 1.40	46.82 ± 8.37	14	47
10	H	H	α	c	73.77 ± 12.19	53.08 ± 6.06	90.21 ± 13.02	1	1
11	H	CH_3_	β	c	50.40 ± 6.84	93.60 ± 10.44	532.3 ± 92.5	2	11
12	H	H	β	c	23.58 ± 3.79	27.49 ± 2.90	799.9 ± 96.7	1	34
13	OH	CH_3_	α	c	0.12 ± 0.02	0.15 ± 0.01	0.37 ± 0.07	1	3
14^b^	OH	H	α	c	1.52 ± 0.27	0.72 ± 0.10	2.94 ± 0.26	0	2
15	OH	CH_3_	β	c	0.32 ± 0.03	0.66 ± 0.05	7.32 ± 1.08	2	23
16^b^	OH	H	β	c	0.61 ± 0.07	0.28 ± 0.02	33.00 ± 3.58	0	54
17	H	CH_3_	α	d	0.62 ± 0.04	16.82 ± 1.34	68.41 ± 11.20	27	110
18	H	H	α	d	248.5 ± 32.0	107.15 ± 8.37	456.1 ± 26.4	0	2
19	H	CH_3_	β	d	4.11 ± 0.57	214.5 ± 26.5	2263.6 ± 338.1	52	551
20	H	H	β	d	7.03 ± 0.92	42.44 ± 2.34	550.9 ± 64.0	6	78
21	OH	CH_3_	α	d	0.14 ± 0.02	0.20 ± 0.02	0.93 ± 0.21	1	7
22	OH	H	α	d	1.08 ± 0.03	0.53 ± 0.06	8.75 ± 0.58	0	8
23	OH	CH_3_	β	d	0.22 ± 0.03	0.78 ± 0.05	7.93 ± 1.02	4	36
24	OH	H	β	d	0.17 ± 0.02	0.30 ± 0.02	12.84 ± 2.37	2	76

a Binding affinity data previously published in ref (28).

b Compound first published in ref (81).

Based on the radioligand binding assay, multiple SAR trends were observed. First, KOR binding affinity seemed to be enhanced with the presence of the 3-hydroxyl group (Table 1). At the onset of the study, the importance of the 3-hydroxyl group for KOR binding affinity was still debatable.^28,59,64,102^ Our previous studies suggested that the 3-hydroxyl group may not be critical for KOR binding affinity as two out of four 3-dehydroxyl compounds synthesized showed subnanomolar KOR binding affinity.^28^ However, with the additional strength of this data set, a trend in the increase in KOR binding affinity with the presence of a 3-hydroxyl group is less ambiguous. Table 1 shows head-to-head comparisons between the binding affinity of compounds with and without the 3-hydroxyl group for three different side chain variations (*e.g.*, **1***vs***5**). Further, it is interesting that the binding affinity difference is smaller between 3-hydroxy and 3-dehydroxy α-methylnaltrexamine compounds than other naltrexamine variations (*i.e.*, 3-dehydroxy α-methylnaltrexamine compounds have unusually high binding affinity). Second, all compounds with a 3-hydroxyl group had subnanomolar binding affinity for the MOR with others ranging from single to triple digit nanomolar (Table 1). This aligns with published SAR regarding the presence of the 3-OH and MOR binding affinity.^103,104^ Third, in all pairwise comparisons, DOR binding affinity seemed higher in the presence of the 3-hydroxyl and lower when in the β configuration (Table 1). Additionally, there seemed to be no apparent affect of the methyl group, the α/β configuration, or the R_3_ side chain on KOR or MOR binding affinity.

#### [^35^2S]-GTPγS Functional Assays

As a functional assay used to assess *in vitro* potency and efficacy, the [^35^S]-GTPγS-binding assay was applied to assess all synthesized ligands at the canonical opioid receptors, MOR, KOR, and DOR. The efficacy of ligands in this assay was related to that of the respective full agonist control U50,488, DAMGO, and SNC80 for the KOR, MOR, and DOR, respectively, following previously established protocols.^85−88,90,93,96,99−101,105^ As displayed in Table 2, most synthesized ligands functioned as full KOR agonists with partial MOR agonism and partial to full DOR agonism. More specifically, all compounds had KOR efficacy above 80% except compound **2**, and all compounds had MOR efficacy lower than 40% except compounds **8** and **16**. Compounds that had DOR efficacy higher than 80% include **2**, **10**, **17**, **19**, **20**, **21**, and **23**.

**Table 2:**
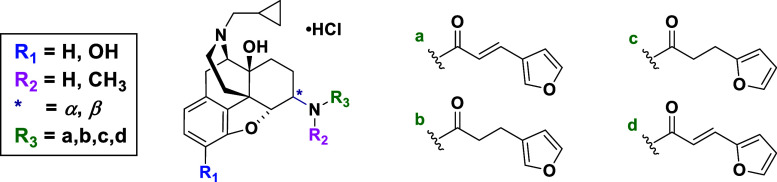
Functional Activity at the KOR, MOR, and DOR as Determined by [^35^S]-GTPγS Assays^c^

Compd.	Compd. variations				[^35^S]-GTPγS functional activity					
					KOR		MOR		DOR	
	R_1_	R_2_	α/β	R_3_	EC_50_ (nM)	*E*_max_ (%)	EC_50_ (nM)	*E*_max_ (%)	EC_50_ (nM)	*E*_max_ (%)
NLF^a^	OH	CH_3_	Β	a	0.26 ± 0.03	95.95 ± 4.13	0.52 ± 0.12	30.24 ± 3.73	141.4 ± 14.8	65.68 ± 6.42
NMF^a^	OH	CH_3_	Α	a	0.08 ± 0.00	92.54 ± 3.02	0.60 ± 0.03	34.34 ± 0.53	2.68 ± 0.74	113.00 ± 10.60
1	H	CH_3_	α	b	6.50 ± 1.11	97.51 ± 4.80	20.03 ± 3.49	11.53 ± 0.81	137.3 ± 26.5	56.44 ± 2.60
2	H	H	α	b	1014 ± 176	66.06 ± 5.20	199.7 ± 63.1	8.80 ± 0.55	429.4 ± 62.6	93.13 ± 1.57
3	H	CH_3_	β	b	245.6 ± 29.2	86.42 ± 0.63	223.6 ± 44.9	13.77 ± 1.86	5783 ± 829	66.86 ± 3.52
4	H	H	β	b	117.1 ± 21.2	81.39 ± 7.72	185.2 ± 38.0	14.19 ± 1.27	1687 ± 116	55.78 ± 2.39
5	OH	CH_3_	α	b	0.04 ± 0.01	105.9 ± 2.2	0.37 ± 0.05	17.95 ± 1.81	0.75 ± 0.09	68.31 ± 1.00
6^b^	OH	H	α	b	6.11 ± 0.83	80.32 ± 4.73	2.31 ± 0.87	27.62 ± 1.96	4.26 ± 0.76	76.87 ± 5.40
7	OH	CH_3_	β	b	0.99 ± 0.04	101.8 ± 1.05	4.30 ± 0.47	12.20 ± 0.92	19.17 ± 1.62	51.44 ± 2.78
8^b^	OH	H	β	b	0.90 ± 0.10	95.91 ± 1.43	0.63 ± 0.12	42.61 ± 2.61	14.29 ± 1.27	46.59 ± 4.06
9	H	CH_3_	α	c	18.42 ± 1.05	91.38 ± 1.46	76.50 ± 48.47	11.22 ± 1.55	211.8 ± 33.8	70.51 ± 5.90
10	H	H	α	c	441.8 ± 29.9	86.49 ± 1.55	278.1 ± 106.8	10.21 ± 1.33	348.3 ± 40.2	85.65 ± 3.74
11	H	CH_3_	β	c	145.9 ± 27.1	109.3 ± 5.9	5625 ± 2130	22.95 ± 2.39	1105 ± 108	61.71 ± 4.27
12	H	H	β	c	367.5 ± 31.8	86.83 ± 0.69	104.4 ± 24.2	10.84 ± 1.13	2395 ± 200	70.98 ± 6.10
13	OH	CH_3_	α	c	0.04 ± 0.01	100.6 ± 2.5	0.31 ± 0.04	15.26 ± 1.08	0.66 ± 0.06	63.07 ± 2.60
14^b^	OH	H	α	c	5.22 ± 0.79	80.75 ± 3.02	2.77 ± 0.73	21.77 ± 2.76	3.55 ± 0.77	75.25 ± 2.46
15	OH	CH_3_	β	c	1.48 ± 0.17	100.7 ± 1.0	3.58 ± 0.59	15.26 ± 0.50	16.87 ± 2.57	38.64 ± 1.37
16^b^	OH	H	β	c	1.62 ± 0.12	93.71 ± 1.01	1.33 ± 0.15	44.53 ± 2.72	16.77 ± 2.96	40.91 ± 2.33
17	H	CH_3_	α	d	5.97 ± 0.42	110.3 ± 7.9	124.7 ± 17.1	22.85 ± 2.01	439.7 ± 32.2	101.7 ± 1.7
18	H	H	α	d	1372 ± 89	85.2 ± 6.52	1509 ± 948	15.98 ± 2.91	1242 ± 147	75.00 ± 5.10
19	H	CH_3_	β	d	53.93 ± 3.33	96.87 ± 8.02	887.9 ± 363.3	11.00 ± 0.68	5985 ± 581	82.09 ± 2.63
20	H	H	β	d	177.6 ± 18.1	99.67 ± 5.88	1145 ± 984	12.81 ± 3.00	860.3 ± 148.1	81.03 ± 3.71
21	OH	CH_3_	α	d	0.04 ± 0.00	106.9 ± 2.6	0.58 ± 0.17	19.70 ± 0.70	1.28 ± 0.28	83.25 ± 2.13
22	OH	H	α	d	13.68 ± 2.52	89.56 ± 7.25	2.97 ± 0.45	12.65 ± 1.05	21.11 ± 1.41	60.95 ± 2.21
23	OH	CH_3_	β	d	0.14 ± 0.02	106.5 ± 0.7	5.31 ± 0.53	14.22 ± 1.77	16.37 ± 3.25	96.13 ± 6.52
24	OH	H	β	d	0.54 ± 0.11	98.53 ± 0.60	6.76 ± 1.99	7.33 ± 0.65	13.07 ± 2.09	42.81 ± 4.01

a Functional activity data previously published in ref (28).

b MOR functional activity for compound first published in ref (79).

c Efficacy values (*E*_max_) are represented as percent of U50,488, DAMGO, and SNC80 full agonist controls for KOR, MOR, and DOR, respectively.

Multiple SAR trends were observed based on the functional activity data. First, the methyl group seemed to be important for KOR agonism. In almost all cases, the KOR efficacy was higher when the methyl group was present (Table 2). The two exceptions were compounds **10** and **20**. This helped clarify some literature inconsistencies surrounding this trend wherein the role of the methyl group was less apparent.^28,69^ Second, compounds bearing a 3-OH, desmethyl, β configuration, and saturated R_3_ side chains had MOR efficacy values above 40%, while all other synthesized ligands had MOR efficacy values below 40% (Table 2; compounds **8** and **16**). Third, compounds bearing a 3-OH and saturated R_3_ side chain (*i.e.*, compounds **5**–**8** and **13**–**16**) as well as their unsaturated counterparts that additionally lack the methyl group (*i.e.*, compounds **22** and **24**) had partial DOR agonism and maintained potency. These findings, particularly those regarding both MOR and DOR partial agonism, are important to note as it has been proposed as a novel drug discovery method that diverge from traditional target-based strategies. These approaches involve engaging multiple targets simultaneously, partially activating target receptors or activating the target receptors in innovative ways. Insights gleaned from functional studies such as above could be instrumental in customizing the efficacy of the MOR, KOR, and DOR for future molecular design. These findings offer valuable guidance in the quest to develop opioids devoid of adverse effects, a pursuit that has spanned more than a century.

### *In Vivo* Warm-Water Tail Immersion Assay

Warm-water tail immersion (WWTI) assays are often employed to rapidly characterize opioid receptor ligands; this is a technique that we have used for many years.^28,80,85,86,88−90,94,96,99−101^ Briefly, to assess potential antinociception, a mouse’s tail is dipped into a warm-water bath. The mouse then flicks its tail to remove the thermal stimulus. The duration that the tail remains in the warm-water bath is recorded with longer durations yielding higher percentage of the maximum possible effect (MPE %) values—indicative of antinociception.

**Figure 2:**
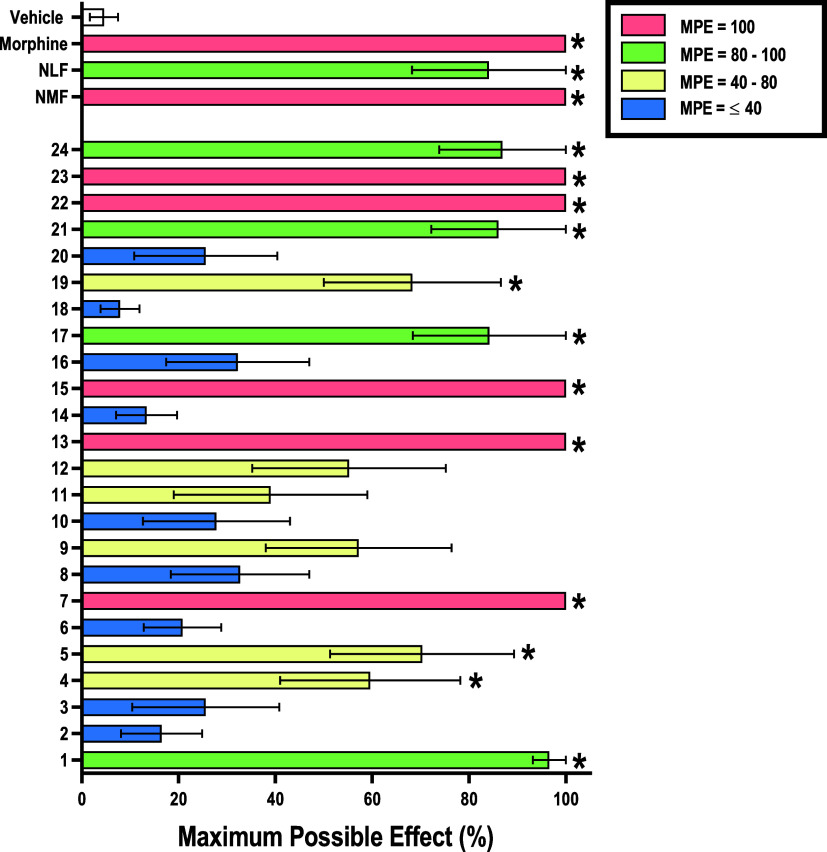
Preliminary *in vivo* agonism screen of synthesized ligands in the warm–water tail immersion assay at a single dose of 10 mg/kg in male Swiss Webster mice (*n* = 6/group). Ordinate: control/comparatorcompounds (morphine, NLF) and synthesized ligands 1–24.Abscissa: percent MPE. All points represent the mean ± SEM. The compounds were divided into four main groups based on their MPE under these conditions MPE = 100% (salmon), MPE = 80–100% (lime green), MPE = 40–80% (pale yellow), and MPE = ≤ 40% (blue). *Denotes significant difference relative to the vehicle condition (open bar), defined as *p* < 0.05. NLF and NMF data previously published in ref (28). Compounds **6**, **8**, **14**, and **16** were first published in ref (81).

First, the antinociceptive potential of all 24 compounds was examined at a single dose of 10 mg/kg in the WWTI assay. Here, withdrawal latency was measured 20 min after subcutaneous administration of the compounds. In this assay, morphine (10 mg/kg) served as a positive control and produced a 100% MPE indicating that the mouse’s tail remained in the warm-water bath for the maximal time allowed of 10 s. Figure 2 shows that 12 of the 24 compounds produced significant antinociception when compared to the vehicle group, attributable to their high affinity and efficacy at the KOR and range of DOR affinity and efficacy. Further, 9 of these 12 compounds resulted in ≥80% MPEs including **1**, **7**, **13**, **15**, **17**, **21**, **22**, **23**, and **24** which agreed with the most promising compounds identified by the *in vitro* binding and functional assays. The lack of significant antinociceptive effect among the remaining 12 compounds at this dose may be attributable to various factors such as comparatively lower *in vitro* KOR and DOR affinities (compounds **2**, **3**, **10**, **11**, **12**, **18**, and **20**) as well as lower *in vitro* potencies or pharmacokinetic limitations (compounds **6**, **8**, **9**, **14**, and **16**). Overall, half of the compounds demonstrated significant antinociception. Therefore, only compounds that produced ≥80% MPE at this dose were selected for further study.

The analogues with the highest antinociceptive potential (≥80% MPE) were then studied to determine their *in vivo* potencies. Here, varying compound doses were subcutaneously administered 20 min prior to tail withdrawal latency measurement to obtain ED_50_ values. Table 3 shows that 6 of the 9 compounds were more potent than morphine, and six of them had ED_50_ values lower than 1 mg/kg.^108^ Compounds **21** and **23** were approximately equipotent to more potent than both NLF and NMF (Table 3).^28^

**Table 3:**
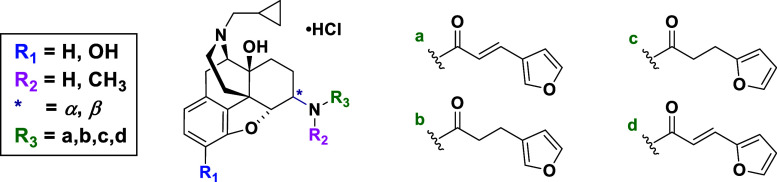
Potency Determination of Selected Ligands Using a Warm–Water Tail Immersion Assay in Male Swiss Webster Mice (*n* = 6/Group)

Compd.	Compd. variations				potency
	R_1_	R_2_	*	R_3_	ED_50_ [95% CI] mg/kg
morphine^a^					3.9 [3.3–4.6]
NLF^b^	OH	CH_3_	β	a	0.046 [0.016–0.126]
NMF^b^	OH	CH_3_	α	a	0.037 [0.014–0.096]
1	H	CH_3_	α	b	0.553 [0.353–0.866]
7	OH	CH_3_	β	b	0.443 [0.293–0.669]
13	OH	CH_3_	α	c	0.123 [0.039–0.384]
15	OH	CH_3_	β	c	0.251 [0.170–0.371]
17	H	CH_3_	α	d	7.796 [0.394–154.348]
21	OH	CH_3_	α	d	0.051 [0.035–0.075]
22	OH	H	α	d	4.077 [1.041–15.968]
23	OH	CH_3_	β	d	0.010 [0.007–0.015]
24	OH	H	β	d	3.322 [0.545–20.251]

a Data adopted from ref (108).

b Data previously published in ref (28).

Interestingly, seven out of the nine compounds that were selected for dose-dependence study bear the R_2_ methyl group suggesting that the presence of this methyl group may also be important for *in vivo* antinociceptive potency. The remaining two aid in forming this conclusion *via* comparison to their more potent methyl-bearing counterparts (**22***vs***21**; **24***vs***23**). This agrees with the results of our previously published study on 8 structurally similar compounds as well wherein NLF and NMF also contain this methyl group.^28^

**Figure 3:**
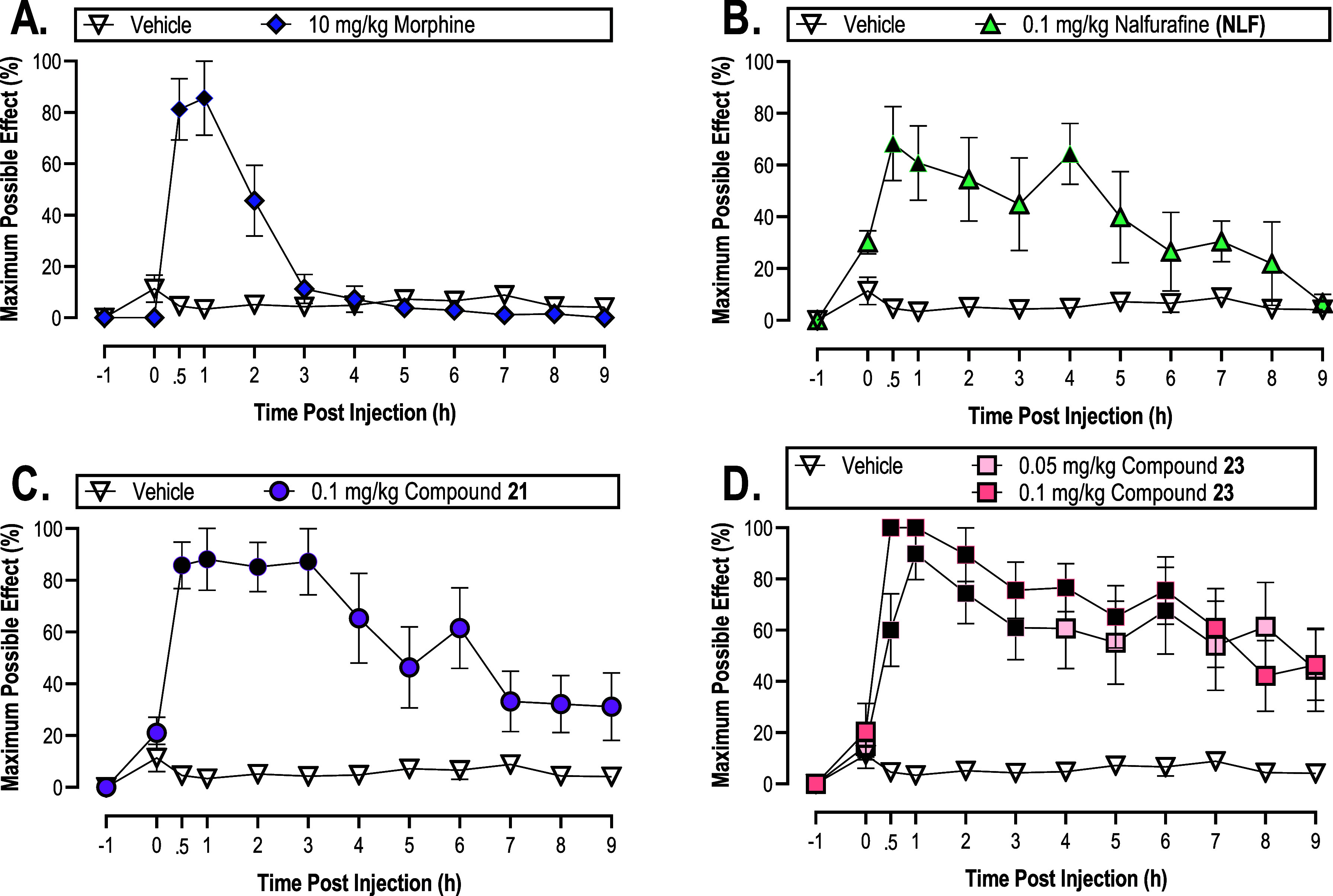
Time course study of (A) morphine, (B) NLF, (C) compound **21**, and (D) compound **23** using the warm–water tail immersion assay in male Swiss Webster mice (*n* = 6/group). Ordinate: percent MPE. Abscissa: time post subcutaneous injection of synthesized ligand in hours. All points represent the mean ± SEM. Black filled symbols denote significant difference relative to the vehicle condition (open symbols), defined as *p* < 0.05.

The two most potent compounds in the WWTI assay (**21** and **23**) were further studied to determine the antinociceptive time course. Here, the test compound was injected subcutaneously, and withdrawal latencies were recorded at subsequent time points to determine the duration of compound effects following published protocol.^28,29^ Doses were chosen to facilitate comparison to our previously reported, structurally related compound NMF, and a dose of 0.05 mg/kg was added to account for the increased potency of compound **23**.^28^ Significant antinociception was produced by morphine 30 min to 1 h postinjection with effects near-baseline by 3 h and back to baseline by 5 h (Figure 3A), similar to previously published studies.^29^ Shown in Figure 3B, 0.1 mg/kg NLF resulted in significant antinociceptive effects 30 min after injection that lasted until the 1 h time point and were significant once more 4 h postinjection with a return to the baseline 9 h postinjection. This is consistent with the findings of previous studies that monitored the time course of NLF using different animal models.^107−109^

Compounds **21** and **23** both showed antinociceptive effects by 30 min postinjection (Figure 3C,D). The antinociceptive effects of 0.1 mg/kg compound **21** remained significant until 3 h postinjection while those of 0.1 mg/kg compound **23** remained significant until 6 h postinjection. Thus, when administered at the same dose, compound **23** had a longer duration of antinociceptive effects. However, compound **23** was more potent than compound **21** in the WWTI studies. To account for this increased potency, a more reasonable comparison may be to compare compound doses at the same potency level. Compounds **21** and **23** have ED_75_ values of 0.1 and 0.05 mg/kg, respectively. The antinociceptive effects of 0.05 mg/kg compound **23** were significant until 3 h postinjection with a return of significance 6 h post injection. Thus, at their ED_75_ dosages, compounds **21** and **23** had equivalent durations of action while compound **21** showed a seemingly more sustained maximal effect. Moreover, these two compounds appeared to have similar onsets at approximately 30 min. One liability of our previously disclosed compound NMF is a short duration of action. With an antinociceptive potency similar to compound **21**, NMF showed antinociceptive effects for 1 h at a dose of 0.1 mg/kg.^28^ Comparatively, both 0.1 mg/kg compound **21** and 0.5 mg/kg compound **23** (with increased potency as previously discussed) had a duration of action of 3 h. Thus, both compounds **21** and **23** have longer durations of antinociceptive action than 10 mg/kg morphine, 0.1 mg/kg NLF, and 0.1 mg/kg NMF in the WWTI assay.^106^

To determine which opioid receptors mediated these potentially antinociceptive effects, a receptor selectivity study was done. Herein, mice were treated with selective opioid receptor antagonists prior to testing with compound **21** or **23** following previously published protocol.^28,29^ Specifically, the MOR antagonist β-funaltrexamine (β-FNA), the KOR antagonist nor-binaltorphimine (nor-BNI), and the DOR antagonist naltrindole (NTI) were subcutaneously injected prior to the subcutaneous injection of compound followed by measurement of withdrawal latency 20 min later.^110−112^

**Figure 4:**
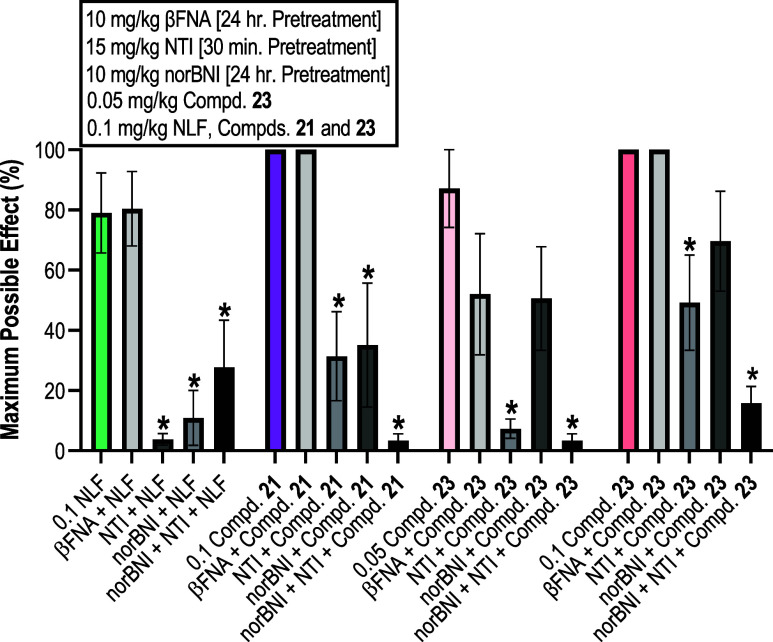
Receptor selectivity study of compounds **21**, **23**, and NLF using the warm–water tail immersion assay in male Swiss Webster mice (*n* = 6/group). Ordinate: percent MPE. Abscissa: combination of synthesized ligands and opioid antagonists administered. All points represent the mean ± SEM *Denotes significant difference relative to the referenced test compound when given alone, defined as *p* < 0.05.

Figure 4 shows that when NTI and nor-BNI were administered separately prior to NLF administration, the resulting MPEs were significantly lower than those when NLF was administered alone indicating the involvement of the DOR and the KOR, respectively. Interestingly the combination of NTI and nor-BNI pretreatment did not result in a more drastic decrease than either antagonist alone. However, the high variance in this data point made it difficult to draw any conclusions. While NLF appeared to be a low efficacy MOR partial agonist in the *in vitro* functional studies (Table 2), Figure 4 demonstrates that it was not antagonized by β-FNA *in vivo*. This discrepancy may be due to multiple factors such as the efficacy threshold of the WWTI assay or the simultaneous involvement of the KOR and DOR. The selectivity of NLF at the opioid receptors has been previously reported and was repeated herein as an internal control to ensure alignment with previously reported data.^44,45^ In previous reports, 10 mg/kg nor-BNI (s.c.) pretreatment antagonized the sedative and antinociceptive effects of 0.1 mg/kg but not 0.03 mg/kg NLF and 20 mg/kg nor-BNI (s.c.) pretreatment antagonized antinociceptive effects of 0.001–0.01 mg/kg NLF.^44,45^ In the current study, nor-BNI when administered at the 10 mg/kg dose (s.c.) was able to antagonize a larger dose of NLF (0.1 mg/kg). The difference in species used may be one potential explanation for the difference between these results and those of previous studies.^45^ Previous studies also reported that 3 mg/kg NTI was unable to antagonize the antinociceptive effects of NLF.^44^ The ability of NTI to antagonize the antinociceptive effects of NLF in the current study and not in the previous one is most likely due to the 5-fold difference in the NTI dose administered.^44^

As for compound **21**, the antinociceptive effects were antagonized by both nor-BNI and NTI while pretreatment with both nor-BNI and NTI together seemed to compound the decrease in compound **21** activity. This indicated that the *in vivo* effects of compound **21** were primarily KOR and DOR mediated. These results mirror the receptor selectivity of our previously published compound NMF.^28^ In contrast, compound **23** (0.05 and 0.1 mg/kg) was antagonized by NTI but showed only a slight, nonsignificant decrease in antinociception with nor-BNI pretreatment. Pretreatment with both NTI and nor-BNI together seemed to result in enhanced antagonism of compound **23** antinociceptive effects. While both compounds **21** and **23** showed low efficacy MOR partial agonism *in vitro* (Table 2), similar to the results of NLF, they were not antagonized by β-FNA *in vivo*.

**Figure 5:**
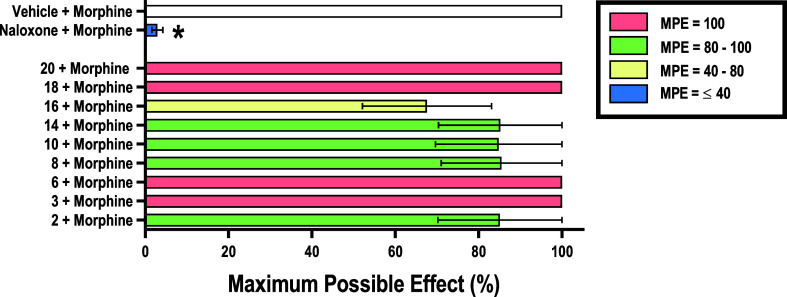
Antagonism screen of synthesized ligands in the warm–water tail immersion assay at a single dose of 10 mg/kg in male Swiss Webster mice (*n* = 6/group). Ordinate: vehicle negative control, naloxone positive control, and selected synthesized ligands as pretreatments to morphine. Abscissa: percent MPE. All points represent the mean ± SEM. The compounds were divided into four main groups based on their MPE under these conditions MPE = 100% (salmon), MPE = 80–100% (lime green), MPE = 40–80% (pale yellow), and MPE = ≤ 40% (blue). *Denotes significant difference relative to the vehicle + morphine condition (open bar), defined as *p* < 0.05. Compounds **6**, **8**, **14**, and **16** first published in ref (79).

In studying the SAR of NLF, it is important to recognize any small structural differences that could result in an agonist to antagonist change. Thus, ligands that did not show antinociceptive potential with MPEs ≤ 40% at the single dose of 10 mg/kg were further studied to monitor their potential to block morphine’s effects using published procedures.^80,85,86^ Here, the compounds were subcutaneously injected 5 min prior to morphine injection followed by measurement of withdrawal latency 20 min later. As the positive control, naloxone sufficiently antagonized morphine antinociception. However, none of the test compounds administered blocked morphine antinociception at the dose tested (Figure 5). There are many potential explanations for this; however, it may be that these compounds have very low MOR potencies which would agree with their *in vitro* results, and larger doses may be necessary to block morphine effects in this assay or that they have unfavorable pharmacokinetics.

### Further *In Vivo* Characterization

#### Locomotor Activity

**Figure 6:**
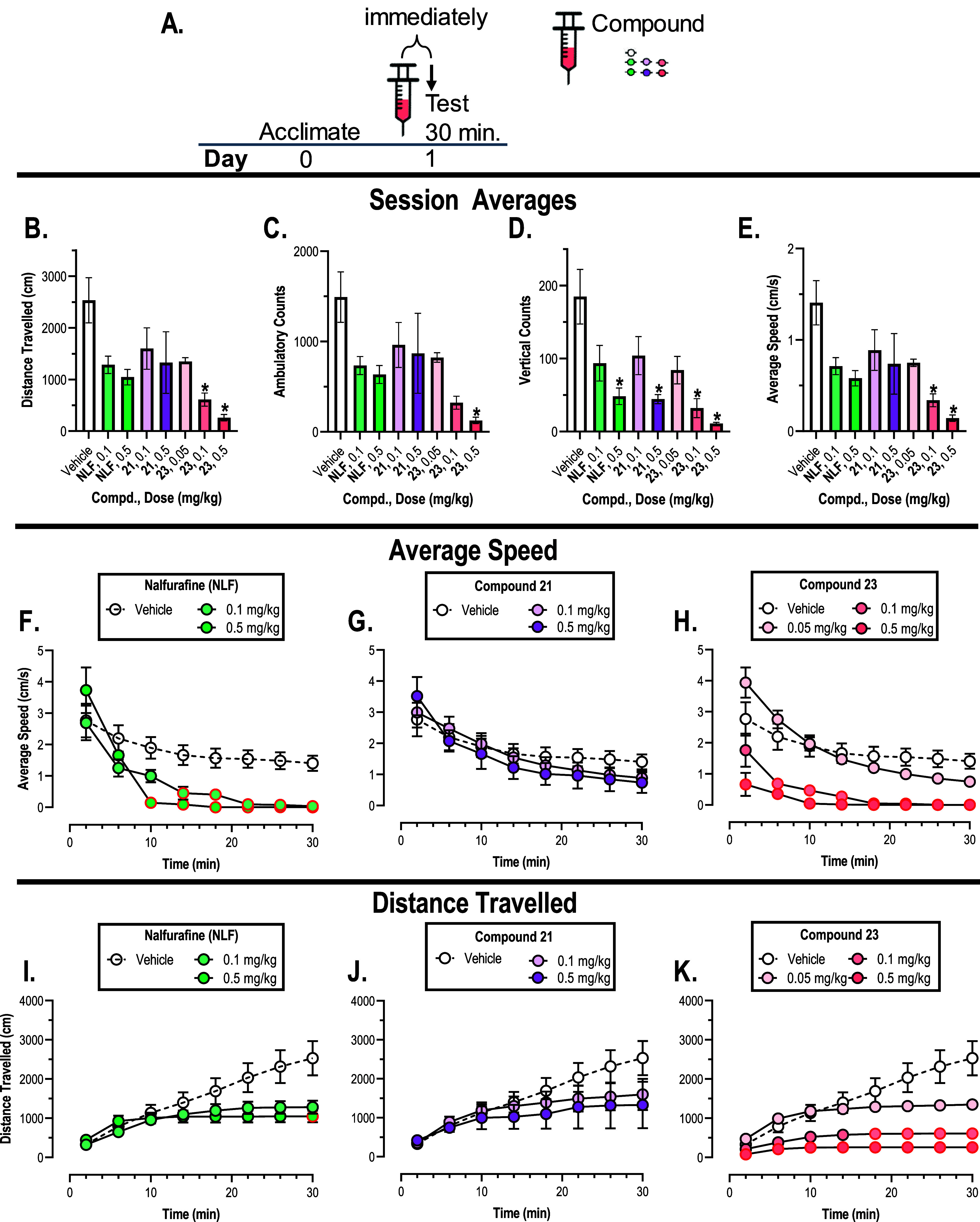
Locomotor activity in male Swiss Webster mice (*n* = 12/group vehicle and compound **21**; *n* = 6/group NLF and compound **23** postinjection of compounds). (A) Timeline for testing procedure. (B–E Abscissa) Compound and dose administered in mg/kg. (B Ordinate) Total distance traveled during the 30 min session in cm. (C Ordinate) Total ambulatory counts in the 30 min Figure 6 (contd.) session. (D Ordinate) Total vertical counts during the 30 min session. (E Ordinate) Average speed during the 30 min session in cm/s (B–E) All bars represent the mean ± SEM *Denotes significant difference relative to the vehicle condition (open bar), defined as *p* < 0.05. (F–K Abscissa) Time in locomotor activity session in minutes. (F–H Ordinate) Average speed in cm/s (I–K Ordinate) Distance traveled in cm. (F–K) All points represent the mean ± SEM. Red bordered symbols denote significant difference relative to the vehicle condition (open symbols).

A well-known liability of KOR agonists is hypolocomotion.^113,114^ This hypolocomotion could result in false positives in assays of evoked antinociception such as WWTI.^115^ Thus, a locomotor activity assay was utilized to assess this end point in accordance with previous studies.^28^ Mice followed the testing schedule, as shown in Figure 6A. In brief, the day prior to testing, mice were placed in the open field chamber for acclimation. On the test day, they were injected with compounds **21**, **23**, NLF, or vehicle and immediately placed back into the open field chamber. Photo beam breaks then recorded the mouse’s locomotion over a 30 min test period.

Figure 6B–E shows the total distance traveled (cm), ambulatory counts, vertical counts, and average speed (cm/s) during the locomotor activity assay, while Figure 6F–H,I–K shows the average speed and distance traveled by time, respectively. There were slight dose-dependent trends for decreased locomotion with NLF administration. This is in agreement with published studies that NLF did not result in hypolocomotion at small doses but did so at large doses and similar to the trend observed with our previously published compound NMF.^28,47,49^ As previously discussed, due to the potency differences between compounds **21** and **23**, 0.1 mg/kg compound **21** may be more comparable to 0.05 mg/kg compound **23** and consequently 0.5 mg/kg compound **21** to 0.1 mg/kg compound **23**. Based on this, compound **21** seems to result in less sedative effects than compound **23** when compared at the same potency level. While high variance in the compound **21** group across all end points made it hard to conclude that this compound did not have any effects on locomotion, compound **21** did not result in average speeds significantly different from vehicle at any time point while both NLF and compound **23** did (Figure 6F–H). This may indicate the possibility for a potential augmented effect of activating both the KOR and DOR allowing for effective antinociception at nonsedative doses as hypothesized. A second potential explanation for diminished adverse effects may be biased agonism, similar to NLF. However, the concept of biased agonism is currently disputable and this observation may instead be due to underlying dose/efficacy effects. Further studies are warranted to verify both hypotheses.

Compounds **21** and **23** had similar *in vitro* profiles, and structurally they differ only in their C_6_ configuration. However, differences in these locomotor activity results may hearken back to their *in vivo* receptor selectivity. While compound **21** was similarly antagonized by both KOR and DOR antagonists, there was no significant effect of KOR antagonist pretreatment even on the lower dose 0.05 mg/kg compound **23** contrary to *in vitro* binding and functional studies. This may indicate that compound **23** is able to outcompete β-FNA at these doses and thus a greater involvement of the KOR leading to a potentially greater sedative effect when compared to the more balanced KOR/DOR compound **21**.

#### Abuse Liability *via* a Self-Administration Model

The potential for abuse liability is an important undesirable effect with the development of novel opioid ligands. To address this, self-administration assays were conducted in rats under a single-lever operant procedure according to published methods.^116^ In brief, rats were implanted with indwelling catheters as per published methods and trained to respond for drug (*i.e.*, fentanyl) on a fixed-ratio 5 (FR5) schedule of reinforcement.^116,117^ Then, the fentanyl syringe was replaced with a saline syringe in the syringe pump, and this switching occurred until rats were able to reliably distinguish the difference between fentanyl and saline according to established criteria (see Experimental Section). Once these criteria were met, the novel ligand (or fentanyl at different doses as a positive control) was inserted into the rotation as “test days”. Behavioral sessions occurred during daily, 2 h, blocks from approximately 9:30 AM–11:30 AM.

**Figure 7:**
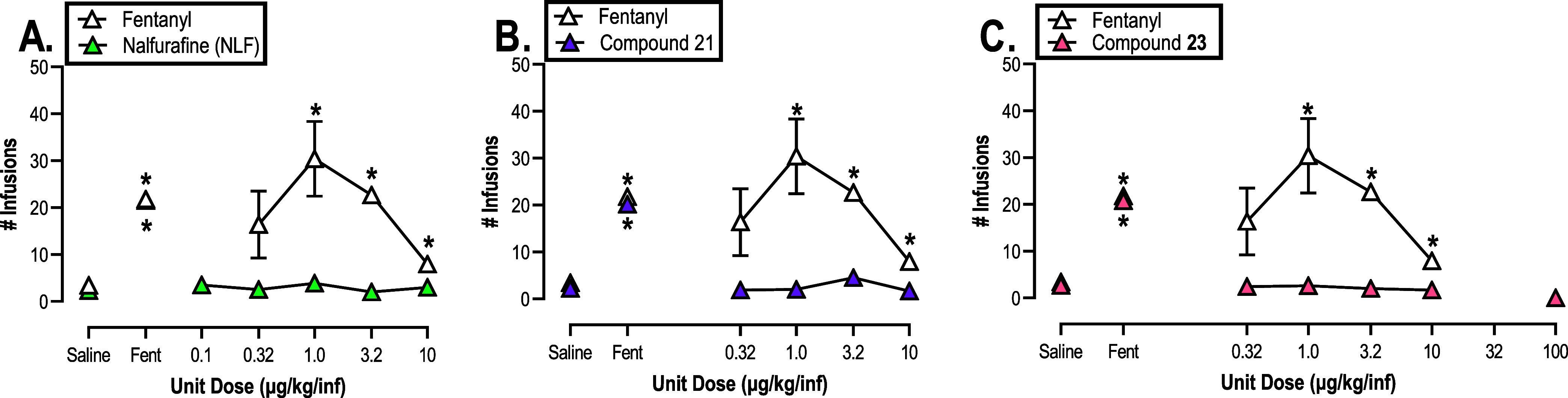
Self-administration of fentanyl and test compounds in male and female Sprague–Dawley rats (A: 2M, 6F; B: 2M, 6F; C: 3M, 6F [except 100 μg/kg/inf; 1M]). Ordinate: number of infusions earned under a FR5 schedule of reinforcement. Abscissa: intravenous unit dose of compound infusion in μg/kg/inf with compound being fentanyl (A–C), NLF (A), compound **21** (B) or compound **23** (C). Saline and “Fent” (Fentanyl) represent the mean ± SEM of all saline and 3.2 μg/kg/inf fentanyl days obtained as baselines between the testing days needed to acquire the respective dose–effect curves shown in (A–C). All points represent the mean ± SEM *Denotes significant difference relative to saline, defined as *p* < 0.05.

As a positive control, fentanyl self-administration was determined. Figure 7 shows that fentanyl functioned as a reinforcer yielding the expected inverted U-shaped dose–effect function. These results were in alignment with numerous previous self-administration studies.^116,118^ Supportive of a lack of abuse liability, NLF did not function as a reinforcer under a dose range similar to fentanyl. This is congruent with previous NLF studies in rats, rhesus monkeys and humans.^35,48,119,120^ Additionally, neither compound **21** nor compound **23** at any dose examined functioned as reinforcers because no dose maintained a significant number of infusions earned above saline. These results were in agreement with prior studies that interrogate the potential abuse liability of compounds that carry KOR agonist activity including our previously disclosed compound NMF.^28,121−123^

### ADMET

#### Drug Distribution

To examine the potential for these compounds to exert their effects on the central nervous system, a drug distribution study monitoring blood–brain barrier penetration was used. Briefly, following previously published methods, mice were subcutaneously injected with compound **21** or **23** (0.1 mg/kg) and sacrificed at various time points (5, 10, 30, 60 min) to facilitate collection of brain and blood samples. Plasma and brain homogenate were then analyzed *via* liquid chromatography with tandem mass spectrometry (LC-MS/MS) to allow calculation of brain-to-plasma ratios.^78^

**Figure 8:**
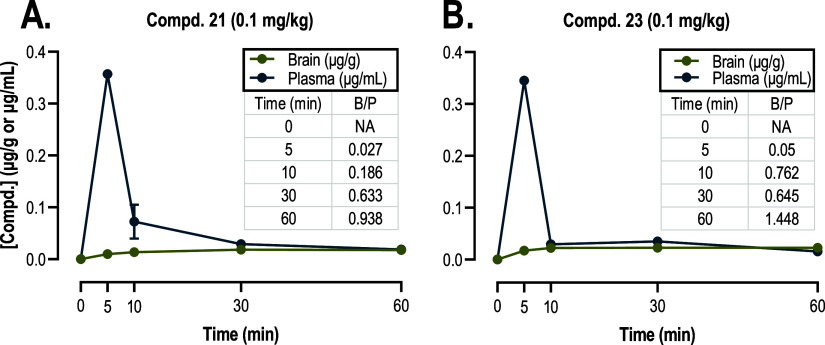
Brain and plasma distribution of (A) compound **21** and (B) compound **23** in male Swiss Webster mice (*n* = 3/time point). Ordinate: concentration of compound in brain (μg/g) and plasma (μg/mL). Abscissa: time post subcutaneous injection of ligands in min. All points represent the mean ± SEM.

Figure 8 shows that compounds **21** and **23** were detected in the plasma as early as 5 min after their s.c. administration, at which point the plasma concentration was the highest for both compounds. In addition, the brain-to-plasma concentration ratio progressively increased from 5 to 60 min for both compounds **21** and **23**. This indicated that the compounds not only penetrated the blood brain barrier but were also accumulating in the CNS over the time points tested. Additionally, it implies that they were not rapidly effluxed. These findings align with the warm water tail immersion studies, wherein compounds **21** and **23** exhibited a long duration of action (>3 h) (Figure 3). Conducting a comprehensive and long-term CNS distribution study would provide in-depth insight into the distribution of these compounds in the brain. Further, the initial spike in plasma concentration is expected considering pharmacokinetic principles wherein there was a high plasma concentration initially after injection followed by drug distribution and metabolism.

#### Metabolism Profile

Hepatic metabolic stability is an important factor to consider for both sustained durations of action and potential oral bioavailability. Thus, compounds can be optimized to avoid extensive first-pass hepatic metabolism, if necessary. As a result, compounds **21** and **23** as well as control compounds were incubated in human and rat liver S9 fractions which include both Phase I and Phase II metabolic enzymes. Assuming first order kinetics, the half-life of compounds **21** and **23** in human liver S9 fraction were over 120 min (Table 4). Meanwhile, the half-life of compounds **21** and **23** in rat liver S9 fraction were 69.7 min and over 120 min, respectively (Table 4). Their half-lives are longer than those of NMF which were determined to be over 60 min in human liver S9 fraction and 10 min in rat liver S9 fraction under the same conditions.^28^ These results hint at the potential for future oral dosing of these or potentially similar analogues. However, detailed *in vivo* pharmacokinetic studies are warranted.

**Table 4:** *In Vitro* Metabolism Study of Compounds **21** and **23** as Well as Control Compounds^a^

Compd.	*T*_1/2_—human (min)	CL_int_—human (μL/min/mg)	*T*_1/2_—rat (min)	CL_int_—rat (μL/min/mg)
**21**	>120	<5.8	69.7	10.1
**23**	>120	<5.8	>120	<5.8
clozapine	>120	<5.8	34.0	20.4
diclofenac	18.5	37.4	101.8	6.8
imipramine	102.7	22.8	20.3	113.7
propanolol	>120	<19.3	14.1	164.2
terfenadine	13.8	167.5	30.6	75.8

a Liver S9 fractions were used for both human and Sprague–Dawley rat determinations. Values shown represent the average of two individual replicates.

#### Cardiac Toxicity

The human ether-à-go-go-related-gene (hERG) codes for the pore-forming, alpha subunit of the potassium ion channel K_v_11.1.^124^ K_v_11.1 is crucial to normal cardiac function *via* mediating repolarizing currents to regulate heartbeat.^124^ Thus, inhibition of hERG decreases electrical current through these channels resulting in a lower heart rate and is undesirable under most circumstances.^124^ To assess this potential liability, compounds **21** and **23** as well as a control compound (verapamil) were evaluated concurrently in an automated patch-clamp hERG inhibition assay using CHO-hERG cells. Here, inhibition was assessed by dividing the test compound current amplitude by the baseline current amplitude at varying concentrations. The IC_50_ value of verapamil was 0.617 μM, while those of compounds **21** and **23** were 2.51 and 1.09 μM, respectively, which were similar to that of our previously reported, structurally similar analogue, NMF (6.7 μM).^28^ Therefore, compounds **21** and **23** seem unlikely to result in cardiac toxicity at potential therapeutic doses considering their high *in vivo* potencies.

## Conclusions

Despite the multitude of existing options available for the treatment of pain, they often fall short in providing adequate relief for some patients. This shortcoming has been one driver of the ongoing opioid crisis. Given this challenge, we aimed to design a nonaddictive pain medication. Nalfurafine (NLF), an approved antipruritic agent in Japan with an intriguing pharmacological profile with regard to pain, was chosen as a lead for further optimization through systematic SAR studies. Modifications to the structure of NLF at five positions have yielded a series of thirty-two analogues, 24 of which are reported herein. Characterization of these 24 analogues allowed identification as well as further confirmation of 3 main SAR trends: (1) the 3-hydroxyl group increases KOR binding affinity, is essential for MOR binding affinity, and additionally increases DOR binding affinity, (2) KOR *in vitro* efficacy is higher with R_2_ methyl group presence, and (3) the R_2_ methyl group may increase *in vivo* antinociceptive potency. Nine of the 24 analogues showed robust antinociception (≥80% MPE) in the WWTI assay, and six were more potent than morphine, while compounds **21** and **23** were approximately equipotent to more potent than both NLF and NMF.

Taking their *in vitro* and preliminary *in vivo* results together, two analogues (compounds **21** and **23**) were selected for further studies. Both compounds had durations of action longer than NLF, morphine and our previously disclosed compound NMF in the WWTI assay, penetrated the blood–brain barrier and did not function as reinforcers. However, compound **23** showed a dose-dependent decrease in locomotor activity across all end points, while compound **21** only resulted in a significant decrease in vertical counts at the largest dose tested. To address this potential sedative effect with compound **21**, future studies may consider incorporating alternative models to study these end points. Future studies may also probe for other typical liabilities associated with opioid use such as gastrointestinal transit, respiratory function, tolerance, and dependence. Altogether, our study suggests that KOR/DOR dual agonists may provide a route to analgesia while simultaneously avoiding the untoward effects of an agonist selective for either receptor alone.

## Experimental Section

### Chemical Synthesis

#### Reactions and Instrumentation

All reactions that involved moisture-sensitive reagents were conducted in oven-dried glassware under inert atmosphere maintained by nitrogen with anhydrous solvents. Chemicals and solvents were obtained from either Sigma-Aldrich or Combi-Blocks. Naltrexone was obtained as a free base through the NIDA Drug Supply Program. ^1^H NMR spectra were recorded at ambient temperature using a Bruker UltraShield Plus 400 MHz NMR (^13^C run at 100 MHz) spectrometer with IconNMR automation and B-ACS 60 autosampler and referenced to the solvent peak. Chemical shifts are expressed in ppm, and coupling constants, *J*, are in hertz (Hz). Peak multiplicities are abbreviated as singlet, s; doublet, d; triplet, t; quartet, q; pentet, p; and multiplet, m; with two letters next to each other representing the first of the second *e.g.*, dd, doublet of doublets. Mass spectrometry analysis was performed on a PerkinElmer AxION 2 TOF MS. Melting points were determined using an OptiMelt automated melting point apparatus. Flash column chromatography was performed with silica gel columns (230–400 mesh, Merck). Purity as determined by high performance liquid chromatography was resolved on a Waters Arc HPLC system using XBridge C_18_ 3.5 μm (4.6 × 50 mm) column. All analyses were conducted at ambient temperatures with a flow rate of 0.2 mL/min and an isocratic mobile phase of 80% acetonitrile/20% water with 0.1% trifluoroacetic acid. The UV detector was set up at 210 nm. Compound purities were calculated as the percentage peak area of the analyzed compound, and retention times were presented in minutes. The purity of all newly synthesized compounds was identified as ≥95% by HPLC.

#### General Procedures

##### General Procedure 1: Acyl Chloride Formation

The acid (3 equiv*) was dissolved in anhydrous CH_2_Cl_2_ (2 mL) in a predried 50 mL round-bottom flask and stirred approximately 12–18 h with 4 Å molecular sieves to predry. After this time, oxalyl chloride (4 equiv) and catalytic dimethylformamide (2 drops) were added while maintaining the reaction mixture under inert conditions using a N_2_ balloon. The reaction was allowed to stir at room temperature for two h, after which time a very small portion was removed, dried thoroughly *in vacuo*, and compared to the starting material *via*^1^H NMR. When NMR revealed completion, the solvent was removed *in vacuo* (with molecular sieves and stir bar remaining inside the round-bottom flask). The acid chloride (dried contents of the flask) was then used immediately for the amidation of a secondary amine in general procedure 2 without further purification.

*The base of 1 equiv is the amine in general procedure 2.

##### General Procedure 2: Amidation of a Secondary Amine

Acid chloride (as previously prepared according to general procedure 1) was dissolved in anhydrous CH_2_Cl_2_ (2 mL) without further purification, and 4 Å molecular sieves were added to the round-bottom flask on an ice water bath under N_2_ atmosphere. The amine (1 equiv) was added in one portion followed by the dropwise addition of triethylamine (4 equiv). The reaction was allowed to come to room temperature with stirring. After approximately 3 h, reaction completion was confirmed by TLC (30:1 CH_2_Cl_2_/MeOH w/NH_3_·H_2_O). The reaction mixture was then filtered through Celite and concentrated *in vacuo*. If R_1_ = H, purification was performed by flash column chromatography with CH_2_Cl_2_/MeOH (1% NH_3_·H_2_O) as the eluent, and purified compounds were then subjected to general procedure 5. If R_1_ = OH, compounds were used directly in general procedure 4 without further purification.

##### General Procedure 3: for Amidation of a Primary Amine

Carboxylic acid (1.5 equiv) was dissolved in anhydrous dimethylformamide (2 mL) in a predried 50 mL round-bottom flask and stirred with 4 Å molecular sieves approximately 12–18 h to predry. After this time, 1-ethyl-3-(3-(dimethylamino)propyl)carbodiimide (EDCI, 2 equiv) and hydroxybenzotriazole (HOBt, 2 equiv + 20%) were added to the round-bottom flask on an ice water bath while maintaining the reaction mixture under inert conditions using a N_2_ balloon. This was immediately followed by the dropwise addition of triethylamine (5 equiv) and fresh 4 Å molecular sieves to the round-bottom flask. The reaction was allowed to come to room temperature while stirring for 2 h after which time the amine (1 equiv) was added and the reaction was left to stir monitoring by TLC (30:1 CH_2_Cl_2_/MeOH w/NH_3_·H_2_O) until completion was reached. At this time, it was filtered over Celite and concentrated *in vacuo*. Purification of 3-dehydroxy compounds was performed by flash column chromatography with CH_2_Cl_2_/MeOH (1% NH_3_·H_2_O) as the eluent. Purified compounds were then subjected to general procedure 5. Compounds originating from starting materials with a 3-OH group were used directly in general procedure 4 without further purification.

##### General Procedure 4: Deprotection of 3-OH (Only when R_1_ = OH)

The presumed disubstituted intermediate was dissolved in anhydrous methanol (3 mL) under N_2_ atmosphere and an ice–water bath was applied. Potassium carbonate (10 equiv) was added in one portion, and the reaction was allowed to return to room temperature with stirring. After approximately 2 days to 1 week when TLC (30:1 CH_2_Cl_2_/MeOH w/NH_3_·H_2_O) indicated completion, the reaction was filtered over Celite and concentrated *in vacuo*. Purification was performed by flash column chromatography with CH_2_Cl_2_/MeOH (1% NH_3_·H_2_O) as the eluent. Purified free bases were then subjected to general procedure 5.

##### General Procedure 5: Hydrochloride Salt Formation

The final compound as a free base (1 equiv) was dissolved in anhydrous methanol under N_2_ atmosphere, and HCl in methanol solution (2 equiv) was added dropwise after application of an ice–water bath. After stirring for 30 min, 15 mL of diethyl ether was added, which precipitated out a fine powder in most cases. After 2 h, the solid was filtered by vacuum and fully dried.

#### Final Compounds

##### 17-Cyclopropylmethyl-4,5α-epoxy-6α-[3′-(furan-3″-yl)*N*-methylpropanamido]-14β-hydroxymorphinan Hydrochloride (**1**)

The title compound was prepared following general procedures 1, 2, and 5 as a light-yellow solid in 26.4% yield. Hydrochloride salt: ^1^H NMR (400 MHz, DMSO-*d*_6_) δ 8.86 (br s, 1H, exchangeable), 7.55 (s, 1H), 7.46 (s, 1H), 7.18 (t, *J* = 7.8 Hz, 1H), 6.77 (d, *J* = 7.7 Hz, 1H), 6.68 (d, *J* = 7.9 Hz, 1H). 6.43 (s, 1H), 6.30 (s, 1H, exchangeable), 5.07–4.94 (m, 1H), 4.64 (d, *J* = 3.0 Hz, 1H), 3.95 (d, *J* = 6.2 Hz, 1H), 3.46 (d, *J* = 20.2, 2H), 3.23 (d, *J* = 7.0 Hz, 1H), 3.06–2.96 (m, 3H), 2.80 (d, *J* = 37.8 Hz, 3H), 2.73–2.57 (m, 5H), 2.45 (m, 1H), 1.95 (dd, *J* = 16.8, 8.6 Hz, 1H), 1.64–1.50 (m, 2H), 1.03 (t, *J* = 8 Hz, 1H), 1.09–1.07 (m, 1H), 0.73–0.66 (m, 1H), 0.65–0.57 (m, 1H), 0.53–0.45 (m, 1H), 0.44–0.36 (m, 1H). ^13^C NMR (100 MHz, DMSO-*d*_6_) δ 171.7, 159.2, 142.8, 139.0, 132.6, 129.9, 127.5, 124.2, 118.6, 111.3, 105.9, 89.2, 68.9, 61.0, 57.1, 48.5, 48.4, 45.0, 44.9, 33.5, 31.2, 29.8, 24.1, 19.8, 17.6, 5.6, 5.1, 2.5. HRMS (*m*/*z*) [M + H]^+^ calcd for C_28_H_34_N_2_O_4_, 463.2491; found, 463.2578, MMA = 2.81 ppm, mp 186 °C.

##### 17-Cyclopropylmethyl-4,5α-epoxy-6α-[3′-(furan-3″-yl)propanamido]-14β-hydroxymorphinan Hydrochloride (**2**)

The title compound was prepared following general procedures 3 and 5 as a light-yellow solid in 52.5% yield. Hydrochloride salt: ^1^H NMR (400 MHz, DMSO-*d*_6_) δ 8.87 (br s, 1H, exchangeable), 7.79 (d, *J* = 7.9 Hz, 1H, exchangeable), 7.54 (s, 1H), 7.43 (s, 1H), 7.17 (t, *J* = 7.7 Hz, 1H), 6.74 (d, *J* = 7.6 Hz, 1H), 6.67 (d, *J* = 7.9 Hz, 1H), 6.38 (s, 1H, exchangeable), 6.28 (br s, 1H), 4.61 (d, *J* = 3.5 Hz, 1H), 4.46–4.37 (m, 1H), 3.92 (d, *J* = 6.4 Hz, 1H), 3.48–3.43 (m, 2H), 3.31–3.26 (m, 1H), 3.15 (dd, *J* = 20.1, 6.9 Hz, 1H), 3.05 (d, *J* = 12.3 Hz, 1H), 2.95 (m, 1H), 2.76–2.67 (m, 1H), 2.62 (t, *J* = 7.5 Hz, 2H), 2.39 (t, *J* = 7.4 Hz, 2H), 1.87 (dd, *J* = 16.6, 8.4 Hz, 1H), 1.61 (d, *J* = 12.0 Hz, 1H), 1.38 (m, 2H), 1.07 (br s, 1H), 0.87 (t, *J* = 11.3 Hz, 1H), 0.74–0.58 (m, 2H), 0.53–0.38 (m, 2H). ^13^C NMR (100 MHz, DMSO-*d*_6_) δ 171.0, 159.3, 142.9, 138.9, 132.6, 129.7, 127.5, 124.1, 118.4, 111.2, 106.2, 87.6, 69.2, 60.9, 57.0, 45.0, 44.8, 44.4, 35.3, 30.1, 29.1, 24.1, 20.4, 19.5, 5.6, 5.1, 2.5. HRMS (*m*/*z*) [M + H]^+^ calcd for C_27_H_32_N_2_O_4_, 449.2435; found, 449.2419, MMA = 3.56 ppm, mp 175 °C.

##### 17-Cyclopropylmethyl-4,5α-epoxy-6β-[3′-(furan-3″-yl)-*N*-methylpropanamido]-14β-hydroxymorphinan Hydrochloride (**3**)

The title compound was prepared following general procedures 1, 2, and 5 as a yellow solid in 47.6% yield. Hydrochloride salt: ^1^H NMR (400 MHz, DMSO-*d*_6_) δ 8.83 (br s, 1H, exchangeable), 7.52 (d, *J* = 14.7 Hz, 1H), 7.37 (d, *J* = 49.1 Hz, 1H), 7.22–7.15 (m, 1H), 6.89–6.81 (m, 1H), 6.72 (dd, *J* = 19.1, 7.3 Hz, 1H), 6.41 (s, 1H), 6.24 (s, 0.5H, exchangeable), 5.75 (s, 0.5H, exchangeable), 4.82 (t, *J* = 9.0 Hz, 1H), 3.89 (s, 1H), 3.48–3.35 (m, 2H), 3.18–3.14 (m, 1H), 3.07–3.04 (m, 1H), 2.98 (s, 1H), 2.89 (s, 1H), 2.84 (s, 2H), 2.63–2.52 (m, 3H), 2.47–2.40 (m, 1H), 2.31 (t, *J* = 7.4 Hz, 1H), 2.08 (t, *J* = 16.5 Hz, 1H), 1.73 (d, *J* = 12.2 Hz, 1H), 1.40 (dd, *J* = 20.7, 10.8 Hz, 3H), 1.30–1.16 (m, 1H), 1.08 (d, *J* = 3.9 Hz, 1H), 0.72–0.57 (m, 2H), 0.47 (dd, *J* = 33.3, 3.3 Hz, 2H). ^13^C NMR (100 MHz, DMSO-*d*_6_) δ 171.7, 171.3, 155.9, 155.3, 142.8, 138.8, 131.3, 129.5, 128.6, 124.1, 119.2, 118.6, 111.1, 87.9, 69.5, 61.2, 56.7, 54.8, 46.1, 45.1, 32.5, 30.2, 26.9, 23.6, 20.0, 5.7, 5.1, 2.6. HRMS (*m*/*z*) [M + H]^+^ calcd for C_28_H_34_N_2_O_4_, 463.2591; found, 463.2580, MMA = 2.37 ppm, mp 197 °C.

##### 17-Cyclopropylmethyl-4,5α-epoxy-6β-[3′-(furan-3″-yl)propanamido]-14β-hydroxymorphinan Hydrochloride (**4**)

The title compound was prepared following general procedures 3 and 5 as a light-yellow solid in 40.5% yield. Hydrochloride salt: ^1^H NMR (400 MHz, DMSO-*d*_6_) δ 8.85 (br s, 1H, exchangeable), 8.13 (d, *J* = 8.1 Hz, 1H, exchangeable), 7.55 (t, *J* = 1.6 Hz, 1H), 7.42 (s, 1H), 7.18 (t, *J* = 7.8 Hz, 1H), 6.83 (d, *J* = 7.8 Hz, 1H), 6.74 (d, *J* = 7.9 Hz, 1H), 6.36 (s, 1H), 6.17 (br s, 1H, exchangeable), 4.53 (d, *J* = 7.9 Hz, 1H), 3.86 (d, *J* = 4.8 Hz, 1H), 3.46–3.41 (m, 3H), 3.20–3.14 (m, 1H), 3.02 (d, *J* = 7.1 Hz, 1H), 2.88–2.80 (m, 1H), 2.60 (t, *J* = 7.4 Hz, 2H), 2.41 (d, *J* = 8.8 Hz, 2H), 2.34–2.28 (m, 2H), 1.76–1.64 (m, 2H), 1.44 (t, *J* = 10.4 Hz, 2H), 1.33 (m, 1H), 1.09 (t, *J* = 7.0 Hz, 1H), 0.72–0.65 (m, 1H), 0.63–0.55 (m, 1H), 0.49 (dt, *J* = 9.3, 4.6 Hz, 1H), 0.42 (dt, *J* = 9.2, 4.5 Hz, 1H). ^13^C NMR (100 MHz, DMSO-*d*_6_) δ 171.0, 155.8, 142.9, 138.9, 131.2, 129.5, 128.3, 124.0, 118.7, 111.2, 108.9, 90.3, 69.6, 61.6, 56.7, 50.4, 46.2, 44.9, 35.9, 29.4, 27.2, 23.7, 23.6, 20.3, 5.7, 5.1, 2.6. HRMS (*m*/*z*) [M + H]^+^ calcd for C_27_H_32_N_2_O_4_, 449.2435; found, 449.2415, MMA = 4.45 ppm, mp 206 °C.

##### 17-Cyclopropylmethyl-3,14β-dihydroxy-4,5α-epoxy-6α-[3′-(furan-3″-yl)-*N*-methylpropanamido]morphinan Hydrochloride (**5**)

The title compound was prepared following general procedures 1, 2, 4, and 5 as a white solid in 24.8% yield. Hydrochloride salt: ^1^H NMR (400 MHz, DMSO-*d*_6_) δ 9.29 (s, 1H, exchangeable), 8.79 (br s, 1H, exchangeable), 7.55 (d, *J* = 1.6 Hz, 1H), 7.47 (s, 1H), 6.71 (d, *J* = 8.1 Hz, 1H), 6.58 (d, *J* = 8.1 Hz, 1H), 6.44 (s, 1H), 6.20 (s, 1H, exchangeable), 5.03–4.93 (m, 1H), 4.62 (d, *J* = 3.9 Hz, 1H), 3.88 (d, *J* = 6.4 Hz, 1H), 3.30–3.22 (m, 2H), 3.11 (d, *J* = 6.7 Hz, 1H), 3.03–3.01 (m, 1H), 2.91 (s, 1H), 2.84 (d, *J* = 36.0 Hz, 3H), 2.71–2.59 (m, 5H), 2.44–2.40 (m, 1H), 1.96–1.87 (m, 1H), 1.64–1.52 (m, 2H), 1.33 (t, *J* = 10.7 Hz, 1H), 1.14–1.07 (m, 1H), 1.04 (s, 1H), 0.71–0.58 (m, 2H), 0.49–0.37 (m, 2H). ^13^C NMR (100 MHz, DMSO-*d*_6_) δ 171.6, 142.9, 139.1, 124.3, 122.0, 119.3, 118.3, 118.1, 117.9, 111.5, 111.4, 110.2, 68.9, 68.8, 61.2, 57.0, 48.5, 45.6, 45.0, 33.5, 31.2, 29.9, 29.8, 23.4, 19.9, 5.7, 4.9, 2.5. HRMS (*m*/*z*) [M + H]^+^ calcd for C_28_H_34_N_2_O_5_, 479.2540; found, 479.2523, MMA = 3.55 ppm, mp 297 °C.

##### 17-Cyclopropylmethyl-3,14β-dihydroxy-4,5α-epoxy-6α-[3′-(furan-3″-yl)propanamido]morphinan Hydrochloride (**6**)—Compound Previously Published in Ref (81)

The title compound was prepared following general procedures 3, 4, and 5 as a white powder in 51% yield. Hydrochloride salt: ^1^H NMR (400 MHz, DMSO-*d*_6_) δ 9.19 (s, 1H), 8.83 (s, 1H), 7.69 (d, *J* = 8 Hz, 1H), 7.54 (m, 1H), 7.44 (m, 1H), 6.72 (d, *J* = 8 Hz, 1H), 6.55 (d, *J* = 8 Hz, 1H), 6.39 (m, 1H), 6.24 (s, 1H), 4.58 (d, *J* = 4 Hz, 1H), 4.43–4.38 (m, 1H), 3.90 (d, *J* = 4 Hz, 2H), 3.26 (m, 2H), 3.05 (m, 2H), 2.94 (m, 1H), 2.71 (m, 1H), 2.63 (t, 2H), 2.43 (m, 3H), 1.85 (m, 1H), 1.61 (m, 1H), 1.39 (m, 2H), 0.92 (m, 1H), 0.63 (m, 2H), 0.44 (m, 2H). ^13^C NMR (100 MHz, DMSO-*d*_6_) δ 170.9, 146.0, 142.9, 138.8, 128.7, 124.1, 122.0, 119.0, 118.2, 111.2, 87.5, 69.3, 64.9, 61.0, 57.0, 45.1, 44.8, 35.4, 30.1, 29.2, 23.4, 20.4, 19.7, 15.1, 5.7, 5.1, 2.5. HRMS (*m*/*z*) [M + H]^+^ calcd for C_27_H_32_N_2_O_5_, 465.2384; found, 465.2403, MMA = 4.08 ppm, 192 °C.

##### 17-Cyclopropylmethyl-3,14β-dihydroxy-4,5α-epoxy-6β-[3′-(furan-3″-yl)-*N*-methylpropanamido]morphinan Hydrochloride (**7**)

The title compound was prepared following general procedures 1, 2, 4, and 5 as a white solid in 53.1% yield. Hydrochloride salt: ^1^H NMR (400 MHz, DMSO-*d*_6_) δ 9.54 (d, *J* = 112 Hz, 1H, exchangeable), 8.79 (br s, 1H, exchangeable), 7.50 (dt, *J* = 21.8, 1.5 Hz, 1H), 7.38 (d, *J* = 58.0 Hz, 1H), 6.77–6.62 (m, 2H), 6.39 (d, *J* = 22.8 Hz, 1H, exchangeable), 6.28 (s, 1H), 4.80 (dd, *J* = 28.0, 8.2 Hz, 1H), 4.10 (s, 0.3H), 3.84 (d, *J* = 5.7 Hz, 1H), 3.47 (ddd, *J* = 12.4, 8.0, 4.2 Hz, 0.7H), 3.41–3.33 (m, 1H), 3.08–3.01 (m, 2H), 2.98 (s, 1H), 2.87 (d, *J* = 11.8 Hz, 1H), 2.83 (s, 2H), 2.75–2.60 (m, 1H), 2.57 (d, *J* = 8.0 Hz, 1H), 2.47–2.42 (m, 2H), 2.34–2.26 (m, 1H), 2.12–2.03 (m, 1H), 1.70 (d, *J* = 13.5 Hz, 1H), 1.51–0.97 (m, 6H), 0.71–0.65 (m, 1H), 0.62–0.56 (m, 1H), 0.53–0.47 (m, 1H), 0.44–0.38 (m, 1H). ^13^C NMR (100 MHz, DMSO-*d*_6_) δ 171.5, 142.7, 141.5, 138.9, 129.9, 124.1, 120.7, 119.8, 117.8, 111.5, 111.2, 87.6, 69.6, 61.3, 56.9, 56.6, 46.4, 45.8, 32.3, 30.3, 27.9, 27.0, 22.9, 21.7, 20.1, 5.7, 5.1, 2.6. HRMS (*m*/*z*) [M + H]^+^ calcd for C_28_H_34_N_2_O_5_, 479.2540; found, 479.2516, MMA = 5.01 ppm, mp 229 °C.

##### 17-Cyclopropylmethyl-3,14β-dihydroxy-4,5α-epoxy-6β-[3′-(furan-3″-yl)propanamido]morphinan Hydrochloride (**8**)—Compound Previously Published in Ref (81)

The title compound was prepared following general procedures 3, 4, and 5 as a yellow powder in 57% yield. Hydrochloride salt: ^1^H NMR (400 MHz, DMSO-*d*_6_) δ 10.03 (s, 1H), 9.22 (s, 1H), 8.84 (s, 1H), 8.28 (s, 1H), 7.89–7.87 (m, 1H), 7.74 (t, *J* = 1.64 Hz, 1H), 6.93 (s, 1H), 6.72 (d, *J* = 8 Hz, 1H), 6.57 (d, *J* = 8 Hz, 1H), 6.31 (m, 1H), 4.72 (d, *J* = 3.84 Hz, 1H), 4.55 (m, 1H), 3.90 (m, 1H), 2.95 (m, 1H), 2.72 (m, 2H), 1.91 (m, 1H), 1.63 (m, 1H), 1.45 (m, 2H), 1.09 (m, 3H), 0.65 (m, 2H), 0.44 (m, 2H). ^13^C NMR (100 MHz, DMSO-*d*_6_) δ 171.0, 142.9, 142.1, 141.3, 138.9, 129.6, 123.9, 120.5, 119.1, 117.9, 111.2, 89.9, 69.7, 61.7, 56.7, 50.6, 46.5, 45.5, 35.9, 29.3, 27.3, 23.6, 22.9, 20.3, 5.7, 5.0, 2.6. HRMS (*m*/*z*) [M + H]^+^ calcd for C_27_H_32_N_2_O_5_, 465.2384; found, 465.2400, MMA = 3.44 ppm, mp 185 °C.

##### 17-Cyclopropylmethyl-4,5α-epoxy-6α-[3′-(furan-2″-yl)*N*-methylpropanamido]-14β-hydroxymorphinan Hydrochloride (**9**)

The title compound was prepared following general procedures 1, 2, and 5 as a yellow solid in 16% yield. Hydrochloride salt: ^1^H NMR (400 MHz, DMSO-*d*_6_) δ 8.86 (br s, 1H, exchangeable), 7.50 (s, 1H), 7.18 (t, *J* = 7.8 Hz, 1H), 6.77 (d, *J* = 7.7 Hz, 1H), 6.69 (t, *J* = 8.1 Hz, 1H), 6.35–6.28 (m, 2H), 6.12 (s, 1H, exchangeable), 5.03–4.79 (m, 1H), 4.63–4.47 (m, 1H), 3.94 (d, *J* = 6.3 Hz, 1H), 3.48–3.43 (m, 2H), 3.28–3.16 (m, 2H), 3.07–2.88 (m, 3H), 2.85 (s, 3H), 2.83–2.65 (m, 4H), 2.44 (d, *J* = 8.7 Hz, 1H), 2.01–1.89 (m, 1H), 1.65–1.49 (m, 2H), 1.34 (t, *J* = 9.9 Hz, 1H), 1.08 (d, *J* = 7.0 Hz, 2H), 0.74–0.57 (m, 2H), 0.52–0.37 (m, 2H). ^13^C NMR (100 MHz, DMSO-*d*_6_) δ 171.2, 159.2, 154.9, 141.2, 132.7, 130.0, 127.5, 118.7, 110.4, 105.9, 105.1, 89.3, 68.9, 61.0, 57.0, 48.5, 45.0, 44.9, 31.4, 31.2, 29.9, 29.8, 24.1, 23.0, 17.6, 5.7, 5.2, 2.6. HRMS (*m*/*z*) [M + H]^+^ calcd for C_28_H_34_N_2_O_4_, 463.2591; found, 463.2578, MMA = 2.81 ppm, mp 171 °C.

##### 17-Cyclopropylmethyl-4,5α-epoxy-6α-[3′-(furan-2″-yl)propanamido]-14β-hydroxymorphinan Hydrochloride (**10**)

The title compound was prepared following general procedures 3 and 5 as a white solid in 38.9% yield. Hydrochloride salt: ^1^H NMR (400 MHz, DMSO-*d*_6_) δ 8.87 (br s, 1H, exchangeable), 7.87 (d, *J* = 8.1 Hz, 1H, exchangeable), 7.49 (d, *J* = 1.1 Hz, 1H), 7.17 (t, *J* = 7.8 Hz, 1H), 6.74 (d, *J* = 7.7 Hz, 1H), 6.67 (d, *J* = 7.9 Hz, 1H), 6.34 (dd, *J* = 3.0, 1.9 Hz, 1H), 6.27 (br s, 1H, exchangeable), 6.09 (d, *J* = 3.1 Hz, 1H), 4.60 (d, *J* = 3.7 Hz, 1H), 4.43 (ddd, *J* = 16.7, 8.0, 4.0 Hz, 1H), 3.91 (d, *J* = 6.7 Hz, 1H), 3.48–3.43 (m, 1H), 3.29–3.24 (m, 1H), 3.19–3.12 (m, 1H), 3.04 (d, *J* = 11.2 Hz, 1H), 2.98–2.92 (m, 1H), 2.83 (t, *J* = 7.5 Hz, 2H), 2.68 (dd, *J* = 14.1, 7.7 Hz, 1H), 2.46 (t, *J* = 7.6 Hz, 3H), 1.86 (dt, *J* = 15.5, 9.5 Hz, 1H), 1.61 (d, *J* = 10.7 Hz, 1H), 1.41–1.35 (m, 2H), 1.09 (t, *J* = 7.0 Hz, 1H), 0.93–0.82 (m, 1H), 0.73–0.57 (m, 2H), 0.52–0.37 (m, 2H). ^13^C NMR (100 MHz, DMSO-*d*_6_) δ 170.6, 159.3, 154.8, 141.3, 132.6, 129.8, 127.6, 118.5, 110.4, 106.3, 105.1, 87.6, 69.2, 60.9, 57.0, 45.0, 44.8, 44.4, 33.2, 30.1, 29.1, 24.2, 23.5, 19.5, 5.7, 5.2, 2.6. HRMS (*m*/*z*) [M + H]^+^ calcd for C_27_H_32_N_2_O_4_, 449.2435; found, 449.2419, MMA = 3.56 ppm, mp 189 °C.

##### 17-Cyclopropylmethyl-4,5α-epoxy-6β-[3′-(furan-2″-yl)-*N*-methylpropanamido]-14β-hydroxymorphinan Hydrochloride (**11**)

The title compound was prepared following general procedures 1, 2, and 5 as a yellow solid in 20.3% yield (average). Hydrochloride salt: ^1^H NMR (400 MHz, DMSO-*d*_6_) δ 8.83 (s, 1H, exchangeable), 7.48 (d, *J* = 9.2 Hz, 1H), 7.21–7.14 (m, 1H), 6.85 (dd, *J* = 14.7, 7.7 Hz, 1H), 6.70 (t, *J* = 8.1 Hz, 1H), 6.41–6.29 (m, 2H), 6.12–5.95 (m, 1H, exchangeable), 4.82 (t, *J* = 9.3 Hz, 1H), 3.88 (s, 1H), 3.43 (s, 3H), 3.26 (s, 1H), 3.21–3.11 (m, 2H), 3.06 (dd, *J* = 13.7, 8.4 Hz, 2H), 2.98 (s, 1H), 2.84 (s, 2H), 2.82–2.62 (m, 3H), 2.41 (dd, *J* = 17.2, 8.7 Hz, 2H), 2.08 (s, 1H), 1.73 (d, *J* = 12.1 Hz, 1H), 1.43 (d, *J* = 12.3 Hz, 2H), 1.19 (t, *J* = 7.3 Hz, 1H), 0.73–0.55 (m, 2H), 0.52–0.39 (m, 2H). ^13^C NMR (100 MHz, DMSO-*d*_6_) δ 154.9, 141.2, 129.5, 119.2, 110.2, 105.1, 104.9, 99.5, 87.2, 73.6, 69.6, 69.5, 64.9, 61.2, 56.7, 46.2, 45.5, 45.2, 30.2, 27.9, 23.6, 23.2, 15.1, 8.5, 5.7, 5.1, 2.6, 0.1. HRMS (*m*/*z*) [M + H]^+^ calcd for C_28_H_34_N_2_O_4_, 463.2591; found, 463.2570, MMA = 4.53 ppm, mp 173 °C.

##### 17-Cyclopropylmethyl-4,5α-epoxy-6β-[3′-(furan-2″-yl)propanamido]-14β-hydroxymorphinan Hydrochloride (**12**)

The title compound was prepared following general procedures 3 and 5 as a light-yellow solid in 22.1% yield. Hydrochloride salt: ^1^H NMR (400 MHz, DMSO-*d*_6_) δ 8.86 (br s, 1H, exchangeable), 8.18 (d, *J* = 7.9 Hz, 1H, exchangeable), 7.49 (s, 1H), 7.17 (t, *J* = 7.8 Hz, 1H), 6.83 (d, *J* = 7.7 Hz, 1H), 6.74 (d, *J* = 7.8 Hz, 1H), 6.34 (d, *J* = 1.5 Hz, 1H), 6.19 (br s, 1H, exchangeable), 6.08 (d, *J* = 2.4 Hz, 1H), 4.54 (d, *J* = 7.9 Hz, 1H), 3.87 (d, *J* = 5.0 Hz, 1H), 3.48–3.37 (m, 3H), 3.17 (dd, *J* = 19.7, 5.7 Hz, 1H), 3.03 (d, *J* = 6.7 Hz, 1H), 2.81 (t, *J* = 7.4 Hz, 3H), 2.40 (t, *J* = 7.6 Hz, 4H), 1.71 (d, *J* = 12.5 Hz, 2H), 1.51–1.38 (m, 2H), 1.32 (t, *J* = 13.5 Hz, 1H), 1.09 (t, *J* = 6.9 Hz, 1H), 0.64 (ddd, *J* = 33.9, 8.1, 4.6 Hz, 2H), 0.46 (ddd, *J* = 35.6, 8.7, 4.3 Hz, 2H). ^13^C NMR (100 MHz, DMSO-*d*_6_) δ 170.5, 155.8, 154.6, 141.2, 131.2, 129.5, 128.3, 118.7, 110.3, 108.9, 105.0, 90.3, 69.6, 64.9, 61.5, 56.7, 50.4, 46.2, 44.9, 33.7, 29.3, 27.2, 23.6, 23.4, 5.7, 5.1, 2.6. HRMS (*m*/*z*) [M + H]^+^ calcd for C_27_H_32_N_2_O_4_, 449.2435; found, 449.2434, MMA = 0.22 ppm, mp 185 °C.

##### 17-Cyclopropylmethyl-3,14β-dihydroxy-4,5α-epoxy-6α-[3′-(furan-2″-yl)-*N*-methylpropanamido]morphinan Hydrochloride (**13**)

The title compound was prepared following general procedures 1, 2, 4, and 5 as a white solid in 25.2% yield. Hydrochloride salt: ^1^H NMR (400 MHz, DMSO-*d*_6_) δ 9.29 (s, 1H, exchangeable), 8.80 (br s, 1H, exchangeable), 7.52 (t, *J* = 5.8 Hz, 1H), 6.72 (d, *J* = 8.1 Hz, 1H), 6.58 (d, *J* = 8.2 Hz, 1H), 6.43 (s, 0.2H, exchangeable), 6.36 (dd, *J* = 5.8, 2.8 Hz, 1H), 6.22 (s, 0.8H, exchangeable), 6.12 (d, *J* = 2.9 Hz, 1H), 4.98 (dt, *J* = 7.1, 3.2 Hz, 1H), 4.69 (dd, *J* = 61.5, 3.0 Hz, 1H), 3.90 (d, *J* = 6.4 Hz, 1H), 3.43–3.32 (m, 1H), 3.29–3.22 (m, 1H), 3.15–3.06 (m, 1H), 3.06–3.00 (m, 1H), 2.92 (d, *J* = 5.4 Hz, 1H), 2.90 (s, 2.5H), 2.86 (d, *J* = 7.9 Hz, 2H), 2.81 (s, 0.5H), 2.71–2.66 (m, 2H), 2.46–2.38 (m, 1H), 1.92 (dt, *J* = 13.5, 9.4 Hz, 1H), 1.64–1.52 (m, 2H), 1.48–1.20 (m, 2H), 1.18–1.02 (m, 2H), 0.65 (ddd, *J* = 28.8, 7.9, 4.4 Hz, 2H), 0.50–0.37 (m, 2H). ^13^C NMR (100 MHz, DMSO-*d*_6_) δ 171.1, 154.9, 145.7, 141.2, 139.0, 128.8, 122.0, 119.3, 118.1, 110.4, 105.1, 89.0, 68.9, 61.1, 57.0, 48.5, 45.6, 45.2, 31.4, 31.2, 30.0, 29.8, 23.4, 23.0, 17.8, 5.6, 5.1, 2.5. HRMS (*m*/*z*) [M + H]^+^ calcd for C_28_H_34_N_2_O_5_, 479.2540; found, 479.2529, MMA = 2.30 ppm, mp 285 °C.

##### 17-Cyclopropylmethyl-3,14β-dihydroxy-4,5α-epoxy-6α-[3′-(furan-2″-yl)propanamido]morphinan Hydrochloride (**14**)—Compound Previously Published in Ref (81)

The title compound was prepared following general procedures 3, 4, and 5 as a white solid in 47.3% yield. Hydrochloride salt: ^1^H NMR (400 MHz, DMSO-*d*_6_) δ 9.15 (s, 1H, exchangeable), 8.79 (br s, 1H, exchangeable), 7.71 (d, *J* = 7.5 Hz, 1H, exchangeable), 7.49 (s, 1H), 6.71 (d, *J* = 8.1 Hz, 1H), 6.56 (d, *J* = 8.1 Hz, 1H), 6.33 (s, 1H), 6.19 (s, 1H, exchangeable), 6.10 (s, 1H), 4.59 (s, 1H), 4.41 (s, 1H), 3.87 (d, *J* = 5.3 Hz, 1H), 3.36 (d, *J* = 7.5 Hz, 1H), 3.26 (s, 2H), 3.04 (d, *J* = 13.8, Hz, 2H), 2.94 (s, 1H), 2.84 (t, *J* = 7.1 Hz, 2H), 2.75–2.68 (m, 1H), 2.46 (s, 1H), 1.88–1.79 (m, 1H), 1.61 (d, *J* = 13.2 Hz, 1H), 1.45–1.34 (m, 2H), 1.11–1.05 (m, 2H), 0.97–0.87 (m, 1H), 0.68–0.61 (m, 2H), 0.47–0.40 (m, 2H). ^13^C NMR (100 MHz, DMSO-*d*_6_) δ 170.6, 154.8, 146.0, 141.4, 138.7, 128.8, 122.2, 119.3, 118.2, 110.5, 105.2, 87.6, 69.4, 65.0, 61.1, 57.1, 55.0, 45.2, 33.4, 30.2, 29.2, 23.6, 19.7, 15.3, 5.7, 5.3, 2.6. HRMS (*m*/*z*) [M + H]^+^ calcd for C_27_H_32_N_2_O_5_, 465.2384; found, 465.2384, MMA = 0 ppm, mp 200 °C.

##### 17-Cyclopropylmethyl-3,14β-dihydroxy-4,5α-epoxy-6β-[3′-(furan-2″-yl)-*N*-methylpropanamido]morphinan Hydrochloride (**15**)

The title compound was prepared following general procedures 1, 2, 4, and 5 as a white solid in 23.4% yield. Hydrochloride salt: ^1^H NMR (400 MHz, DMSO-*d*_6_) δ 9.35 (d, *J* = 82.5 Hz, 1H, exchangeable), 8.78 (br s, 1H, exchangeable), 7.45 (d, *J* = 32.5 Hz, 1H), 6.76–6.62 (m, 2H), 6.34 (s, 1H, exchangeable), 6.28 (dd, *J* = 7.5, 5.5 Hz, 1H), 6.12–5.98 (m, 1H), 4.80 (dd, *J* = 31.1, 8.0 Hz, 1H), 4.10–4.03 (m, 0.3H), 3.83 (s, 1H), 3.54–3.46 (m, 0.7H), 3.42–3.34 (m, 1H), 3.04 (d, *J* = 12.0 Hz, 2H), 2.98 (s, 1H), 2.89 (s, 1H), 2.83 (s, 2H), 2.81–2.53 (m, 4H), 2.47–2.38 (m, 2H), 2.12–2.03 (m, 1H), 1.71 (d, *J* = 13.6 Hz, 1H), 1.42 (dt, *J* = 26.6, 13.3 Hz, 3H), 1.22 (d, *J* = 13.1 Hz, 1H), 1.10–1.13 (m, 1H), 0.72–0.66 (m, 1H), 0.59 (dd, *J* = 7.6, 3.9 Hz, 1H), 0.54–0.47 (m, 1H), 0.44–0.38 (m, 1H). ^13^C NMR (100 MHz, DMSO-*d*_6_) δ 171.0, 154.9, 141.6, 141.4, 141.1, 140.9, 129.9, 120.6, 119.8, 117.8, 110.3, 105.2, 104.8, 87.6, 69.6, 61.3, 57.0, 56.7, 46.4, 45.8, 30.0, 28.0, 23.2, 22.9, 21.7, 5.7, 5.1, 2.6. HRMS (*m*/*z*) [M + H]^+^ calcd for C_28_H_34_N_2_O_5_, 479.2540; found, 479.2528, MMA = 2.50 ppm, mp 237 °C.

##### 17-Cyclopropylmethyl-3,14β-dihydroxy-4,5α-epoxy-6β-[3′-(furan-2″-yl)propanamido]morphinan Hydrochloride (**16**)—Compound Previously Published in Ref (81)

The title compound was prepared following general procedures 3, 4, and 5 as a white solid in 51.7% yield. Hydrochloride salt: ^1^H NMR (400 MHz, DMSO-*d*_6_) δ 9.32 (s, 1H, exchangeable), 8.81 (br s, 1H, exchangeable), 8.19 (d, *J* = 7.8 Hz, 1H, exchangeable), 7.50 (s, 1H), 6.71 (d, *J* = 8.1 Hz, 1H), 6.63 (d, *J* = 8.1 Hz, 1H), 6.34 (d, *J* = 1.5 Hz, 1H), 6.12 (s, 1H, exchangeable), 6.09 (d, *J* = 2.1 Hz, 1H), 4.54 (d, *J* = 7.8 Hz, 1H), 3.82 (d, *J* = 5.0 Hz, 1H), 3.42–3.38 (m, 1H), 3.29 (s, 1H), 3.04 (dd, *J* = 22.5, 8.5 Hz, 2H), 2.82 (t, *J* = 7.1 Hz, 3H), 2.47–2.35 (m, 4H), 1.74–1.63 (m, 2H), 1.54–1.47 (m, 1H), 1.43 (d, *J* = 10.1 Hz, 1H), 1.33 (t, *J* = 13.2 Hz, 1H), 1.10–1.01 (m, 1H), 0.70–0.55 (m, 2H), 0.45 (ddd, *J* = 38.1, 9.0, 4.5 Hz, 2H). ^13^C NMR (100 MHz, DMSO-*d*_6_) δ 154.7, 142.1, 141.4, 141.2, 129.7, 120.7, 119.4, 117.9, 110.5, 105.2, 89.9, 69.7, 61.7, 56.7, 55.0, 50.6, 48.6, 46.5, 45.6, 33.8, 29.3, 27.4, 23.5, 23.0, 5.8, 5.2, 2.7. HRMS (*m*/*z*) [M + H]^+^ calcd for C_27_H_32_N_2_O_5_, 465.2384; found, 465.2367, MMA = 3.65 ppm, mp 249 °C.

##### 17-Cyclopropylmethyl-4,5α-epoxy-6α-[(2*E*)-3′-(furan-2″-yl)*N*-methylprop-2-enamido]-14β-hydroxymorphinan Hydrochloride (**17**)

The title compound was prepared following general procedures 1, 2, and 5 as a white solid in 27.3% yield. Hydrochloride salt: ^1^H NMR (400 MHz, DMSO-*d*_6_) δ 8.88 (br s, 1H, exchangeable), 7.81 (s, 1H), 7.37 (d, *J* = 15.2 Hz, 1H), 7.20 (t, *J* = 7.7 Hz, 1H), 6.89 (dd, *J* = 9.0, 6.2 Hz, 2H), 6.78 (d, *J* = 7.6 Hz, 1H), 6.70 (d, *J* = 7.8 Hz, 1H), 6.63 (s, 1H, exchangeable), 6.45 (d, *J* = 95.2 Hz, 1H), 5.09 (d, *J* = 14.1 Hz, 0.7H), 4.78 (s, 0.3H), 4.72 (d, *J* = 2.1 Hz, 1H), 3.96 (d, *J* = 5.6 Hz, 1H), 3.50–3.45 (m, 1H), 3.28–3.13 (m, 2H), 3.05 (d, *J* = 11.0 Hz, 1H), 3.00 (s, 2H), 2.98–2.92 (m, 1H), 2.86 (s, 1H), 2.70 (d, *J* = 12.7 Hz, 1H), 2.02–1.92 (m, 1H), 1.68–1.05 (m, 6H), 0.74–0.59 (m, 2H), 0.52–0.38 (m, 2H). ^13^C NMR (100 MHz, DMSO-*d*_6_) δ 165.6, 159.2, 151.2, 145.0, 132.7, 130.0, 128.9, 127.5, 118.7, 115.7, 114.2, 112.6, 105.9, 89.2, 68.9, 61.0, 57.0, 49.0, 45.0, 31.5, 29.9, 29.8, 24.1, 17.6, 5.7, 5.2, 2.6, 0.1. HRMS (*m*/*z*) [M + H]^+^ calcd for C_28_H_35_N_2_O_4_, 461.2435; found, 461.2413, MMA = 4.77 ppm, mp 269 °C.

##### 17-Cyclopropylmethyl-4,5α-epoxy-6α-[(2*E*)-3′-(furan-2″-yl)prop-2-enamido]-14β-hydroxymorphinan Hydrochloride (**18**)

The title compound was prepared following general procedures 3 and 5 as a light-yellow solid in 58.2% yield. Hydrochloride salt: ^1^H NMR (400 MHz, DMSO-*d*_6_) δ 8.89 (br s, 1H, exchangeable), 8.11 (d, *J* = 8.1 Hz, 1H, exchangeable), 7.78 (s, 1H), 7.25 (d, *J* = 15.6 Hz, 1H), 7.18 (t, *J* = 7.8 Hz, 1H), 6.78–6.74 (m, 2H), 6.68 (d, *J* = 7.9 Hz, 1H), 6.62–6.55 (m, 2H), 6.31 (br s, 1H, exchangeable), 4.68 (d, *J* = 3.7 Hz, 1H), 4.58–4.47 (m, 1H), 3.93 (d, *J* = 6.7 Hz, 1H), 3.49–3.44 (m, 2H), 3.21–3.14 (m, 1H), 3.06 (d, *J* = 11.9 Hz, 1H), 2.99–2.94 (m, 1H), 2.71 (dd, *J* = 22.8, 9.9 Hz, 1H), 2.45 (d, *J* = 4.8 Hz, 1H), 1.94–1.84 (m, 1H), 1.63 (d, *J* = 11.1 Hz, 1H), 1.50–1.38 (m, 2H), 1.09 (s, 1H), 0.98–0.89 (m, 1H), 0.66 (ddd, *J* = 17.2, 8.2, 4.5 Hz, 2H), 0.52–0.39 (m, 2H). ^13^C NMR (100 MHz, DMSO-*d*_6_) δ 164.3, 159.3, 151.0, 144.8, 132.7, 129.8, 127.6, 126.2, 119.5, 118.5, 113.7, 112.4, 106.2, 87.5, 69.3, 64.9, 57.0, 45.1, 45.0, 44.4, 30.1, 29.1, 24.2, 19.5, 5.7, 5.2, 2.6. HRMS (*m*/*z*) [M + H]^+^ calcd for C_27_H_30_N_2_O_4_, 447.2278; found, 447.2258, MMA = 4.47 ppm, mp 227 °C.

##### 17-Cyclopropylmethyl-4,5α-epoxy-6β-[(2*E*)-3′-(furan-2″-yl)-*N*-methylprop-2-enamido]-14β-hydroxymorphinan Hydrochloride (**19**)

The title compound was prepared following general procedures 1, 2, and 5 as a white solid in 42% yield. Hydrochloride salt: ^1^H NMR (400 MHz, DMSO-*d*_6_) δ 8.89 (s, 1H, exchangeable), 8.11 (d, *J* = 8.0 Hz, 1H), 7.78 (s, 1H), 7.25 (d, *J* = 15.6 Hz, 1H), 7.18 (t, *J* = 7.7 Hz, 1H), 6.82–6.72 (m, 2H), 6.68 (d, *J* = 7.9 Hz, 1H), 6.62–6.53 (m, 2H), 6.32 (s, 1H, exchangeable), 4.68 (d, *J* = 3.7 Hz, 1H), 4.52 (td, *J* = 8.6, 4.1 Hz, 1H), 3.94 (d, *J* = 6.6 Hz, 1H), 3.49 (s, 3H), 3.32–3.23 (m, 1H), 3.21–2.89 (m, 5H), 2.78–2.65 (m, 1H), 1.94–1.82 (m, 1H), 1.63 (d, *J* = 12.1 Hz, 1H), 1.49–1.37 (m, 2H), 1.02–0.85 (m, 1H), 0.72–0.60 (m, 2H), 0.52–0.32 (m, 2H). ^13^C NMR (100 MHz, DMSO-*d*_6_) δ 164.3, 159.3, 151.0, 144.8, 132.7, 129.8, 127.6, 126.2, 119.5, 118.5, 113.7, 112.4, 106.2, 87.5, 69.3, 64.9, 60.9, 57.0, 45.1, 44.4, 30.1, 29.1, 24.2, 19.5, 15.1, 5.7, 5.2, 2.6. HRMS (*m*/*z*) [M + H]^+^ calcd for C_28_H_32_N_2_O_4_, 461.2434; found, 461.2449, MMA = 3.04 ppm, mp 233 °C.

##### 17-Cyclopropylmethyl-4,5α-epoxy-6β-[(2*E*)-3′-(furan-2″-yl)prop-2-enamido]-14β-hydroxymorphinan Hydrochloride (**20**)

The title compound was prepared following general procedures 3 and 5 as a white solid in 26.4% yield. Hydrochloride salt: ^1^H NMR (400 MHz, DMSO-*d*_6_) δ 8.88 (br s, 1H, exchangeable), 8.45 (d, *J* = 8.2 Hz, 1H, exchangeable), 7.78 (s, 1H), 7.25–7.14 (m, 2H), 6.85 (d, *J* = 7.7 Hz, 1H), 6.77 (t, *J* = 6.1 Hz, 2H), 6.59 (d, *J* = 1.9 Hz, 1H), 6.39 (d, *J* = 15.6 Hz, 1H), 6.24 (br s, 1H, exchangeable), 4.58 (d, *J* = 7.9 Hz, 1H), 3.89 (d, *J* = 4.5 Hz, 1H), 3.58–3.45 (m, 2H), 3.19 (dd, *J* = 20.1, 5.5 Hz, 1H), 3.05 (d, *J* = 6.9 Hz, 1H), 2.90–2.83 (m, 1H), 2.44 (d, *J* = 8.4 Hz, 2H), 1.77 (t, *J* = 13.8 Hz, 2H), 1.59–1.52 (m, 1H), 1.45 (d, *J* = 8.8 Hz, 1H), 1.42–1.20 (m, 2H), 1.09 (t, *J* = 7.0 Hz, 1H), 0.72–0.65 (m, 1H), 0.60 (dd, *J* = 8.0, 4.4 Hz, 1H), 0.51 (dd, *J* = 9.3, 4.6 Hz, 1H), 0.46–0.39 (m, 1H). ^13^C NMR (100 MHz, DMSO-*d*_6_) δ 164.4, 155.8, 150.8, 144.8, 131.2, 129.5, 128.3, 126.4, 119.2, 113.9, 112.4, 109.0, 90.3, 69.6, 64.9, 61.5, 56.7, 50.6, 46.2, 44.9, 29.4, 27.2, 23.7, 15.1, 5.7, 5.1, 2.6. HRMS (*m*/*z*) [M + H]^+^ calcd for C_27_H_31_N_2_O_4_, 448.2357; found, 447.2268, MMA = 2.24 ppm, mp 196 °C.

##### 17-Cyclopropylmethyl-3,14β-dihydroxy-4,5α-epoxy-6α-[(2*E*)-3′-(furan-2″-yl)-*N*-methylprop-2-enamido]morphinan Hydrochloride (**21**)

The title compound was prepared following general procedures 1, 2, 4, and 5 as a white solid in 65.5% yield. Hydrochloride salt: ^1^H NMR (400 MHz, DMSO-*d*_6_) δ 9.32 (s, 1H, exchangeable), 8.84 (br s, 1H, exchangeable), 7.81 (s, 1H), 7.37 (d, *J* = 15.3 Hz,1H), 6.95–6.84 (m, 2H), 6.73 (d, *J* = 8.1 Hz, 1H), 6.66–6.57 (m, 2H), 6.42 (d, *J* = 101.5 Hz, 1H, exchangeable), 5.05 (d, *J* = 13.9 Hz, 1H), 4.71 (d, *J* = 3.3 Hz, 1H), 3.93 (d, *J* = 6.3 Hz, 1H), 3.40–3.34 (m, 1H), 3.26 (dd, *J* = 10.8, 4.2 Hz, 1H), 3.17–3.08 (m, 1H), 3.04 (s, 3H), 2.92 (d, *J* = 14.0 Hz, 2H), 2.69 (d, *J* = 12.8 Hz, 1H), 2.45 (d, *J* = 12.9 Hz, 1H), 2.01–1.90 (m, 1H), 1.66–1.53 (m, 2H), 1.41 (t, *J* = 10.3 Hz, 1H), 1.23–1.15 (m, 1H), 1.10–1.02 (m, 1H), 0.73–0.66 (m, 1H), 0.62 (dd, *J* = 8.3, 4.4 Hz, 1H), 0.53–0.45 (m, 1H), 0.43–0.35 (m, 1H). ^13^C NMR (100 MHz, DMSO-*d*_6_) δ 165.6, 151.2, 145.7, 145.0, 139.0, 128.9, 122.1, 119.4, 118.1, 115.8, 114.2, 112.6, 89.0, 69.0, 61.1, 57.0, 49.1, 45.7, 45.2, 31.6, 30.1, 29.8, 23.4, 17.9, 15.0, 5.7, 5.2, 2.6. HRMS (*m*/*z*) [M + H]^+^ calcd for C_28_H_32_N_2_O_5_, 477.2384; found, 477.2361, MMA = 4.82 ppm, mp 300 °C.

##### 17-Cyclopropylmethyl-3,14β-dihydroxy-4,5α-epoxy-6α-[(2*E*)-3′-(furan-2″-yl)prop-2-enamido]morphinan Hydrochloride (**22**)

The title compound was prepared following general procedures 3, 4, and 5 as a white solid in 81.6% yield. Hydrochloride salt: ^1^H NMR (400 MHz, DMSO-*d*_6_) δ 9.21 (s, 1H, exchangeable), 8.83 (br s, 1H, exchangeable), 8.05 (d, *J* = 7.9 Hz, 1H, exchangeable), 7.78 (s, 1H), 7.25 (d, *J* = 15.6 Hz, 1H), 6.78 (d, *J* = 3.3 Hz, 1H), 6.72 (d, *J* = 8.1 Hz, 1H), 6.62–6.55 (m, 3H), 6.26 (s, 1H, exchangeable), 4.67 (d, *J* = 3.8 Hz, 1H), 4.53–4.46 (m, 1H), 3.89 (d, *J* = 6.7 Hz, 1H), 3.26 (d, *J* = 11.6 Hz, 1H), 3.11–2.88 (m, 4H), 2.72 (d, *J* = 12.2 Hz, 1H), 2.45 (dd, *J* = 13.4, 5.0 Hz, 1H), 1.92–1.84 (m, 1H), 1.63 (d, *J* = 11.3 Hz, 1H), 1.43 (dd, *J* = 15.4, 9.3 Hz, 2H), 1.08–0.95 (m, 2H), 0.71–0.59 (m, 2H), 0.50–0.38 (m, 2H). ^13^C NMR (100 MHz, DMSO-*d*_6_) δ 164.3, 151.0, 146.0, 144.8, 138.8, 128.7, 126.2, 122.1, 119.5, 119.1, 118.2, 113.7, 112.4, 87.3, 69.3, 61.1, 57.0, 45.3, 45.2, 30.2, 29.2, 23.5, 23.4, 19.6, 5.7, 5.2, 2.5. HRMS (*m*/*z*) [M + H]^+^ calcd for C_27_H_30_N_2_O_5_ 463.2227; found, 463.2245, MMA = 3.89 ppm, mp 255 °C.

##### 17-Cyclopropylmethyl-3,14β-dihydroxy-4,5α-epoxy-6β-[(2*E*)-3′-(furan-2″-yl)-*N*-methylprop-2-enamido]morphinan Hydrochloride (**23**)—Compound Previously Published in Ref (80)

The title compound was prepared following general procedures 1, 2, 4, and 5 as a white solid in 39.8% yield. Hydrochloride salt: ^1^H NMR (400 MHz, DMSO-*d*_6_) δ 9.34 (d, *J* = 55.6 Hz, 1H, exchangeable), 8.79 (br s, 1H, exchangeable), 7.74 (d, *J* = 49.8 Hz, 1H), 7.22 (d, *J* = 39.6 Hz, 1H), 6.88–6.43 (m, 5H), 6.34 (d, *J* = 29.0 Hz, 1H, exchangeable), 4.86 (dd, *J* = 28.9, 8.1 Hz, 1H), 4.19 (s, 0.5H), 3.84 (s, 1H), 3.62 (s, 1H), 3.36 (s, 1H) 3.27 (d, *J* = 2.5 Hz, 1H), 3.15 (s, 1.5H), 3.07 (dd, *J* = 19.5, 5.1 Hz, 2H), 2.93 (s, 1.5H), 2.88 (d, *J* = 7.7 Hz, 1H), 2.45 (s, 1H), 2.12 (dd, *J* = 23.5, 10.6 Hz, 1H), 1.73 (d, *J* = 11.9 Hz, 1H), 1.51–1.00 (m, 5H), 0.72–0.56 (m, 2H), 0.53–0.39 (m, 2H). ^13^C NMR (100 MHz, DMSO-*d*_6_) δ 166.3, 165.5, 151.2, 144.9, 144.5, 141.8, 129.8, 128.8, 128.0, 120.4, 119.8, 117.8, 116.7, 114.1, 112.6, 69.6, 61.3, 56.6, 46.5, 45.9, 30.4, 27.1, 22.9, 22.0, 20.7, 5.7, 5.1, 2.6. HRMS (*m*/*z*) [M + H]^+^ calcd for C_28_H_32_N_2_O_5_, 477.2384; found, 477.2377, MMA = 1.47 ppm, mp 266 °C.

##### 17-Cyclopropylmethyl-3,14β-dihydroxy-4,5α-epoxy-6β-[(2*E*)-3′-(furan-2″-yl)prop-2-enamido]morphinan Hydrochloride (**24**)

The title compound was prepared following general procedures 3, 4, and 5 as a white solid in 39.6% yield. Hydrochloride salt: ^1^H NMR (400 MHz, DMSO-*d*_6_) δ 9.33 (s, 1H, exchangeable), 8.82 (br s, 1H, exchangeable), 8.45 (d, *J* = 7.8 Hz, 1H, exchangeable), 7.78 (s, 1H), 7.22 (d, *J* = 15.5 Hz, 1H), 6.78 (s, 1H), 6.72 (d, *J* = 8.1 Hz, 1H), 6.65 (d, *J* = 8.1 Hz, 1H), 6.59 (s, 1H), 6.40 (d, *J* = 15.6 Hz, 1H), 6.17 (s, 1H, exchangeable), 4.58 (d, *J* = 7.8 Hz, 1H), 3.84 (d, *J* = 4.3 Hz, 1H), 3.52 (d, *J* = 6.5 Hz, 1H), 3.06 (dd, *J* = 24.9, 6.2 Hz, 3H), 2.82 (dd, *J* = 36.0, 27.7 Hz, 2H), 2.46–2.36 (m, 2H), 1.80–1.67 (m, 2H), 1.59 (d, *J* = 10.3 Hz, 1H), 1.48–1.34 (m, 2H), 1.11–1.05 (m, 1H), 0.67 (s, 1H), 0.59 (s, 1H), 0.50 (s, 1H), 0.41 (s, 1H). ^13^C NMR (100 MHz, DMSO-*d*_6_) δ 164.4, 150.9, 144.8, 142.0, 141.3, 129.6, 126.3, 120.6, 119.3, 119.3, 117.9, 113.9, 112.4, 89.9, 69.7, 61.7, 56.7, 50.8, 46.5, 45.6, 29.4, 27.3, 23.7, 23.0, 5.7, 5.1, 2.6. HRMS (*m*/*z*) [M + H]^+^ calcd for C_27_H_30_N_2_O_5_, 463.2227; found, 463.2218, MMA = 1.94 ppm, mp 293 °C.

### Biological Methods

#### Cell Lines

Monoclonal mouse opioid receptor (denoted “m”, mKOR and mMOR) and monoclonal human opioid receptor (denoted “h”, hDOR) stably expressed in CHO cell lines were used. Cell lines heterologously expressed each of the cloned receptors to provide a reliable source of one opioid receptor subtype to be studied. This method also yields a higher receptor density, which provides for optimal signal-to-noise ratios in assays.^99^ Cell membrane homogenate prepared from these cell lines was used in *in vitro* assays.

#### Radioligand Binding Assay

Competitive radioligand binding assays were employed to assess the binding affinity and selectivity of designed compounds (**1**–**24**) for the KOR, MOR, and DOR. [^3^H]naloxone ([^3^H]NLX) was used to label the MOR, and [^3^H]diprenorphine ([^3^H]DPN) was used to label the KOR and DOR. A saturation assay was previously conducted to determine the K_D_ and *B*_max_ values for [^3^H]NLX at MOR and [^3^H]DPN at KOR and DOR. A fixed concentration of membrane protein (10–25 μg) was then incubated with the corresponding radioligand in the presence of varying concentrations of designed compound in TME buffer (50 mM Tris, 3 mM MgCl_2_, and 0.2 mM EGTA, pH 7.4) for 1.5 h at 30 °C. The bound radioligand was then separated by filtration using a Brandel harvester to determine total binding. Nonspecific binding was determined by adding an excess of unlabeled competitive ligand: 5 μM U50,488, naltrexone, and SNC80 for the KOR, MOR, and DOR, respectively. Specific (*i.e.*, opioid receptor-related) binding was defined as the difference between total binding and nonspecific binding. Data from these assays allowed determination of IC_50_ and Hill Slope. The Cheng-Prushoff equation () was then used to calculate the equilibrium dissociation constant of each compound (*i.e.*, *K*_*i*_).^125^ All assays were conducted in duplicate and repeated at least three times. Results were reported as mean ± SEM.

#### [^35^S]-GTPγS Functional Assay

[^35^S]-GTPγS binding assays were used to determine the functional activity of compounds for the KOR, MOR, and DOR (*i.e.*, agonist, partial agonist, antagonist, or inverse agonist). This was done by defining a compound’s efficacy in relation to that of the full agonist control for that receptor; that is U50,488, DAMGO, and DPDPE for the KOR, MOR and DOR, respectively. Membrane proteins (9–12 μg of mKOR-CHO, mMOR-CHO, and hDOR-CHO, in turn) were incubated with GDP (20 μM), [^35^S]-GTPγS (0.1 nM), varying concentrations of designed compound, 100 mM NaCl, and TME assay buffer for 1.5 h at 30 °C. Twenty μM of unlabeled GTPγS was used to determine nonspecific binding. To define the maximum effect for each receptor, 3, 3, and 5 μM, respectively, of U50,488, DAMGO, and SNC80 were used as maximally effective concentrations for the KOR, MOR, and DOR, respectively. To separate the bound radioligand from free radioligand after incubation, filtration through a GF/B glass fiber filter paper was performed and the filtrate was rinsed three times with ice-cold wash buffer (50 mM Tris-HCl, pH 7.2) using a Brandel harvester. A scintillation counter was then used to determine results. Dose response curves developed from [^35^S]-GTPγS data conveyed EC_50_ and *E*_max_ values. *E*_max_ values were related to their respective full agonists by (net-stimulated binding by ligand/net-stimulated by maximally effective concentration of full agonist) × 100%. All assays were conducted in duplicate and repeated at least three times. Results were reported as mean ± SEM.

### *In Vivo* Model Methods

#### Subjects and Approvals

All animals were acquired from Envigo (Frederick, MD, USA). Rats were individually housed, while mice were group housed (5 per cage) and both were maintained in temperature- and humidity-controlled vivaria programmed on 12 h light/dark cycles (lights on 6:00 AM to 6:00 PM). Food (Teklad Rat Diet, Envigo) and water were provided *ad libitum* in the home cage. Animal research protocols, maintenance, and enrichment were approved by the Virginia Commonwealth University Institutional Animal Care and Use Committee and in accordance with the “Guide for the Care and Use of Laboratory Animals”, eighth Ed. (2011). Mice were 6–8 week-old male Swiss Webster mice (*n* = 6/group). Rats were approximately 11–17 week-old male (*n* = 9) and female (*n* = 9) Sprague–Dawley rats.

#### Drugs

##### Studies in Mice

(-)-Morphine sulfate pentahydrate was purchased from Mallinckrodt (St. Louis, MO) and provided by the National Institute on Drug Abuse Drug Supply Program (Bethesda, MD). Naloxone HCl was purchased from Sigma-Aldrich (St. Louis, MO), norbinaltorphimine di-HCl and naltrindole HCl were purchased from MedChemExpress (Monmouth Junction, NJ), and β-funaltrexamine HCl was purchased from AABlocks (San Diego, CA). All test compounds synthesized herein were formulated as hydrochloride salts. All known and test compounds were dissolved in double-distilled water and used directly. All drug doses were expressed based on the salt forms listed above and delivered based on individual weights as collected immediately prior to each administration.

##### Studies in Rats

Fentanyl HCl was provided by the National Institute on Drug Abuse Drug Supply Program (Bethesda, MD) and dissolved in sterile saline. NLF HCl, compound **21**, and compound **23** synthesized herein were dissolved in sterile saline. All solutions were passed through a 0.22 μm sterile filter (Millex GV, Millipore Sigma, Burlington, MA) before intravenous (IV) administration. All drug doses were expressed based on the salt forms listed above and delivered based on individual weights as collected weekly.

#### WWTI Assay

##### General Procedure

WWTI assays were conducted in male Swiss Webster mice. A warm water bath was set to 56 ± 0.1 °C. Baseline tail withdrawal latencies (prior to compound administration) were measured, and a 2–4 s window for this baseline latency was used as exclusion criteria. To prevent tissue damage, a 10 s cutoff was established as the maximum amount of time a mouse’s tail may remain in the warm–water. In studies of agonism, test compounds were administered subcutaneously (s.c.), and withdrawal latencies were measured 20 min postinjection. In studies of antagonism, test compounds were administered s.c. Five min before s.c. morphine administration and withdrawal latencies were measured 20 min post morphine injection.

##### Data Analysis

The primary dependent measure was the antinociceptive response calculated as the percentage of the MPE (% MPE), wherein the MPE is a tail remaining in the warm–water bath for 10 s and % MPE = [(test – baseline latency)/(10 – baseline latency)] × 100. % MPE was calculated for each mouse. Data are shown as mean ± SEM. ED_50_ values were calculated using a least-squares linear regression analysis, followed by the calculation of 95% confidence intervals by the Bliss method. Data were compared using one-way ANOVA as appropriate and a significant ANOVA was followed by a Dunnet’s post hoc test as appropriate. Statistical significance was defined as *p* < 0.05.

##### Receptor Selectivity Study

Baseline tail withdrawal latencies were measured according to the “general” procedure prior to any compound administration. Mice in nor-BNI groups received 10 mg/kg nor-BNI s.c. Twenty-four h prior to 0.1 mg/kg test compound s.c. injection. Mice in β-FNA groups received 10 mg/kg β-FNA s.c. Twenty-four h prior to 0.1 mg/kg test compound s.c. injection. Mice in NTI groups received 15 mg/kg NTI s.c. Thirty min prior to 0.1 mg/kg test compound s.c. injection. Mice in the nor-BNI + NTI groups received both nor-BNI and NTI according to the same dosing schedule as mice receiving a single antagonist compound. Withdrawal latencies for mice in all groups were acquired 20 min post-test-compound administration.^28,29^

##### Time-Course Study

Baseline tail withdrawal latencies were measured according to the “general” procedure 1 h prior to s.c. administration of test compound. Immediately following injection, withdrawal latencies were remeasured (time point 0). Withdrawal latencies were acquired after the first 30 min and after every h until test compound effects wore off with a maximum of 10 h post injection.

#### Locomotor Activity

##### Apparatus

Six open field activity chambers (Med Associates, St. Albans, VT) were used in the locomotor activity study. Each chamber is located inside a sound-attenuating cubicle (Med Associates) and is equipped with a ventilation system. The interior of the chamber consists of a 27 × 27 cm plexiglass enclosure wired with photobeam cells and connected to a computer console that monitors the activity of the animal.

##### Methods

The day before the experiment, mice were acclimated to the activity chambers for a period of 30 min. On the day of the experiment, mice were administered vehicle or test compound s.c., placed into the activity chambers immediately and activity was monitored and recorded for a period of 30 min.

##### Data Analysis

The primary dependent measures are total (1) distance traveled (cm), (2) ambulatory counts, (3) vertical counts, and (4) average speed (cm/s) within the 30 min session. Data are shown as mean ± SEM. Data were compared using one-way or two-way ANOVA as appropriate and a significant ANOVA was followed by a Dunnet’s post hoc test as appropriate. Statistical significance was defined as *p* < 0.05.

#### Abuse Liability *via* a Self-Administration Model

##### Apparatus and Catheter Maintenance

Twelve modular operant chambers located within sound-attenuating cubicles were assembled as previously reported.^117^ Following each behavioral session, intravenous (IV) catheters were flushed with 0.1 mL of gentamicin (4 mg/mL) and 0.1 mL of heparinized saline (30 units/mL). Catheter patency was verified at least every 2 weeks and at the conclusion of the study *via* instantaneous muscle tone loss precipitated by IV methohexital (0.5 mg) administration.

##### Training

Post surgical implantation of vascular access ports as previously published, rats were trained to self-administer 3.2 μg/kg/inf IV fentanyl using the following steps.^117^ First, daily 2 h behavioral sessions occurring Mon-Sun (approximately 9:30–11:30 AM) were used to train rats to respond for IV drug infusions (inf) under a fixed-ratio (FR) 1/20 s time out schedule of reinforcement. The “drug” was a 3.2 μg/kg/inf unit dose of fentanyl. This schedule of reinforcement was in effect until the number of earned infusions in a single session was approximately 20. Then, the FR requirement was increased to FR5 and remained in effect until the number of earned infusions was within 20% of the running mean with no upward or downward trends for three consecutive days.

Once drug self-administration training was complete, the drug syringe attached to the syringe pump was intermittently replaced with a saline syringe. This occurred on a double alternation schedule (*i.e.*, DDSSDDSS, wherein D = drug and S = saline) until the number of infusions earned on the first “saline day” after a “drug day” was ≥75% below the number of infusions earned on the “drug day” preceding it. Upon meeting this criterion, rats were switched to a single alternation schedule (*i.e.*, DSDS) until the number of saline infusions earned was ≥75% below the number of infusions earned on the drug day preceding it for two consecutive alternations. The same program was used to run saline sessions, and saline infusion duration was equivalent to a “drug” (3.2 μg/kg/inf) day.

##### Methods

Once all training criteria were met, test sessions were inserted into the sequence (*i.e.*, DSTSDTDST, wherein T = test). As a positive control, the fentanyl dose–effect curve (0.32, 1, 3.2, 10 μg/kg/inf) was first established using this schedule. NLF (0.1, 0.32, 1, 3.2, 10 μg/kg/inf), compound **23** (0.32, 1, 3.2, 10 μg/kg/inf) [100 μg/kg/inf, *n* = 1], and compound **21** (0.32, 1, 3.2, 10 μg/kg/inf) were then assessed using the same procedure. Each dose of fentanyl (other than the 3.2 μg/kg/inf test dose), NLF, compound **23**, and compound **21** was given once in each rat.

##### Data Analysis

The primary dependent measure was the number of infusions earned per session. Data were compared using one-way repeated measures ANOVA, and the Geisser-Greenhouse correction was applied as appropriate (Prism 9, GraphPad, La Jolla, CA, USA). A significant ANOVA was followed by a Dunnet’s post hoc test as appropriate. Statistical significance was defined as *p* < 0.05.

### ADMET Screening Methods

#### Drug-Distribution Study

The test compound was administered s.c., and a group of Swiss Webster mice was euthanized by decapitation at each time point (5, 10, 30, 60 min post injection) allowing whole brain and blood samples to be harvested. Brain samples were washed with saline to ensure removal of any blood on the isolated brain and were then immersed in 300 μL saline. Blood samples were centrifuged for 10 min (15,000*g*; 4 °C) to collect plasma. Brain and plasma samples were stored at −80 °C until further analysis.

##### LC/MS Analysis

Identification and quantification of test compound in mouse brain and plasma were performed using a modification of a previously described method with an internal standard of naloxone-*d*_5_.^126^ Compounds were extracted from both blood and brain by a liquid/liquid extraction. Briefly, brain tissue samples were homogenized 1-part tissue to 3-parts water. Seven-point calibration curves (10–1000 ng/mL or ng/g) in plasma, drug free control, a negative control without internal standard in plasma and brain, and quality control specimens in plasma and brain (30, 300, and 750 ng/mL or ng/g) were prepared and analyzed with each batch of samples. Naltrexone-*d*_5_, was added at 10 ng/mL concentration to aliquots of either 100 μL for blood or 400 μL for brain homogenate to each calibrator, control, or specimen except the negative control. To these samples, 0.5 mL of saturated carbonate/bicarbonate buffer (1:1, pH 9.5) and 2.0 mL of chloroform/2-propanol (8:2) were added. The samples were then mixed and centrifuged. The top, aqueous layer was aspirated, and the organic layer was transferred to a clean test tube and evaporated to dryness under nitrogen. The samples were then reconstituted in 80:20 water/water and transferred to autosampler vials for analysis. The chromatographic separation of test compounds and naloxone-*d*_5_ was accomplished using a Shimadzu Nexera X2 liquid chromatography system and a Zorbax XDB-C18 4.6 × 75 mm, 3.5 μm column (Agilent Technologies, Santa Clara, CA). The mobile phases consisted of (A) water with 1 g/L ammonium formate and 0.1% formic acid, and (B) methanol. The flow rate was set to 1 mL/min. The mobile phase started with 20% B and was increased to 80% B at 1.0 min and held constant for 1.5 min before returning to 20% B. The system detector was a Sciex 6500 QTRAP system with an IonDrive Turbo V source for TurboIonSpray (Sciex, Ontario, Canada) that had the curtain gas flow rate set at 30 mL/min and the ion source gases 1 and 2 at 60 mL/min. The source temperature was set at 650 °C with an ionspray voltage was 5500 V. The declustering potential was 58 eV.

##### Data Analysis

The primary dependent measures are (1) the plasma concentration of the test compound (μg/mL), (2) the brain concentration of the test compound (μg/g), and (3) the brain-to-plasma concentration ratio calculated as [brain]/[plasma]. Concentrations were determined by a linear regression plot based on peak ratios of the calibrators. Data are shown as mean ± SEM. Data were compared using one-way ANOVA as appropriate and a significant ANOVA was followed by a Dunnet’s post hoc test as appropriate. Statistical significance was defined as *p* < 0.05.

#### Metabolism Profile

Compounds **21** and **23** and controls (1 μM for all; except imipramine, propranolol, terfenadine: 0.1 μM) were incubated with human and rat liver S9 fractions (0.3 mg/mL) with the addition of 1 mM glutathione, uridine 5′-diphosphoglucuronic acid (UDPGA), and 3′-phosphoadenosine-5′-phosphosulfate (PAPS) cofactors at 37 °C following a previously published protocol.^127^ After 0, 15, 30, 45, and 60 min of incubation, the concentration of each compound was determined using HPLC-MS/MS. After the experiment, metabolic stability was expressed as the percentage of remaining parent compound as calculated by comparing the peak area of the compound at each time point relative to that at time point 0. Half-life (*T*_1/2_) was estimated using the slope of the initial linear range of the logarithmic curve of the remaining compound (%) as a function of time while assuming first-order kinetics. The apparent intrinsic clearance (CL_int_, μL/min/mg) was calculated according to 

#### Cardiac Toxicity

The cardiac toxicity assessment was performed using the IonChannelProfiler hERG Qube APC assay in CHO-hERG cells in the presence of 0.1% pluronic F-68 nonionic surfactant and at approximately 25 °C. First, whole cell configuration was achieved. Then, the cell was held at −80 mV. A 500 ms pulse to −40 mV was delivered to measure the leak current which was subtracted from the hERG online current. The cell was depolarized to +40 mV for 500 ms and then back to −80 mV over a 100 ms ramp to yield the hERG tail current. This sequence was repeated once every 8 s to monitor the current amplitude. The primary dependent measure was the maximum tail current evoked on stepping to +40 mV and ramping back to −80 mV. All data were filtered for seal quality, seal drop, and current amplitude. The peak current amplitude was calculated before and after compound addition, and the amount of block was assessed by dividing the test compound current amplitude by the baseline current amplitude. Baseline data is the mean hERG current amplitude collected 15 s at the end of the baseline period. Test compound data is the mean hERG current amplitude collected 15 s at the end of the test concentration application for each concentration. IC50 values were determined by a nonlinear, least-squares regression analysis.

## Supporting Information Available

Spectral data for target compounds (^1^H NMR, ^13^C NMR, and HPLC chromatograms) and *in silico* physiochemical property prediction of target compounds (PDF)
Molecular formula strings and *in vitro* data (CSV)

## Abbreviations

ACD: Advanced Chemistry Development
BBB: blood–brain barrier
CHO: Chinese hamster ovary
CL: confidence level
CNS: central nervous system
DAMGO: [d-Ala2-MePhe4-Gly(ol)5]ekephalin
DOR: delta opioid receptor
EDCI: 1-ethyl-3-(3-(dimethylamino)propyl)carbodiimide
GPCR: G protein-coupled receptor
hERG: human ether-à-go-go-related-gene
HOBt: hydroxybenzotriazole
KOR: kappa opioid receptor
MOR: mu opioid receptor
MPE %: percentage of the maximum possible effect
NIDA: National Institute on Drug Abuse
NLF: nalfurafine
NTI: naltrindole
NLX: naloxone
NOP: nociceptin opioid peptide receptor
nor-BNI: nor-binaltorphimine
SAR: structure–activity relationship
TPSA: topological polar surface area
WWTI: warm-water tail immersion
β-FNA: beta-funaltrexamine

## References

[ref1] Thomas D.; Wessel C. State of Innovation in Pain and Addiction, 2023. www.bio.org/iareports.

[ref2] Torvik K.; Kaasa S.; Kirkevold Ø.; Rustøen T. Pain in Patients Living in Norwegian Nursing Homes. Palliat. Med. 2009, 23 (1), 8–16. 10.1177/0269216308098800. 18952745

[ref3] Strohbuecker B.; Mayer H.; Evers G. C. M.; Sabatowski R. Pain Prevalence in Hospitalized Patients in a German University Teaching Hospital. J. Pain Symptom Manag. 2005, 29 (5), 498–506. 10.1016/j.jpainsymman.2004.08.012. 15904752

[ref4] Deandrea S.; Montanari M.; Moja L.; Apolone G. Prevalence of Undertreatment in Cancer Pain. A Review of Published Literature. Ann. Oncol. 2008, 19, 1985–1991. 10.1093/annonc/mdn419. 18632721 PMC2733110

[ref5] Majedi H.; Dehghani S. S.; Soleyman-Jahi S.; Tafakhori A.; Emami S. A.; Mireskandari M.; Hosseini S. M. Assessment of Factors Predicting Inadequate Pain Management in Chronic Pain Patients. Anesth. Pain Med. 2019, 9 (6), e97229 10.5812/aapm.97229. 32280619 PMC7118688

[ref6] Kenan K.; Mack K.; Paulozzi L. Trends in Prescriptions for Oxycodone and Other Commonly Used Opioids in the United States, 2000–2010. Open Med. 2012, 6 (2), e41. 23696768 PMC3659213

[ref7] Anighoro A.; Bajorath J.; Rastelli G. Polypharmacology: Challenges and Opportunities in Drug Discovery. J. Med. Chem. 2014, 57, 7874–7887. 10.1021/jm5006463. 24946140

[ref8] Roth B. L.; Sheffler D. J.; Kroeze W. K. Magic Shotguns versus Magic Bullets: Selectively Non-Selective Drugs for Mood Disorders and Schizophrenia. Nat. Rev. 2004, 3, 353–359. 10.1038/nrd1346. 15060530

[ref9] Aldrich J. V.; McLaughlin J. P. Peptide Kappa Opioid Receptor Ligands: Potential for Drug Development. AAPS J. 2009, 11, 312–322. 10.1208/s12248-009-9105-4. 19430912 PMC2691465

[ref10] Naloxone. Substance Abuse and Mental Health Services Administration 2021. Https://Www.Samhsa.Gov/Medication-Assisted-Treatment/Medications-Counseling-Related-Conditions/Naloxone (accessed September 28, 2021).

[ref11] Abraha I.; Cusi C. Oral Naltrexone Maintenance Treatment for Opioid Dependence1. Alcohol and Drug Misuse; John Wiley & Sons, Ltd, 2012; pp 118–120.

[ref12] Wang D.; Sun X.; Sadee W. Different Effects of Opioid Antagonists on μ-δ-and κ-Opioid Receptors with and without Agonist Pretreatment. J. Pharmacol. Exp. Ther. 2007, 321 (2), 544–552. 10.1124/jpet.106.118810. 17267582

[ref13] Robinson S. E. Buprenorphine: An Analgesic with an Expanding Role in the Treatment of Opioid Addiction. CNS Drug Rev. 2002, 8 (4), 377–390. 10.1111/j.1527-3458.2002.tb00235.x. 12481193 PMC6741692

[ref14] Paton K. F.; Atigari D. V.; Kaska S.; Prisinzano T.; Kivell B. M. Strategies for DevelopingκOpioid Receptor Agonists for the Treatment of Pain with Fewer Side Effects. J. Pharmacol. Exp. Ther. 2020, 375 (2), 332–348. 10.1124/jpet.120.000134. 32913006 PMC7589957

[ref15] Larsen D.; Christopher; Maani V. Nalbuphine Continuing Education Activity, 2023. https://www.ncbi.nlm.nih.gov/books/NBK534283/.

[ref16] Schiller P. W.; Fundytus M. E.; Merovitz L.; Weltrowska G.; Nguyen T. M. D.; Lemieux C.; Chung N. N.; Coderre T. J. The Opioid μ Agonist/δ Antagonist DIPP-NH 2 [Ψ] Produces a Potent Analgesic Effect, No Physical Dependence, and Less Tolerance than Morphine in Rats. J. Med. Chem. 1999, 42, 3520–3526. 10.1021/jm980724+. 10479285

[ref17] Ananthan S. Opioid ligands with mixed μ/δ opioid receptor interactions: An emerging approach to novel analgesics. AAPS J. 2006, 8, E118–E125. 10.1208/aapsj080114. 16584118 PMC2751430

[ref18] Bird M. F.; McDonald J.; Horley B.; O’Doherty J. P.; Fraser B.; Gibson C. L.; Guerrini R.; Caló G.; Lambert D. G. MOP and NOP Receptor Interaction: Studies with a Dual Expression System and Bivalent Peptide Ligands. PLoS One 2022, 17 (1), e0260880 10.1371/journal.pone.0260880. 35061679 PMC8782398

[ref19] Premium Insights Likelihood of Approval and Phase Transition Success Rate Model-Cebranopadol in Substance (Drug) Abuse Related Company Profiles Tris Pharma Inc View All, 2024. https://www.pharmaceutical-technology.com/data-insights/cebranopadol-tris-pharma-substance-drug-abuse-likelihood-of-approval/.

[ref20] Ziemichod W.; Kotlinska J.; Gibula-Tarlowska E.; Karkoszka N.; Kedzierska E. Cebranopadol as a Novel Promising Agent for the Treatment of Pain. Molecules 2022, 27, 3987 10.3390/molecules27133987. 35807228 PMC9268744

[ref21] Bonifazi A.; Battiti F. O.; Sanchez J.; Zaidi S. A.; Bow E.; Makarova M.; Cao J.; Shaik A. B.; Sulima A.; Rice K. C.; Katritch V.; Canals M.; Lane J. R.; Newman A. H. Novel Dual-Target μ-Opioid Receptor and Dopamine D3Receptor Ligands as Potential Nonaddictive Pharmacotherapeutics for Pain Management. J. Med. Chem. 2021, 64 (11), 7778–7808. 10.1021/acs.jmedchem.1c00611. 34011153 PMC9308496

[ref22] Bonifazi A.; Saab E.; Sanchez J.; Nazarova A. L.; Zaidi S. A.; Jahan K.; Katritch V.; Canals M.; Lane J. R.; Newman A. H. Pharmacological and Physicochemical Properties Optimization for Dual-Target Dopamine D3 (D3R) and μ-Opioid (MOR) Receptor Ligands as Potentially Safer Analgesics. J. Med. Chem. 2023, 66 (15), 10304–10341. 10.1021/acs.jmedchem.3c00417. 37467430 PMC11091828

[ref23] Rios M. New Insights into the Mechanisms Underlying the Effects of BDNF on Eating Behavior. Neuropsychopharmacology 2011, 36 (1), 368–369. 10.1038/npp.2010.139. 21116262 PMC3055507

[ref24] Vanderah T. W. Delta and Kappa Opioid Receptors as Suitable Drug Targets for Pain. Clin. J. Pain 2010, 26, S10–S15. 10.1097/ajp.0b013e3181c49e3a. 20026960

[ref25] Al-Hasani R.; Bruchas M. R. Molecular Mechanisms of Opioid Receptor-Dependent Signaling and Behavior. Anesthesiology 2011, 115, 1363–1381. 10.1097/aln.0b013e318238bba6. 22020140 PMC3698859

[ref26] Miaskowski C.; Taiwo Y. O.; Levine J. D. K-And B-Opioid Agonists Synergize to Produce Potent Analgesia, 1990; Vol. 509. 10.1016/0006-8993(90)90327-8 2155044

[ref27] Hartrick C. T.; Poulin D.; Molenaar R.; Hartrick A. Dual-Acting Peripherally Restricted Delta/Kappa Opioid (Cav1001) Produces Antinociception in Animal Models of Sub-Acute and Chronic Pain. J. Pain Res. 2020, 13, 2461–2474. 10.2147/JPR.S262303. 33116788 PMC7547792

[ref28] Li M.; Stevens D. L.; Arriaga M.; Townsend E. A.; Mendez R. E.; Blajkevch N. A.; Selley D. E.; Banks M. L.; Negus S. S.; Dewey W. L.; et al. Characterization of a Potential KOR/DOR Dual Agonist with No Apparent Abuse Liability via a Complementary Structure—Activity Relationship Study on Nalfurafine Analogues. ACS Chem. Neurosci. 2022, 13, 3608–3628. 10.1021/acschemneuro.2c00526. 36449691 PMC10243363

[ref29] Atigari D. V.; Paton K. F.; Uprety R.; Váradi A.; Alder A. F.; Scouller B.; Miller J. H.; Majumdar S.; Kivell B. M. The Mixed Kappa and Delta Opioid Receptor Agonist, MP1104, Attenuates Chemotherapy-Induced Neuropathic Pain. Neuropharmacology 2021, 185, 108445 10.1016/j.neuropharm.2020.108445. 33383089 PMC8344368

[ref30] Samoson A.; Tuherm T.; Past J.; Reinhold A.; Anupõld T.; Heinmaa I.; Meijere A.; Houk K. N.; Kessler H.; Lehn J. M.; Ley S. V.; Schreiber S. L.; Thiem J.; Trost B. M.; Vögtle F.; Yamamoto H.; Klinowski J. Topics in Current Chemistry; Springer, 2011; Vol. 299.

[ref31] Kozono H.; Yoshitani H.; Nakano R. Post-marketing surveillance study of the safety and efficacy of nalfurafine hydrochloride (Remitch capsules 2.5 μg) in 3,762 hemodialysis patients with intractable pruritus. Int. J. Nephrol. Renovasc. Dis. 2018, 11, 9–24. 10.2147/IJNRD.S145720. 29391822 PMC5774492

[ref32] Kumagai H.; Ebata T.; Takamori K.; Muramatsu T.; Nakamoto H.; Suzuki H. Effect of a Novel Kappa-Receptor Agonist, Nalfurafine Hydrochloride, on Severe Itch in 337 Haemodialysis Patients: A Phase III, Randomized, Double-Blind, Placebo-Controlled Study. Nephrol. Dial. Transplant. 2010, 25, 1251–1257. 10.1093/ndt/gfp588. 19926718

[ref33] Inui S. Nalfurafine Hydrochloride to Treat Pruritus: A Review. Clin. Cosmet. Invest. Dermatol. 2015, 8, 249–255. 10.2147/CCID.S55942. PMC4433050 26005355

[ref34] Nagase H.; Hayakawa J.; Kawamura K.; Kawai K.; Takezawa Y.; Matsuura H.; Tajima C.; Endo T. Discovery of a Structurally Novel Opioid .KAPPA.-Agonist Derived from 4,5-Epoxymorphinan. Chem. Pharm. Bull. (Tokyo) 1998, 46 (2), 366–369. 10.1248/cpb.46.366. 9501472

[ref35] Kumagai H.; Ebata T.; Takamori K.; Miyasato K.; Muramatsu T.; Nakamoto H.; Kurihara M.; Yanagita T.; Suzuki H. Efficacy and Safety of a Novel K-Agonist for Managing Intractable Pruritus in Dialysis Patients. Am. J. Nephrol. 2012, 36 (2), 175–183. 10.1159/000341268. 22868684

[ref36] TRK-820 Study in Subjects on Hemodialysis with or without Uremic Pruritus. Clinical Trials.Gov Identifier: NCT03002233. Updated July 21, 2017. Accessed May 6, 2024.

[ref37] Safety Study of TRK-820 for Patients with Hemodialysis. Clinicaltrials.Gov Identifier: NCT01248650. Updated November 25, 2010. Accessed May 6, 2024.

[ref38] A Multi-Site Bridging Study of Nalfurafine Hydrochloride Orally Disintegrating Tablet. Clinicaltrials.Gov Identifier: NCT04728984. Updated January 28, 2021. Accessed May 6, 2024.

[ref39] Efficacy and Safety of MT-9938 for Treatment of Uremic Pruritus in Subjects with End-Stage Renal Disease Receiving Hemodialysis. Clinicaltrials.Gov Identifier: NCT01660243. Updated February 9, 2022. Accessed May 6, 2024.

[ref40] Efficacy and Safety Study of TRK-820 to Treat Conventional-Treatment-Resistant Pruritus in Patients Receiving Hemodialysis. Clinicaltrials.Gov Identifier: NCT01513161. Updated January 20, 2012. Accessed May 6, 2024.

[ref41] A Randomized-Withdrawal Phase 3 Study Evaluating the Safety and Efficacy of Oral Nalfurafine HCl (AC-820) in Subjects on Hemodialysis with Uremic Pruritus (Renal Itch). Clinicaltrials.Gov Identifier: NCT00793156. Updated February 4, 2010. Accessed May 6, 2024.

[ref42] Nalfurafine Hydrochloride for Pruritus in Patients with Primary Biliary Cholangitis. Clinicaltrials.Gov Identifier: NCT02659696. Updated April 26, 2018. Accessed May 6, 2024.

[ref43] Phase II Study of TRK-820 Soft Capsules—Intractable Pruritus in Patients with Chronic Liver Disease. Clinicaltrials.Gov Identifier: NCT00638495. Updated February 1, 2010. Accessed May 6, 2024.

[ref44] Endoh T.; Matsuura H.; Tajima A.; Izumimoto N.; Tajima C.; Suzuki T.; Saitoh A.; Suzuki T.; Narita M.; Tseng L.; Nagase H. Potent antinociceptive effects of TRK-820, a novel κ-opioid receptor agonist. Life Sci. 1999, 65 (16), 1685–1694. 10.1016/S0024-3205(99)00417-8. 10573186

[ref45] Endoh T.; Tajima A.; Izumimoto N.; Suzuki T.; Saitoh A.; Suzuki T.; Narita M.; Kamei J.; Tseng L. F.; Mizoguchi H.; Nagase H. TRK-820, a Selective κ-Opioid Agonist, Produces Potent Antinociception in Cynomolgus Monkeys. Jpn. J. Pharmacol. 2001, 85, 282–290. 10.1254/jjp.85.282. 11325021

[ref46] Endoh T.; Tajima A.; Suzuki T.; Kamei J.; Suzuki T.; Narita M.; Tseng L.; Nagase H. Characterization of the Antinociceptive Effects of TRK-820 in the Rat. Eur. J. Pharmacol. 2000, 387, 133–140. 10.1016/S0014-2999(99)00815-8. 10650153

[ref47] Liu J. J.; Chiu Y.-T.; DiMattio K. M.; Chen C.; Huang P.; Gentile T. A.; Muschamp J. W.; Cowan A.; Mann M.; Liu-Chen L.-Y. Phosphoproteomic Approach for Agonist-Specific Signaling in Mouse Brains: MTOR Pathway Is Involved in κ Opioid Aversion. Neuropsychopharmacology 2019, 44 (5), 939–949. 10.1038/s41386-018-0155-0. 30082888 PMC6462019

[ref48] Townsend E. A.; Naylor J. E.; Negus S. S.; Edwards S. R.; Qureshi H. N.; McLendon H. W.; McCurdy C. R.; Kapanda C. N.; do Carmo J. M.; da Silva F. S.; Hall J. E.; Sufka K. J.; Freeman K. B. Effects of Nalfurafine on the Reinforcing, Thermal Antinociceptive, and Respiratory-Depressant Effects of Oxycodone: Modeling an Abuse-Deterrent Opioid Analgesic in Rats. Psychopharmacology (Berl) 2017, 234 (17), 2597–2605. 10.1007/s00213-017-4652-3. 28567699 PMC5709149

[ref49] Kaski S. W.; White A. N.; Gross J. D.; Trexler K. R.; Wix K.; Harland A. A.; Prisinzano T. E.; Aubé J.; Kinsey S. G.; Kenakin T.; Siderovski D. P.; Setola V. Preclinical Testing of Nalfurafine as an Opioid-sparing Adjuvant that Potentiates Analgesia by the Mu Opioid Receptor-targeting Agonist Morphine. J. Pharmacol. Exp. Therapeut. 2019, 371 (2), 487–499. 10.1124/jpet.118.255661. PMC6863463 31492823

[ref50] Tsuji M.; Takeda H.; Matsumiya T.; Nagase H.; Narita M.; Suzuki T. The Novel-Opioid Receptor Agonist TRK-820 Suppresses the Rewarding and Locomotor-Enhancing Effects of Morphine in Mice. Life Sci. 2001, 68, 1717–1725. 10.1016/S0024-3205(01)00957-2. 11270618

[ref51] Zhou Y.; Kreek M. J. Combination of Clinically Utilized Kappa-Opioid Receptor Agonist Nalfurafine With Low-Dose Naltrexone Reduces Excessive Alcohol Drinking in Male and Female Mice. Alcohol. Clin. Exp. Res. 2019, 43 (6), 1077–1090. 10.1111/acer.14033. 30908671 PMC6551307

[ref52] Mori T.; Nomura M.; Nagase H.; Narita M.; Suzuki T. Effects of a newly synthesized κ-opioid receptor agonist, TRK-820, on the discriminative stimulus and rewarding effects of cocaine in rats. Psychopharmacology (Berl) 2002, 161, 17–22. 10.1007/s00213-002-1028-z. 11967626

[ref53] Zamarripa C. A.; Patel T. R.; Williams B. C.; Pareek T.; Schrock H. M.; Prisinzano T. E.; Freeman K. B. The Kappa-Opioid Receptor Agonist, Nalfurafine, Blocks Acquisition of Oxycodone Self-Administration and Oxycodone’s Conditioned Rewarding Effects in Male Rats. Behav. Pharmacol. 2020, 31, 792–797. 10.1097/FBP.0000000000000581. 32804774 PMC7655691

[ref54] Togashi Y.; Umeuchi H.; Okano K.; Ando N.; Yoshizawa Y.; Honda T.; Kawamura K.; Endoh T.; Utsumi J.; Kamei J.; Tanaka T.; Nagase H. Antipruritic activity of the κ-opioid receptor agonist, TRK-820. Eur. J. Pharmacol. 2002, 435, 259–264. 10.1016/S0014-2999(01)01588-6. 11821035

[ref55] Inan S.; Lee D. Y.-W.; Liu-Chen L. Y.; Cowan A. Comparison of the Diuretic Effects of Chemically Diverse Kappa Opioid Agonists in Rats: Nalfurafine, U50,488H, and Salvinorin A. Naunyn Schmiedebergs Arch Pharmacol 2009, 379 (3), 263–270. 10.1007/s00210-008-0358-8. 18925386

[ref56] Schattauer S. S.; Kuhar J. R.; Song A.; Chavkin C. Nalfurafine Is a G-Protein Biased Agonist Having Significantly Greater Bias at the Human than Rodent Form of the Kappa Opioid Receptor. Cell Signal 2017, 32, 59–65. 10.1016/j.cellsig.2017.01.016. 28088389 PMC5779083

[ref57] Kenakin T.; Christopoulos A. Signalling Bias in New Drug Discovery: Detection, Quantification and Therapeutic Impact. Nat. Rev. Drug Discov. 2013, 12 (3), 205–216. 10.1038/nrd3954. 23411724

[ref58] Van Der Westhuizen E. T.; Breton B.; Christopoulos A.; Bouvier M. Quantification of Ligand Bias for Clinically Relevant β_2_-Adrenergic Receptor Ligands: Implications for Drug Taxonomy. Mol. Pharmacol. 2014, 85 (3), 492–509. 10.1124/mol.113.088880. 24366668

[ref59] Fujii H.; Imaide S.; Hirayama S.; Nemoto T.; Gouda H.; Hirono S.; Nagase H. Essential structure of opioid κ receptor agonist nalfurafine for binding to the κ receptor 3: Synthesis of decahydro(iminoethano)phenanthrene derivatives with an oxygen functionality at the 3-position and their pharmacologies. Bioorg. Med. Chem. Lett. 2012, 22, 7711–7714. 10.1016/j.bmcl.2012.09.101. 23103094

[ref60] Mores K. L.; Cummins B. R.; Cassell R. J.; van Rijn R. M. A Review of the Therapeutic Potential of Recently Developed G Protein-Biased Kappa Agonists. Front. Pharmacol 2019, 10, 407 10.3389/fphar.2019.00407. 31057409 PMC6478756

[ref61] Nagi K.; Pineyro G. Practical Guide for Calculating and Representing Biased Signaling by GPCR Ligands: A Stepwise Approach. Methods 2016, 92, 78–86. 10.1016/j.ymeth.2015.09.010. 26364590

[ref62] Rajagopal S.; Ahn S.; Rominger D. H.; Gowen-MacDonald W.; Lam C. M.; DeWire S. M.; Violin J. D.; Lefkowitz R. J. Quantifying Ligand Bias at Seven-Transmembrane Receptors. Mol. Pharmacol. 2011, 80 (3), 367–377. 10.1124/mol.111.072801. 21610196 PMC3164332

[ref63] Lovell K. M.; Frankowski K. J.; Stahl E. L.; Slauson S. R.; Yoo E.; Prisinzano T. E.; Aube J.; Bohn L. M. Structure–Activity Relationship Studies of Functionally Selective Kappa Opioid Receptor Agonists that Modulate ERK 1/2 Phosphorylation While Preserving G Protein Over βArrestin2 Signaling Bias. ACS Chem. Neurosci. 2015, 6 (8), 1411–1419. 10.1021/acschemneuro.5b00092. 25891774 PMC4830356

[ref64] Nagase H.; Fujii H. Essential Structure of the κ Opioid Receptor Agonist Nalfurafine for Binding to the κ Receptor. Curr. Pharm. Des. 2014, 19, 7400–7414. 10.2174/138161281942140105165011. 23448474

[ref65] Nagase H.; Imaide S.; Yamada T.; Hirayama S.; Nemoto T.; Yamaotsu N.; Hirono S.; Fujii H. Essential Structure of Opioid κ Receptor Agonist Nalfurafine for Binding to κ Receptor 1: Synthesis of Decahydroisoquinoline Derivatives and Their Pharmacologies. Chem. Pharm. Bull. (Tokyo) 2012, 60 (8), 945–948. 10.1248/cpb.c12-00336. 22863695

[ref66] Nagase H.; Imaide S.; Hirayama S.; Nemoto T.; Fujii H. Essential structure of opioid κ receptor agonist nalfurafine for binding to the κ receptor 2: Synthesis of decahydro(iminoethano)phenanthrene derivatives and their pharmacologies. Bioorg. Med. Chem. Lett. 2012, 22, 5071–5074. 10.1016/j.bmcl.2012.05.122. 22742909

[ref67] Nagase H.; Akiyama J.; Nakajima R.; Hirayama S.; Nemoto T.; Gouda H.; Hirono S.; Fujii H. Synthesis of New Opioid Derivatives with a Propellane Skeleton and Their Pharmacology. Part 2: Propellane Derivatives with an Amide Side Chain. Bioorg. Med. Chem. Lett. 2012, 22, 2775 10.1016/j.bmcl.2012.02.082. 22460026

[ref68] Nakajima R.; Yamamoto N.; Hirayama S.; Iwai T.; Saitoh A.; Nagumo Y.; Fujii H.; Nagase H. Synthesis of New Opioid Derivatives with a Propellane Skeleton and Their Pharmacologies: Part 5, Novel Pentacyclic Propellane Derivatives with a 6-Amide Side Chain. Bioorg. Med. Chem. 2015, 23, 6271–6279. 10.1016/j.bmc.2015.08.036. 26346669

[ref69] Kawai K.; Hayakawa J.; Miyamoto T.; Imamura Y.; Yamane S.; Wakita H.; Fujii H.; Kawamura K.; Matsuura H.; Izumimoto N.; Kobayashi R.; Endo T.; Nagase H. Design, synthesis, and structure–activity relationship of novel opioid κ-agonists. Bioorg. Med. Chem. 2008, 16, 9188–9201. 10.1016/j.bmc.2008.09.011. 18829333

[ref70] Horikiri H.; Hirano N.; Tanaka Y.; Oishi J.; Hatakeyama H.; Kawamura K.; Nagase H. Syntheses of 10-Oxo, 10.ALPHA.-Hydroxy, and 10.BETA.-Hydroxy Derivatives of a Potent .KAPPA.-Opioid Receptor Agonist, TRK-820. Chem. Pharm. Bull. (Tokyo) 2004, 52 (6), 664–669. 10.1248/cpb.52.664. 15187385

[ref71] Suzuki S.; Sugawara Y.; Tsuji R.; Tanimura R.; Kaneko C.; Yuzawa N.; Yagi M.; Kawai K. Discovery of highly selective κ-opioid receptor agonists: 10α-Hydroxy TRK-820 derivatives. Bioorg. Med. Chem. Lett. 2017, 27, 3920–3924. 10.1016/j.bmcl.2017.06.017. 28688957

[ref72] Nemoto T.; Yamamoto N.; Wada N.; Harada Y.; Tomatsu M.; Ishihara M.; Hirayama S.; Iwai T.; Fujii H.; Nagase H. The effect of 17-N substituents on the activity of the opioid κ receptor in nalfurafine derivatives. Bioorg. Med. Chem. Lett. 2013, 23, 268–272. 10.1016/j.bmcl.2012.10.100. 23200250

[ref73] Cashman J. Synthesis of metabolically stable agents for alcohol and drug abuse. WO 2010006119 Al, 2010.

[ref74] Watanabe Y.; Kitazawa S.; Fujii H.; Nemoto T.; Hirayama S.; Iwai T.; Gouda H.; Hirono S.; Nagasea H. Design, Synthesis, and Structure-Activity Relationship of Novel Opioid κ Receptor Selective Agonists: α-Iminoamide Derivatives with an Azabicyclo[2.2.2]Octene Skeleton. Bioorg. Med. Chem. Lett. 2014, 24 (21), 4980–4983. 10.1016/j.bmcl.2014.09.029. 25283554

[ref75] Nagase H.; Watanabe A.; Nemoto T.; Yamaotsu N.; Hayashida K.; Nakajima M.; Hasebe K.; Nakao K.; Mochizuki H.; Hirono S.; Fujii H. Drug Design and Synthesis of a Novel κ Opioid Receptor Agonist with an Oxabicyclo[2.2.2]Octane Skeleton and Its Pharmacology. Bioorg. Med. Chem. Lett. 2010, 20 (1), 121–124. 10.1016/j.bmcl.2009.11.027. 19962305

[ref76] Watanabe A.; Fujii H.; Nakajima M.; Hasebe K.; Mochizuki H.; Nagase H. Synthesis of a New Opioid Ligand Having the Oxabicyclo[3.2.1]Octane Skeleton Using a New Rearrangement Reaction. Bioorg. Med. Chem. Lett. 2009, 19 (9), 2416–2419. 10.1016/j.bmcl.2009.03.068. 19349178

[ref77] Wang H.; Cao D.; Gillespie J. C.; Mendez R. E.; Selley D. E.; Liu-Chen L.-Y.; Zhang Y. Exploring the Putative Mechanism of Allosteric Modulations by Mixed-Action Kappa/Mu Opioid Receptor Bitopic Modulators. Future Med. Chem. 2021, 13, 551–573. 10.4155/fmc-2020-0308. 33590767 PMC8027703

[ref78] Pagare P. P.; Li M.; Zheng Y.; Kulkarni A. S.; Obeng S.; Huang B.; Ruiz C.; Gillespie J. C.; Mendez R. E.; Stevens D. L.; Poklis J. L.; Halquist M. S.; Dewey W. L.; Selley D. E.; Zhang Y. Design, Synthesis, and Biological Evaluation of NAP Isosteres: A Switch from Peripheral to Central Nervous System Acting Mu-Opioid Receptor Antagonists. J. Med. Chem. 2022, 65 (6), 5095–5112. 10.1021/acs.jmedchem.2c00087. 35255685 PMC10149103

[ref79] ACD Percepta Software Reference: ACD/Percepta. version 2020.1.2; Advanced Chemistry Development, Inc.: Toronto, ON, Canada, 2020. Www.Acdlabs.Com.

[ref80] Waring M. J. Lipophilicity in Drug Discovery. Expert. Opin. Drug Discov. 2010, 5 (3), 235–248. 10.1517/17460441003605098. 22823020

[ref81] Lipinski C. A.; Lombardo F.; Dominy B. W.; Feeney P. J. Experimental and Computational Approaches to Estimate Solubility and Permeability in Drug Discovery and Development Settings. Adv. Drug Deliv. Rev. 2001, 46, 3–26. 10.1016/s0169-409x(00)00129-0. 11259830

[ref82] Manallack D. T. The PKa Distribution of Drugs: Application to Drug Discovery. Perspect. Med. Chem. 2007, 1, 25–38. PMC2754920 19812734

[ref83] Ertl P.; Rohde B.; Selzer P. Fast Calculation of Molecular Polar Surface Area as a Sum of Fragment-Based Contributions and Its Application to the Prediction of Drug Transport Properties. J. Med. Chem. 2000, 43 (20), 3714–3717. 10.1021/jm000942e. 11020286

[ref84] Ida Y.; Nemoto T.; Hirayama S.; Fujii H.; Osa Y.; Imai M.; Nakamura T.; Kanemasa T.; Kato A.; Nagase H. Synthesis of Quinolinomorphinan-4-Ol Derivatives as δ Opioid Receptor Agonists. Bioorg. Med. Chem. 2012, 20 (2), 949–961. 10.1016/j.bmc.2011.11.047. 22197670

[ref85] Obeng S.; Yuan Y.; Jali A.; Selley D. E.; Zhang Y. In vitro and in vivo functional profile characterization of 17-cyclopropylmethyl-3,14β-dihydroxy-4,5α-epoxy-6α-(isoquinoline-3-carboxamido)morphinan (NAQ) as a low efficacy mu opioid receptor modulator. Eur. J. Pharmacol. 2018, 827, 32–40. 10.1016/j.ejphar.2018.03.013. 29530590 PMC5890425

[ref86] Zheng Y.; Obeng S.; Wang H.; Jali A. M.; Peddibhotla B.; Williams D. A.; Zou C.; Stevens D. L.; Dewey W. L.; Akbarali H. I.; Selley D. E.; Zhang Y. Design, Synthesis, and Biological Evaluation of the Third Generation 17-Cyclopropylmethyl-3,14β-dihydroxy-4,5α-epoxy-6β-[(4′-pyridyl)carboxamido]morphinan (NAP) Derivatives as μ/κ Opioid Receptor Dual Selective Ligands. J. Med. Chem. 2019, 62 (2), 561–574. 10.1021/acs.jmedchem.8b01158. 30608693 PMC6467700

[ref87] Obeng S.; Jali A.; Zheng Y.; Wang H.; Schwienteck K. L.; Chen C.; Stevens D. L.; Akbarali H. I.; Dewey W. L.; Banks M. L.; Liu-Chen L. Y.; Selley D. E.; Zhang Y. Characterization of 17-Cyclopropylmethyl-3,14β-dihydroxy-4,5α-epoxy-6α-(indole-7-carboxamido)morphinan (NAN) as a Novel Opioid Receptor Modulator for Opioid Use Disorder Treatment. ACS Chem. Neurosci. 2019, 10 (5), 2518–2532. 10.1021/acschemneuro.9b00038. 30758946 PMC6520168

[ref88] Ma H.; Obeng S.; Wang H.; Zheng Y.; Li M.; Jali A. M.; Stevens D. L.; Dewey W. L.; Selley D. E.; Zhang Y. Application of Bivalent Bioisostere Concept on Design and Discovery of Potent Opioid Receptor Modulators. J. Med. Chem. 2019, 62 (24), 11399–11415. 10.1021/acs.jmedchem.9b01767. 31782922

[ref89] Zheng Y.; Obeng S.; Reinecke B. A.; Chen C.; Phansalkar P. S.; Walentiny D. M.; Gerk P. M.; Liu-Chen L. Y.; Selley D. E.; Beardsley P. M.; Zhang Y. Pharmacological Characterization of 17-Cyclopropylmethyl-3,14-Dihydroxy-4,5-Epoxy-6-[(3′-Fluoro-4′-Pyridyl)Acetamido]Morphinan (NFP) as a Dual Selective MOR/KOR Ligand with Potential Applications in Treating Opioid Use Disorder. Eur. J. Pharmacol. 2019, 865, 172812 10.1016/j.ejphar.2019.172812. 31743739 PMC6914219

[ref90] Yuan Y.; Elbegdorj O.; Chen J.; Akubathini S. K.; Zhang F.; Stevens D. L.; Beletskaya I. O.; Scoggins K. L.; Zhang Z.; Gerk P. M.; Selley D. E.; Akbarali H. I.; Dewey W. L.; Zhang Y. Design, Synthesis, and Biological Evaluation of 17-Cyclopropylmethyl-3,14β-dihydroxy-4,5α-epoxy-6β-[(4′-pyridyl)carboxamido]morphinan Derivatives as Peripheral Selective μ Opioid Receptor Agents. J. Med. Chem. 2012, 55 (22), 10118–10129. 10.1021/jm301247n. 23116124 PMC3527108

[ref91] Yuan Y.; Elbegdorj O.; Beletskaya I. O.; Selley D. E.; Zhang Y. Structure activity relationship studies of 17-cyclopropylmethyl-3,14β-dihydroxy-4,5α-epoxy-6α-(isoquinoline-3′-carboxamido)morphinan (NAQ) analogues as potent opioid receptor ligands: Preliminary results on the role of electronic characteristics for affinity and function. Bioorg. Med. Chem. Lett. 2013, 23 (18), 5045–5048. 10.1016/j.bmcl.2013.07.043. 23948248 PMC3776595

[ref92] Yuan Y.; Stevens D. L.; Braithwaite A.; Scoggins K. L.; Bilsky E. J.; Akbarali H. I.; Dewey W. L.; Zhang Y. 6β-N-Heterocyclic substituted naltrexamine derivative NAP as a potential lead to develop peripheral mu opioid receptor selective antagonists. Bioorg. Med. Chem. Lett. 2012, 22 (14), 4731–4734. 10.1016/j.bmcl.2012.05.075. 22683223 PMC3395321

[ref93] Yuan Y.; Elbegdorj O.; Chen J.; Akubathini S. K.; Beletskaya I. O.; Selley D. E.; Zhang Y. Structure selectivity relationship studies of 17-cyclopropylmethyl-3,14β-dihydroxy-4,5α-epoxy-6β-[(4′-pyridyl)carboxamido]morphinan derivatives toward the development of the mu opioid receptor antagonists. Bioorg. Med. Chem. Lett. 2011, 21 (18), 5625–5629. 10.1016/j.bmcl.2011.06.135. 21788135 PMC3171173

[ref94] Zhang Y.; Braithwaite A.; Yuan Y.; Streicher J. M.; Bilsky E. J. Behavioral and cellular pharmacology characterization of 17-cyclopropylmethyl-3,14β-dihydroxy-4,5α-epoxy-6α-(isoquinoline-3′-carboxamido)morphinan (NAQ) as a mu opioid receptor selective ligand. Eur. J. Pharmacol. 2014, 736, 124–130. 10.1016/j.ejphar.2014.04.041. 24815322 PMC4073486

[ref95] Zhang Y.; Williams D. A.; Zaidi S. A.; Yuan Y.; Braithwaite A.; Bilsky E. J.; Dewey W. L.; Akbarali H. I.; Streicher J. M.; Selley D. E. 17-Cyclopropylmethyl-3,14β-dihydroxy-4,5α-epoxy-6β-(4′-pyridylcarboxamido)morphinan (NAP) Modulating the Mu Opioid Receptor in a Biased Fashion. ACS Chem. Neurosci. 2016, 7 (3), 297–304. 10.1021/acschemneuro.5b00245. 26716358 PMC5111356

[ref96] Yuan Y.; Zaidi S. A.; Stevens D. L.; Scoggins K. L.; Mosier P. D.; Kellogg G. E.; Dewey W. L.; Selley D. E.; Zhang Y. Design, Syntheses and Pharmacological Characterization of 17-Cyclopropylmethyl-3,14beta-Dihydroxy-4,5alpha-Epoxy-6alpha-(Isoquinoline-3′-Carboxamido)Morphinan Analogues as Opioid Receptor Ligands. Physiol. Behav. 2017, 176 (1), 139–148. 10.1016/j.bmc.2015.02.055. 25783191 PMC4380750

[ref97] Li M.; St. Onge C. M.; Zhang Y. Stereoselective Syntheses of 3-Dehydroxynaltrexamines and N-Methyl-3-Dehydroxynaltrexamines. Tetrahedron Lett. 2020, 61 (39), 152379 10.1016/j.tetlet.2020.152379. 33100417 PMC7584067

[ref98] Thinnes C. C.; Lohans C. T.; Abboud M. I.; Yeh T.; Tumber A.; Nowak R. P.; Attwood M.; Cockman M. E.; Oppermann U.; Loenarz C.; Schofield C. J. Selective Inhibitors of a Human Prolyl Hydroxylase (OGFOD1) Involved in Ribosomal Decoding. Chem.—Eur. J. 2019, 25, 2019–2024. 10.1002/chem.201804790. 30427558 PMC6471485

[ref99] Li G.; Aschenbach L. C.; Chen J.; Cassidy M. P.; Stevens D. L.; Gabra B. H.; Selley D. E.; Dewey W. L.; Westkaemper R. B.; Zhang Y. Design, Synthesis, and Biological Evaluation of 6α- and 6β-*N*-Heterocyclic Substituted Naltrexamine Derivatives as μ Opioid Receptor Selective Antagonists. J. Med. Chem. 2009, 52 (5), 1416–1427. 10.1021/jm801272c. 19199782 PMC2880636

[ref100] Yuan Y.; Zaidi S. A.; Stevens D. L.; Scoggins K. L.; Mosier P. D.; Kellogg G. E.; Dewey W. L.; Selley D. E.; Zhang Y. Design, syntheses, and pharmacological characterization of 17-cyclopropylmethyl-3,14β-dihydroxy-4,5α-epoxy-6α-(isoquinoline-3′-carboxamido)morphinan analogues as opioid receptor ligands. Bioorg. Med. Chem. 2015, 23 (8), 1701–1715. 10.1016/j.bmc.2015.02.055. 25783191 PMC4380750

[ref101] Obeng S.; Wang H.; Jali A.; Stevens D. L.; Akbarali H. I.; Dewey W. L.; Selley D. E.; Zhang Y. Structure–Activity Relationship Studies of 6α- and 6β-Indolylacetamidonaltrexamine Derivatives as Bitopic Mu Opioid Receptor Modulators and Elaboration of the “Message-Address Concept” To Comprehend Their Functional Conversion. ACS Chem. Neurosci. 2019, 10 (3), 1075–1090. 10.1021/acschemneuro.8b00349. 30156823 PMC6405326

[ref102] Che T.; Majumdar S.; Zaidi S. A.; Ondachi P.; Mccorvy J. D.; Wang S.; Mosier P. D.; Uprety R.; Vardy E.; Krumm B. E.; Han G. W.; Lee M.; Pardon E.; Steyaert J.; Huang X.; Strachan R. T.; Tribo A. R.; Pasternak G. W.; Carroll F. I.; Stevens R. C.; et al. Structure of the Nanobody-Stabilized Active State of the Kappa Opioid Receptor. Cell 2018, 172, 55–67.e15. 10.1016/j.cell.2017.12.011. 29307491 PMC5802374

[ref103] Pert C. B.; Pasternak G.; Snyder S. H. Opiate Agonists and Antagonists Discriminated by Receptor Binding in Brain. Science 1973, 182, 1359 10.1126/science.182.4119.1359. 4128222

[ref104] Pasternak G. W.; Pan Y. X. Mu Opioids and Their Receptors: Evolution of a Concept. Pharmacol. Rev. 2013, 65, 1257–1317. 10.1124/pr.112.007138. 24076545 PMC3799236

[ref105] Li G.; Aschenbach L. C. K.; He H.; Selley D. E.; Zhang Y. 14-O-Heterocyclic-Substituted Naltrexone Derivatives as Non-Peptide Mu Opiid Receptor Selective Antagonists: Design, Synthesis, and Biological Studies. Bioorg. Med. Chem. Lett. 2009, 19 (6), 1825–1839. 10.1016/j.bmcl.2008.12.093. 19217280 PMC2802822

[ref106] Gomaa A. A.; Moustafa S. A.; Mohamed L. H.; Ahmed H. N. Modification of Morphine-Induced Analgesia, Tolerance and Dependence by Bromocriptine. J. Islam. Acad. Sci. 1990, 3 (1), 22–26. 10.1016/0014-2999(89)90533-5 2620694

[ref107] Huskinson S. L.; Platt D. M.; Brasfield M.; Follett M. E.; Prisinzano T. E.; Blough B. E.; Freeman K. B. Quantification of Observable Behaviors Induced by Typical and Atypical Kappa-Opioid Receptor Agonists in Male Rhesus Monkeys. Psychopharmacology (Berl) 2020, 237 (7), 2075–2087. 10.1007/s00213-020-05519-7. 32372348 PMC7308209

[ref108] Lazenka M. L.; Moerke M. J.; Townsend E. A.; Freeman K. B.; Carroll F. I.; Negus S. S. Dissociable Effects of the Kappa Opioid Receptor Agonist Nalfurafine on Pain/Itch-Stimulated and Pain/Itch-Depressed Behaviors in Male Rats. Psychopharmacology (Berl) 2018, 235 (1), 203–213. 10.1007/s00213-017-4758-7. 29063139 PMC5750069

[ref109] Sakakihara M.; Imamachi N.; Saito Y. Effects of Intrathecal κ-Opioid Receptor Agonist on Morphine-Induced Itch and Antinociception in Mice. Reg. Anesth. Pain Med. 2016, 41 (1), 69–74. 10.1097/AAP.0000000000000326. 26587674

[ref110] Ward S. J.; Portoghese P. S.; Takemori A. E.; Chemistry M. Pharmacological Characterization in Vivo of the Novel Opiate, Fl-Funaltrexamine1. J. Pharmacol. Exp. Ther. 1982, 220, 494. 6121045

[ref111] Takemori A. E.; Ho B. Y.; Naeseth J. S.; Portoghese P. Nor-Binaltorphimine, a Highly Selective Kappa-Opioid Antagonist in Analgesic and Receptor Binding Assays’. J. Pharmacol. Exp. Ther. 1988, 246, 255. 2839664

[ref112] Portoghese P. S.; Sultana M.; Takemori A. E. Rapid Communication Naltrindole, a Highly Selective and Potent Non-peptide 8 Opioid Receptor Antagonist, 1988; Vol. 146. 10.1016/0014-2999(88)90502-x 2832195

[ref113] Zhou Y.; Freeman K.; Setola V.; Cao D.; Kaski S.; Kreek M. J.; Liu-Chen L. Y. Preclinical Studies on Nalfurafine (TRK-820), a Clinically Used KOR Agonist. In Handbook of Experimental Pharmacology; Springer Science and Business Media Deutschland GmbH, 2022; Vol. 271, pp 137–162. 33834276 10.1007/164_2021_443 PMC9249371

[ref114] Liu-Chen L.-Y.; Huang P. Signaling Underlying Kappa Opioid Receptor-Mediated Behaviors in Rodents. Front. Neurosci. 2022, 16, 1–16. 10.3389/fnins.2022.964724. PMC9670127 36408401

[ref115] Yaksh T. L.; Woller S. A.; Ramachandran R.; Sorkin L. S. The Search for Novel Analgesics: Targets and Mechanisms. F1000Prime Rep. 2015, 7, 56 10.12703/P7-56. 26097729 PMC4447049

[ref116] Townsend E. A.; Negus S. S.; Caine S. B.; Thomsen M.; Banks M. L. Sex Differences in Opioid Reinforcement under a Fentanyl vs. Food Choice Procedure in Rats. Neuropsychopharmacology 2019, 44 (12), 2022–2029. 10.1038/s41386-019-0356-1. 30818323 PMC6898628

[ref117] Townsend E. A.; Schwienteck K. L.; Robinson H. L.; Lawson S. T.; Banks M. L. A Drug-vs-Food “Choice” Self-Administration Procedure in Rats to Investigate Pharmacological and Environmental Mechanisms of Substance Use Disorders. J. Neurosci. Methods 2021, 354, 109110 10.1016/j.jneumeth.2021.109110. 33705855 PMC8082467

[ref118] Banks M. L.; Negus S. S. Preclinical Determinants of Drug Choice under Concurrent Schedules of Drug Self-Administration. Adv. Pharmacol. Sci. 2012, 2012, 1–17. 10.1155/2012/281768. PMC3515886 23243420

[ref119] Nakao K.; Hirakata M.; Miyamoto Y.; Kainoh M.; Wakasa Y.; Yanagita T. Nalfurafine Hydrochloride, a Selective κ Opioid Receptor Agonist, Has No Reinforcing Effect on Intravenous Self-Administration in Rhesus Monkeys. Adv. Pharmacol. Sci. 2016, 130 (1), 8–14. 10.1016/j.jphs.2015.11.008. 26786553

[ref120] Ueno Y.; Mori A.; Yanagita T. One Year Long-Term Study on Abuse Liability of Nalfurafine in Hemodialysis Patients. Int. J. Clin. Pharmacol. Ther. 2013, 51 (11), 823–831. 10.5414/CP201852. 24040851

[ref121] Wang Y. H.; Sun J. F.; Tao Y. M.; Chi Z. Q.; Liu J. G. The role of κ-opioid receptor activation in mediating antinociception and addiction. Acta Pharmacol. Sin. 2010, 31, 1065–1070. 10.1038/aps.2010.138. 20729876 PMC4002313

[ref122] Kivell B. M.; Ewald A. W. M.; Prisinzano T. E. Salvinorin A Analogues and Other Kappa-Opioid Receptor Compounds as Treatments for Cocaine Abuse. Adv. Pharmacol. 2014, 69, 481–511. 10.1016/B978-0-12-420118-7.00012-3. 24484985 PMC4128345

[ref123] Dalefield M. L.; Scouller B.; Bibi R.; Kivell B. M. The Kappa Opioid Receptor: A Promising Therapeutic Target for Multiple Pathologies. Front. Pharmacol 2022, 13, 837671 10.3389/fphar.2022.837671. 35795569 PMC9251383

[ref124] Trudeau M. C.; Warmke J. W.; Ganetzky B.; Robertson G. A. HERG, a Human Inward Rectifier in the Voltage-Gated Potassium Channel Family. Science (1979) 1995, 269, 92–95. 10.1126/science.7604285. 7604285

[ref125] Cheng Y.-C.; Prusoff W. H. Relationship between the Inhibition Constant and the Concentration of Inhibitor Which Causes 50 Per Cent Inhibition (Iso) of an Enzymatic Reaction*; Pergamon Press, 1973; Vol. 22. 10.1016/0006-2952(73)90196-2 4202581

[ref126] Schwienteck K. L.; Blake S.; Bremer P. T.; Poklis J. L.; Townsend E. A.; Negus S. S.; Banks M. L. Effectiveness and Selectivity of a Heroin Conjugate Vaccine to Attenuate Heroin, 6-Acetylmorphine, and Morphine Antinociception in Rats: Comparison with Naltrexone. Drug Alcohol Depend. 2019, 204, 107501 10.1016/j.drugalcdep.2019.06.006. 31479865 PMC6878171

[ref127] J Richardson S.; Bai A.; A Kulkarni A.; F Moghaddam M. Efficiency in Drug Discovery: Liver S9 Fraction Assay As a Screen for Metabolic Stability. Drug Metab. Lett. 2016, 10, 83–90. 10.2174/1872312810666160223121836. 26902079 PMC5405623

