# Supplementary material for: Systematic Structure–Activity Relationship Study of Nalfurafine Analogues toward Development of Potentially Nonaddictive Pain Management Treatments
Source: J Med Chem. 2024 May 30;67(11):9552–74. doi: 10.1021/acs.jmedchem.4c00646 (PMC11181328; doi:10.1021/acs.jmedchem.4c00646)

Supporting Information

Systematic Structure-Activity Relationship Study of Nalfurafine Analogs Towards Development of Potentially Non-addictive Pain Management Treatments

Celsey M. St. Onge[a], Piyusha P. Pagare[a], Yi Zheng[a], Michelle Arriaga[b], David L. Stevens[b], Rolando E. Mendez[b], Justin L. Poklis[c], Matthew S. Halquist[c], Dana E. Selley[b], William L. Dewey[b], Matthew L. Banks[b], Yan Zhang[abd*]

[a] Department of Medicinal Chemistry, Virginia Commonwealth University, 800 E. Leigh Street, Richmond, Virginia 23219, United States

[b] Department of Pharmacology and Toxicology, Virginia Commonwealth University, 410 North 12th Street, Richmond, Virginia 23298, United States

[c] Department of Pharmaceutics, Virginia Commonwealth University, 410 North 12th Street, Richmond, Virginia 23298, United States

[d] Institute for Drug and Alcohol Studies, 203 East Cary Street, Richmond, Virginia 23298, United States

Table of Contents

1. **Table S1.** Physiochemical property predictions made with ACD Percepta ®. 3
2. ^{1}H and ^{13}C NMR spectra of final compounds. 5
3. **Table S2.** Purity of final compounds. 29
4. HPLC chromatograms of final compounds. 30

* Corresponding author. Tel.: +1(804)828-0021 E-mail address: yzhang2@vcu.edu (Y.Zhang).

1. **Table S1.** Physiochemical property predictions made with ACD Percepta ®.

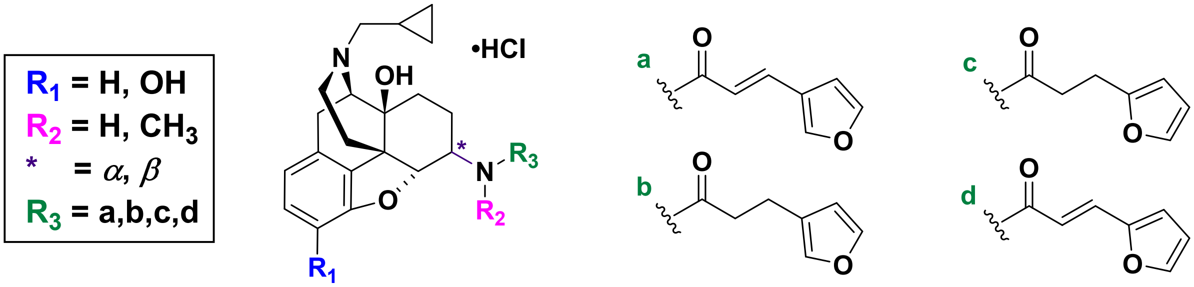

Compd.	Compd. Variations				ACD Percepta ® Predictions						
	R_1	R_2	α/β	R_3	Mol. Wt.	cLogP	cLogD pH 7.4	cpKa (Base)	HBD	HBA	TPSA
NLF	OH	CH_3	β	a	476.56	2.09	1.88	7.34	2	7	86.38
NMF	OH	CH_3	α	a	476.56	2.09	1.88	7.34	2	7	86.38
1	H	CH_3	α	b	462.58	3.64	3.04	7.44	1	6	66.15
2	H	H	α	b	448.55	2.92	2.48	7.44	2	6	74.94
3	H	CH_3	β	b	462.58	3.64	1.87	7.44	1	6	66.15
4	H	H	β	b	448.55	2.92	2.48	7.44	2	6	74.94
5	OH	CH_3	α	b	478.58	2.65	3.04	7.37	2	7	86.38
6	OH	H	α	b	464.55	2.47	1.84	7.37	3	7	95.17
7	OH	CH_3	β	b	478.58	2.65	3.04	7.37	2	7	86.38
8	OH	H	β	b	464.55	2.47	2.06	7.37	3	7	95.17
9	H	CH_3	α	c	462.58	3.64	2.44	7.43	1	6	66.15
10	H	H	α	c	448.55	2.92	2.09	7.43	2	6	74.94
11	H	CH_3	β	c	462.58	3.64	2.48	7.43	1	6	66.15
12	H	H	β	c	448.55	2.92	2.1	7.43	2	6	74.94
13	OH	CH_3	α	c	478.58	2.65	3.04	7.37	2	7	86.38
14	OH	H	α	c	464.55	2.47	2.09	7.37	3	7	95.17
15	OH	CH_3	β	c	478.58	2.65	2.48	7.37	2	7	86.38
16	OH	H	β	c	464.55	2.47	1.87	7.37	3	7	95.17
17	H	CH_3	α	d	460.56	2.64	2.75	7.39	1	6	66.15
18	H	H	α	d	446.54	2.35	1.87	7.39	2	6	74.94
19	H	CH_3	β	d	460.56	2.64	2.75	7.39	1	6	66.15
20	H	H	β	d	446.54	2.35	1.84	7.39	2	6	74.94
21	OH	CH_3	α	d	476.56	2.09	2.1	7.32	2	7	86.38
22	OH	H	α	d	462.54	1.92	2.06	7.32	3	7	95.17
23	OH	CH_3	β	d	476.56	2.09	2.44	7.32	2	7	86.38

24	OH	H	*β*	d	462.54	1.92	1.87	7.32	3	7	95.17

Table S1. Contd.

Mol. Wt.: Molecular weight (g/mol); cLogP: calculated partition coefficient; cLogD: calculated distribution constant (at physiological pH); cpKa: calculated negative logarithm of the acid dissociation constant; HBD: hydrogen bond donors; HBA: hydrogen bond acceptors; TPSA: topological polar surface area

2. ^{1}H and ^{13}C NMR spectra of final compounds.

17-Cyclopropylmethyl-4,5*α*-epoxy-6*α*-[3’-(furan-3”-yl)*N*-methylpropanamido]-14*β*-hydroxymorphinan hydrochloride **(1)**

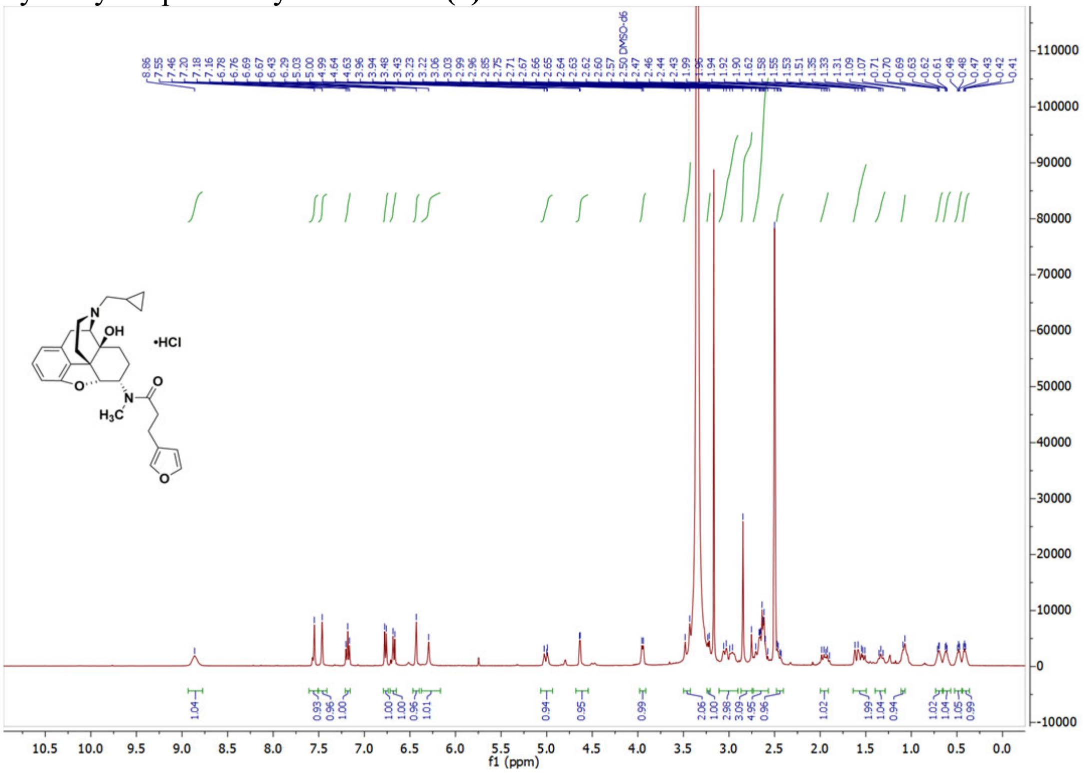

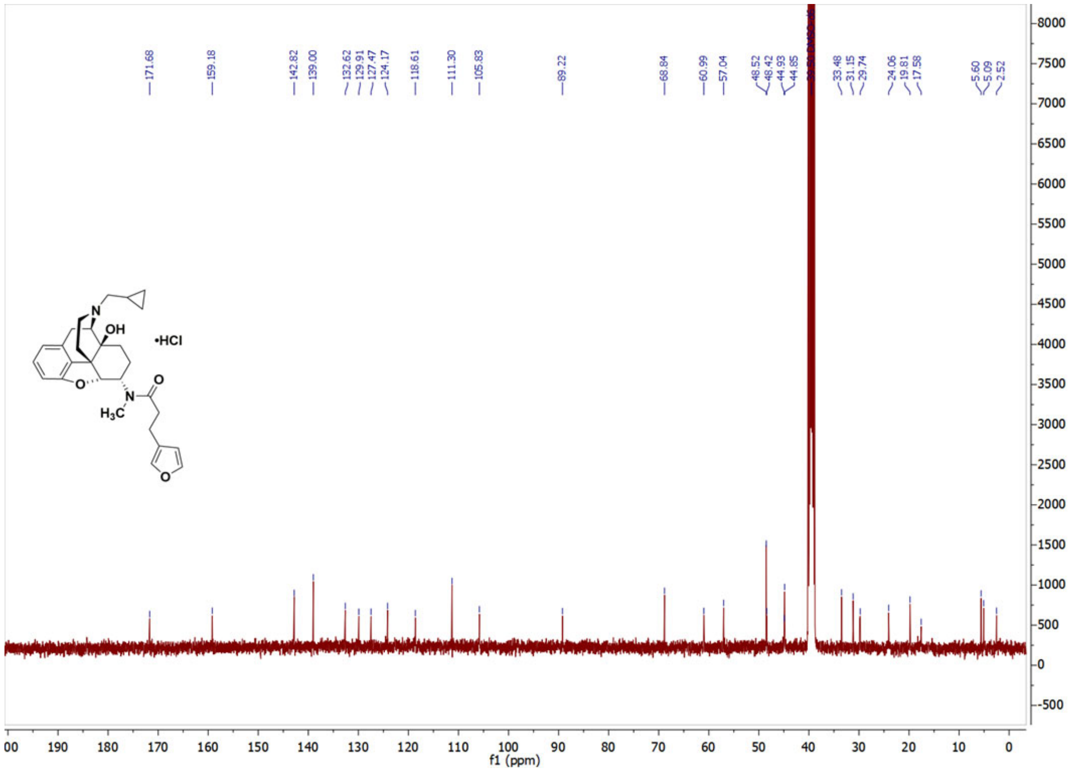

17-Cyclopropylmethyl-4,5α-epoxy-6α-[3’-(furan-3”-yl)propanamido]-14β-hydroxymorphinan hydrochloride **(2)**

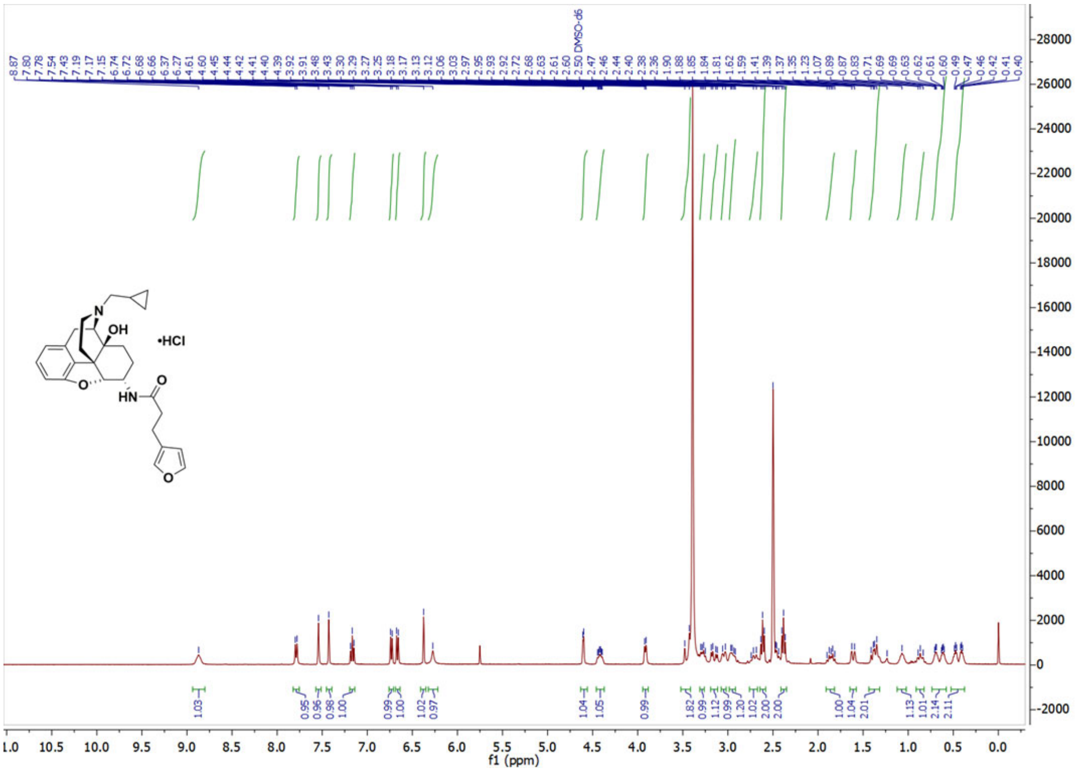

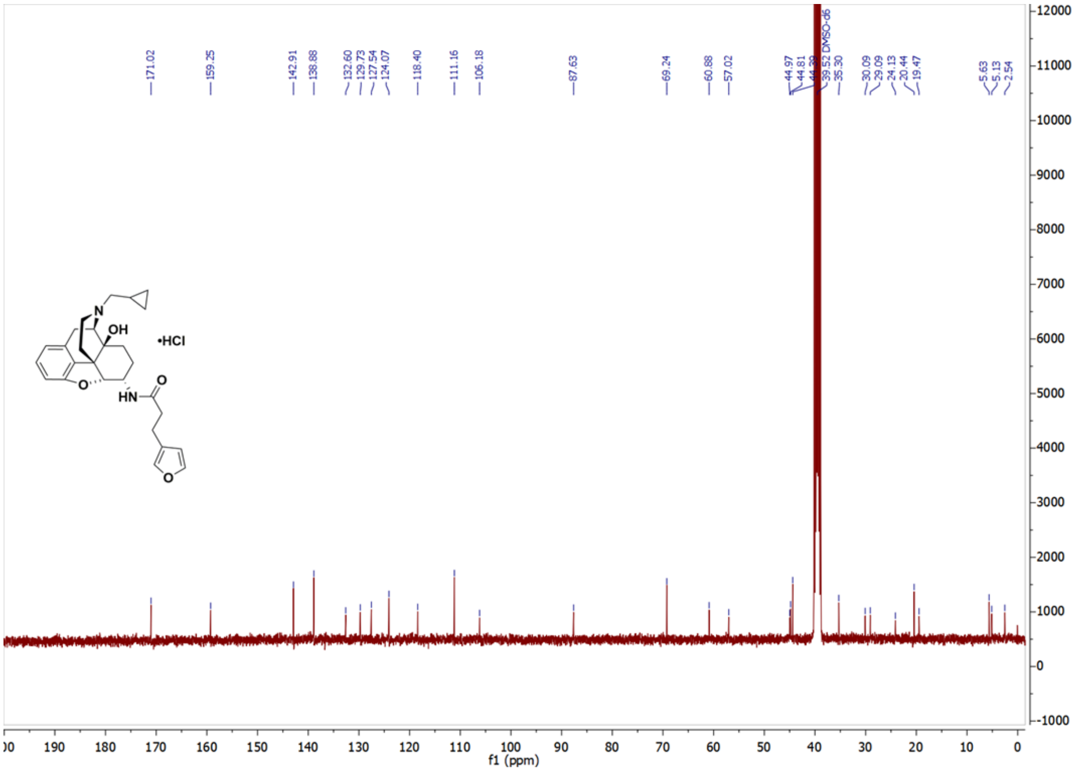

17-Cyclopropylmethyl-4,5α-epoxy-6β-[3'-(furan-3''-yl)-*N*-methylpropanamido]-14β-hydroxymorphinan hydrochloride **(3)**

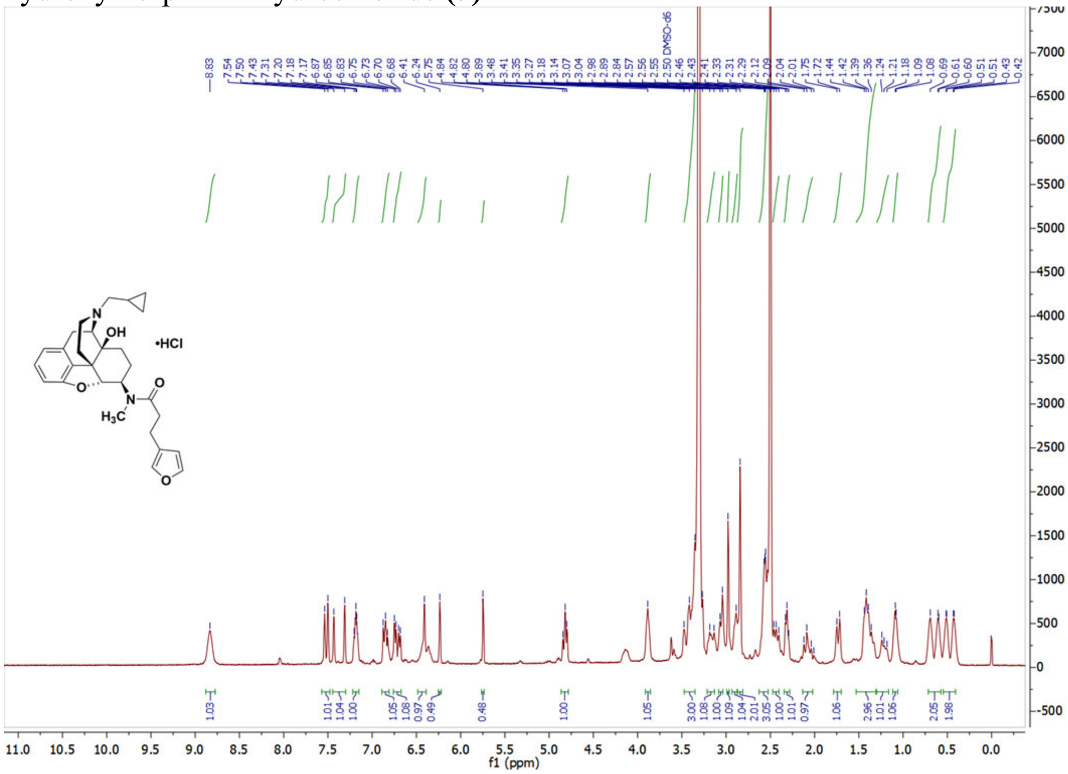

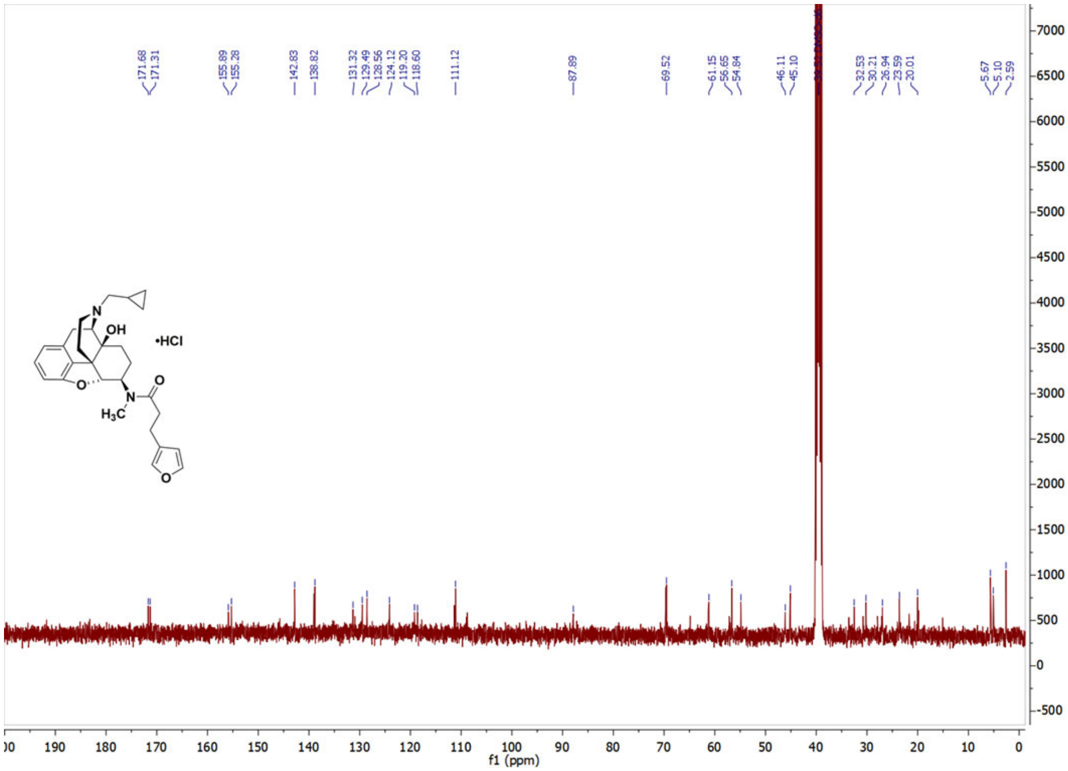

17-Cyclopropylmethyl-4,5*α*-epoxy-6*β*-[3’-(furan-3”-yl)propanamido]-14*β*-hydroxymorphinan hydrochloride **(4)**

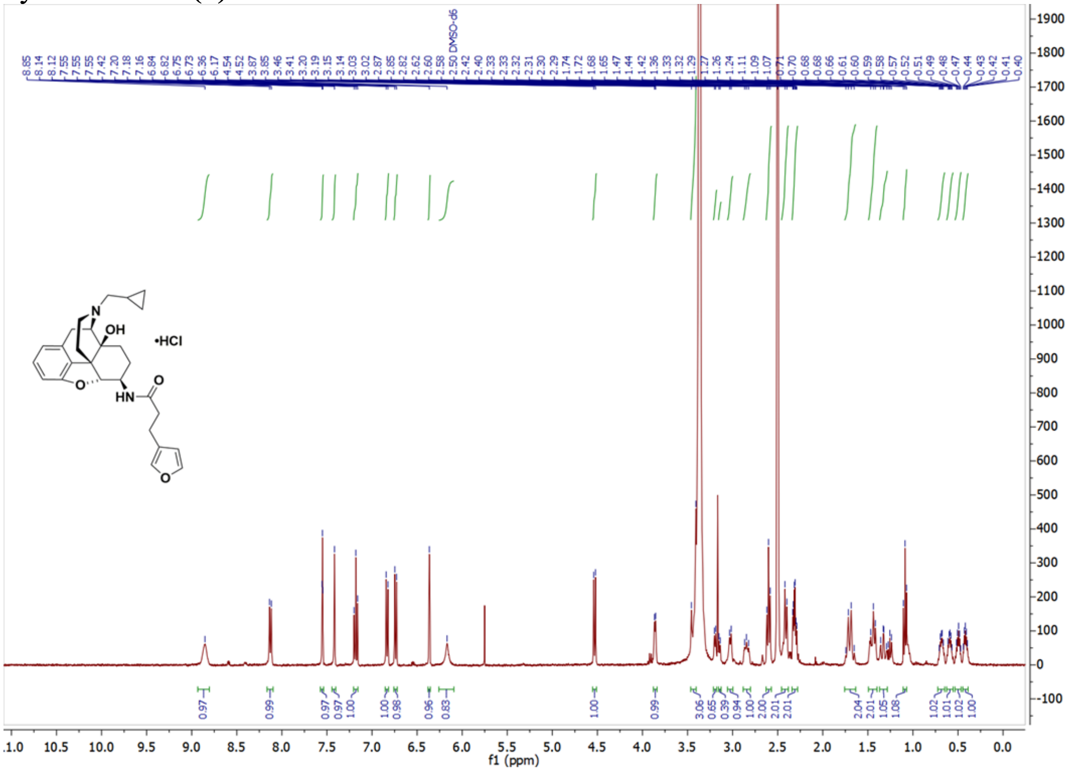

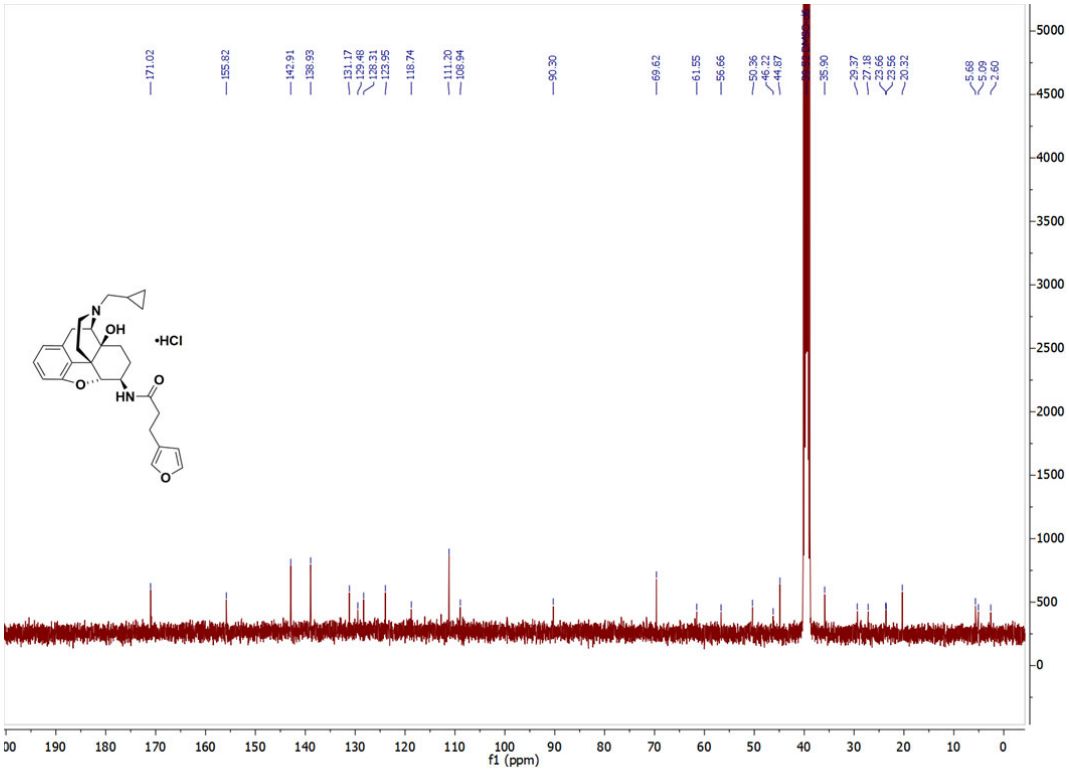

17-Cyclopropylmethyl-3,14β-dihydroxy-4,5α-epoxy-6α-[3'-(furan-3''-yl)-N-methylpropanamido]morphinan hydrochloride **(5)**

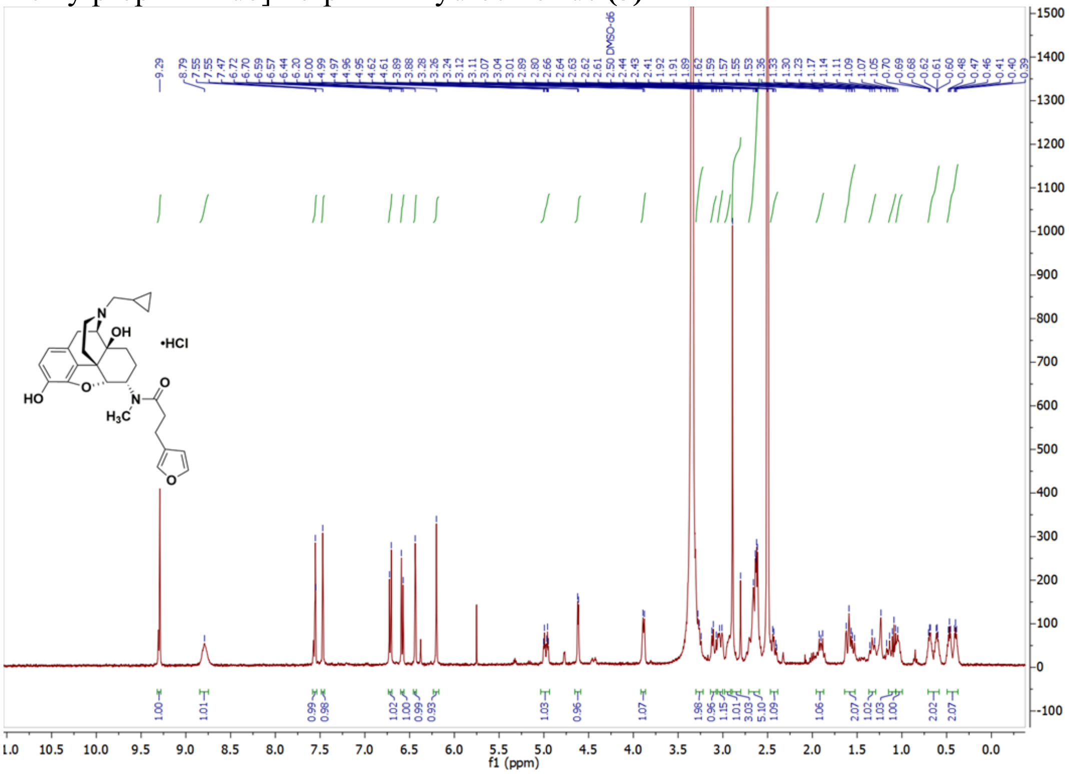

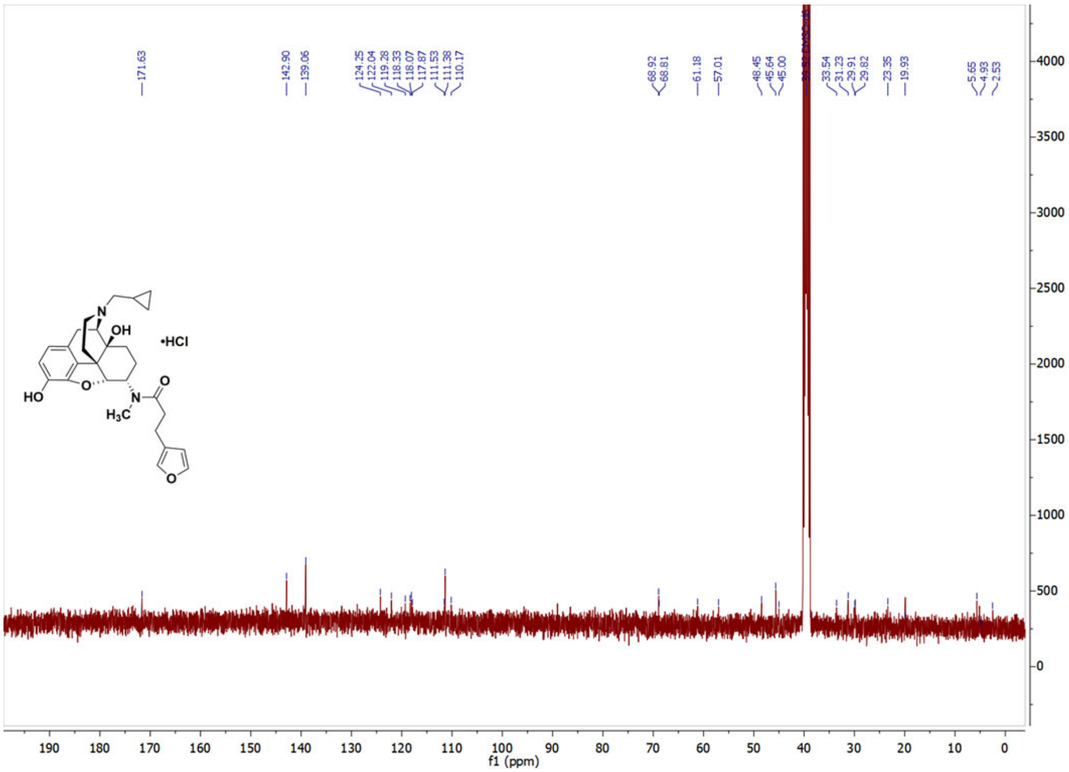

17-Cyclopropylmethyl-3,14*β*-dihydroxy-4,5*α*-epoxy-6*α*-[3’-(furan-3”-yl)propanamido]morphinan hydrochloride **(6)**

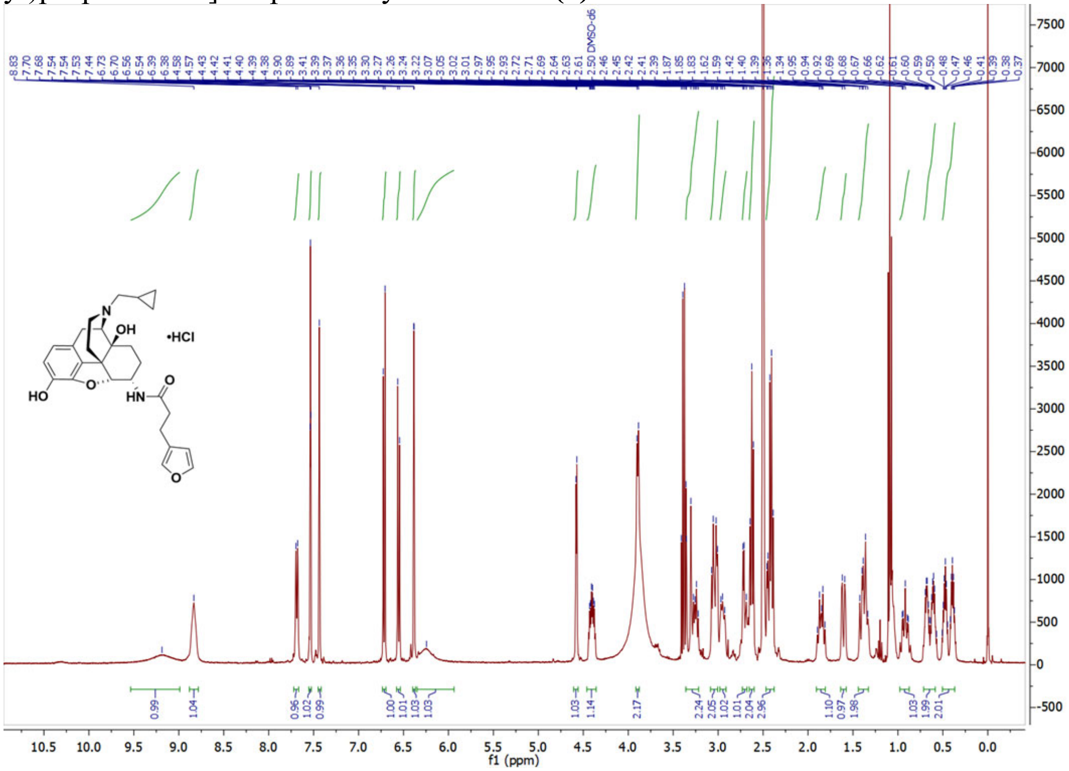

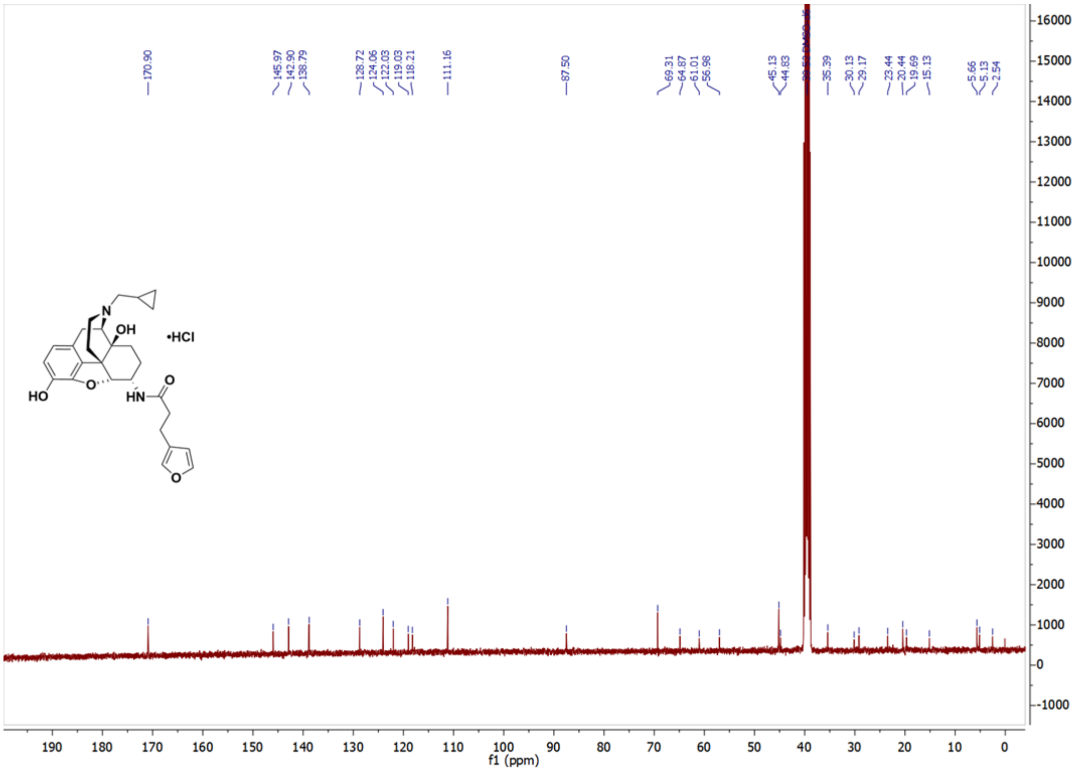

17-Cyclopropylmethyl-3,14*β*-dihydroxy-4,5*α*-epoxy-6*β*-[3'-(furan-3''-yl)-*N*-methylpropanamido]morphinan hydrochloride **(7)**

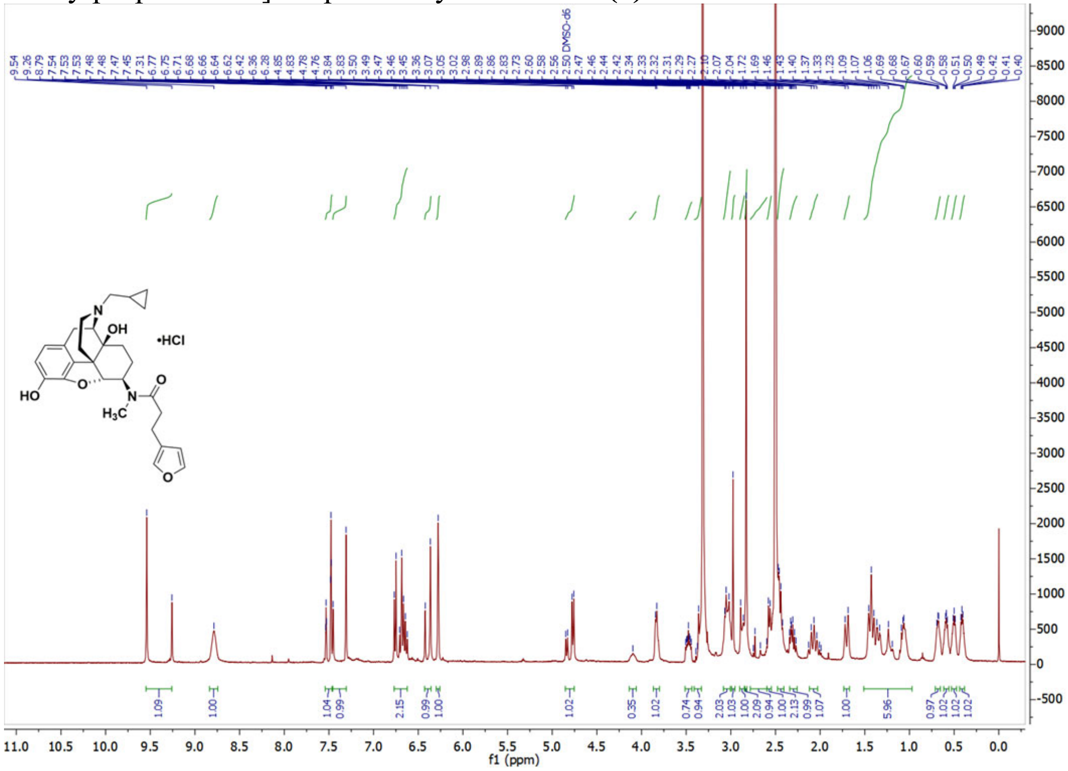

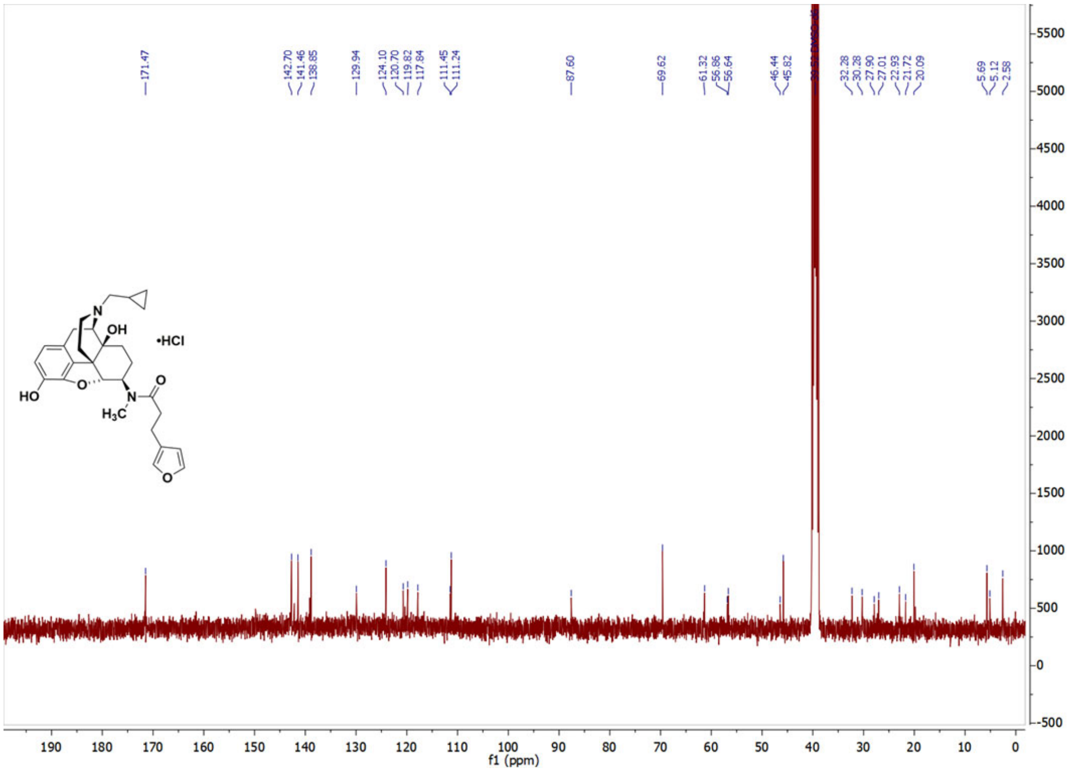

17-Cyclopropylmethyl-3,14β-dihydroxy-4,5α-epoxy-6β-[3'-(furan-3"-yl)propanamido]morphinan hydrochloride **(8)**

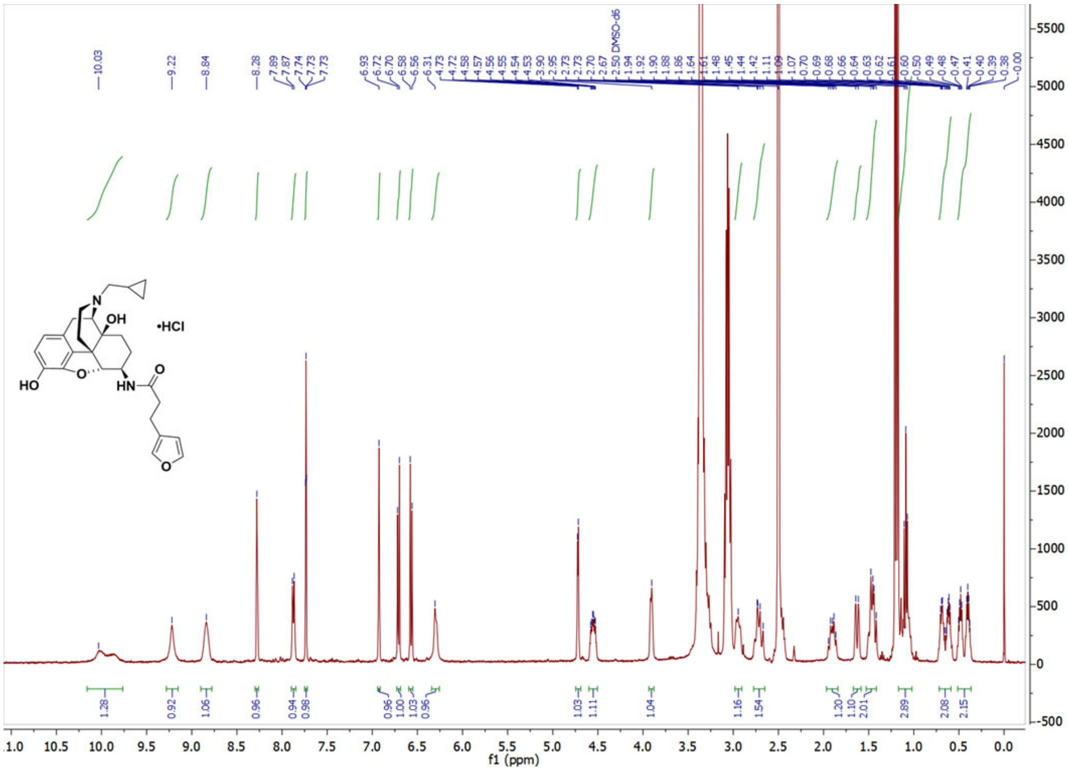

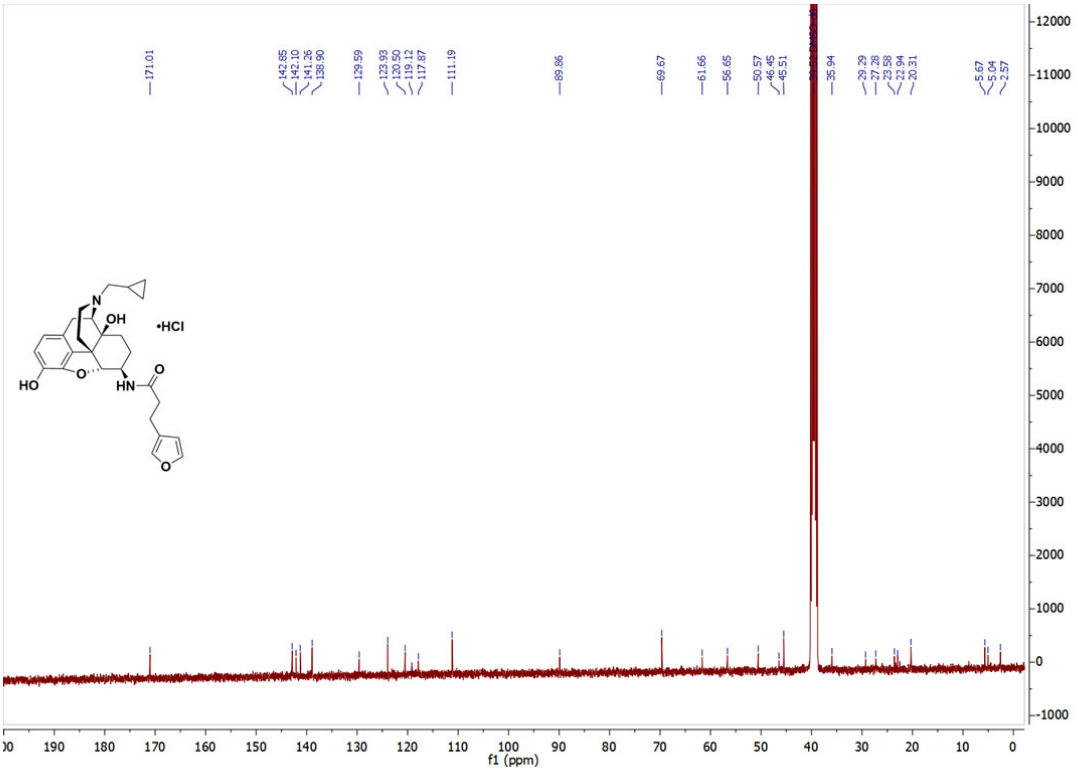

17-Cyclopropylmethyl-4,5*α*-epoxy-6*α*-[3’-(furan-2”-yl)*N*-methylpropanamido]-14*β*-hydroxymorphinan hydrochloride **(9)**

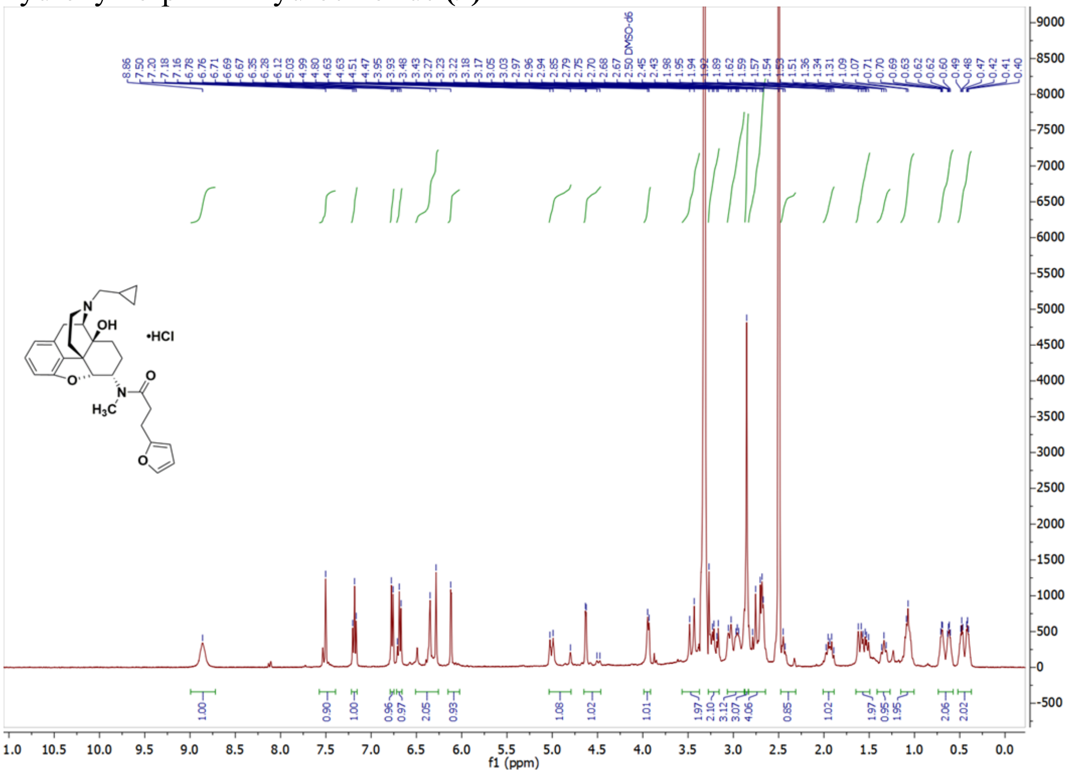

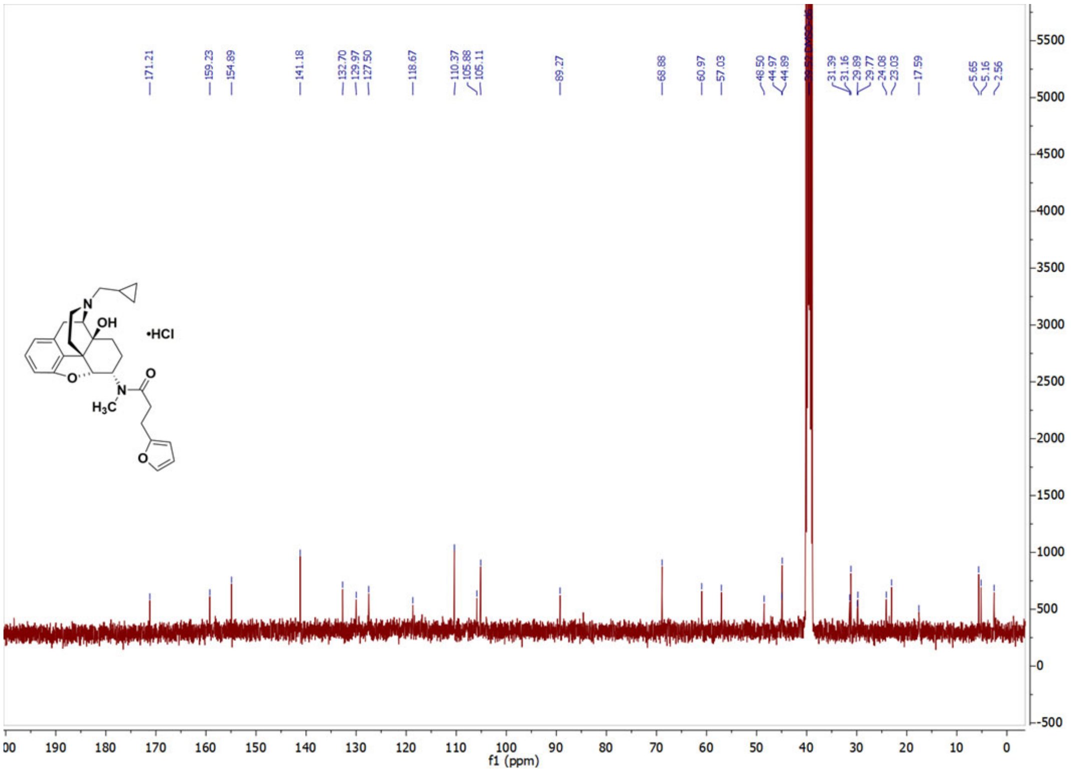

17-Cyclopropylmethyl-4,5α-epoxy-6α-[3’-(furan-2”-yl)propanamido]-14β-hydroxymorphinan hydrochloride **(10)**

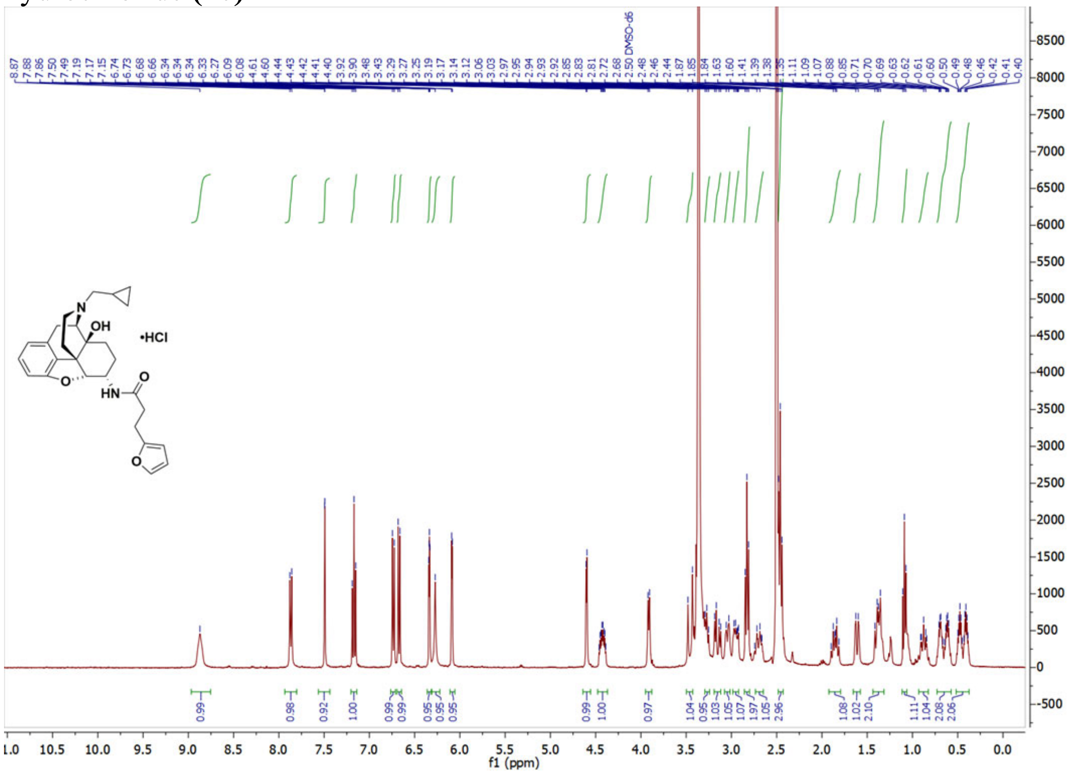

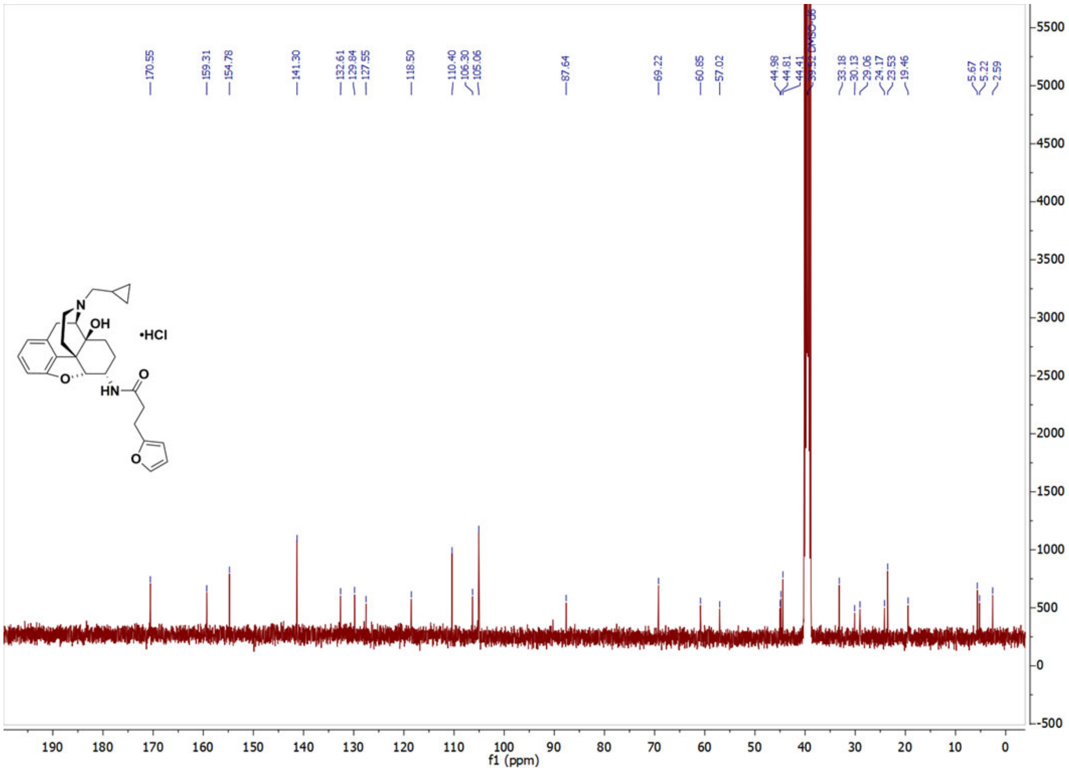

17-Cyclopropylmethyl-4,5α-epoxy-6β-[3'-(furan-2''-yl)-*N*-methylpropanamido]-14β-hydroxymorphinan hydrochloride **(11)**

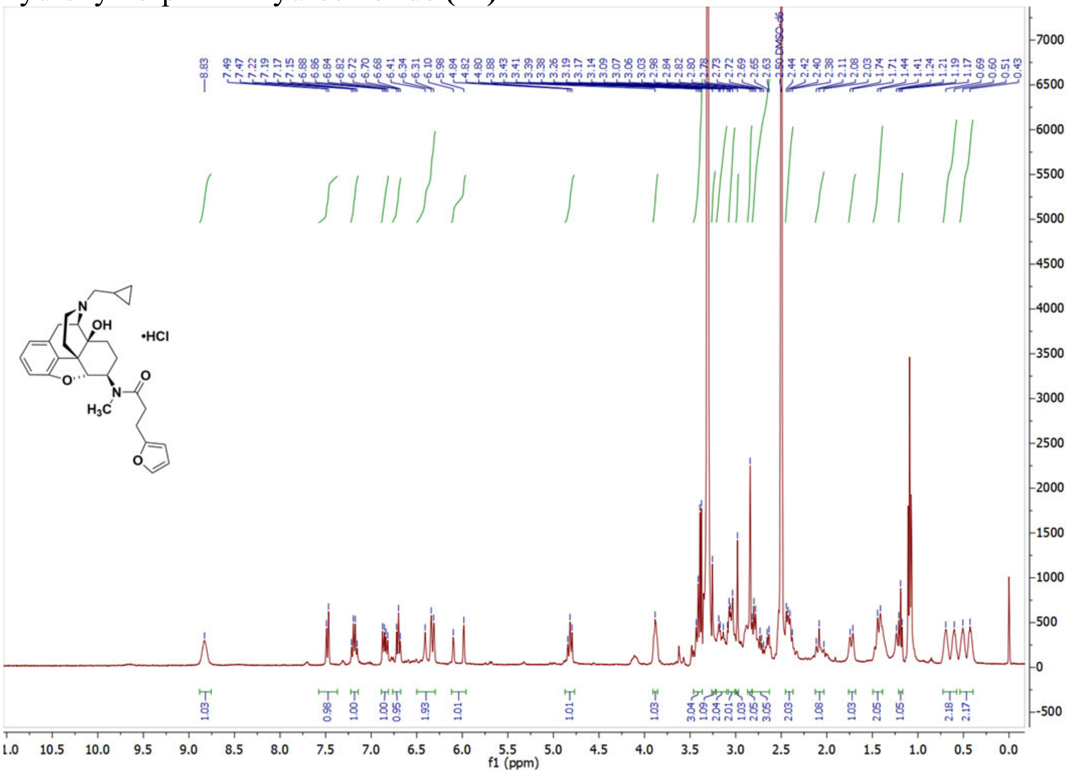

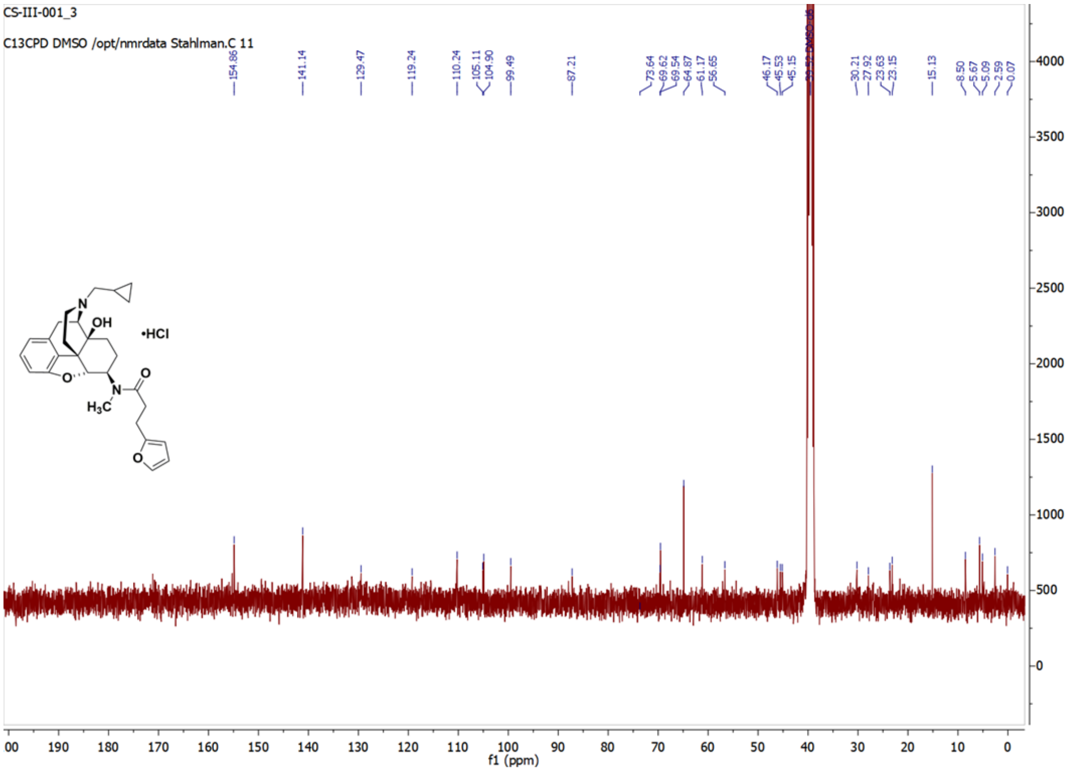

17-Cyclopropylmethyl-4,5*α*-epoxy-6*β*-[3’-(furan-2”-yl)propanamido]-14*β*-hydroxymorphinan hydrochloride **(12)**

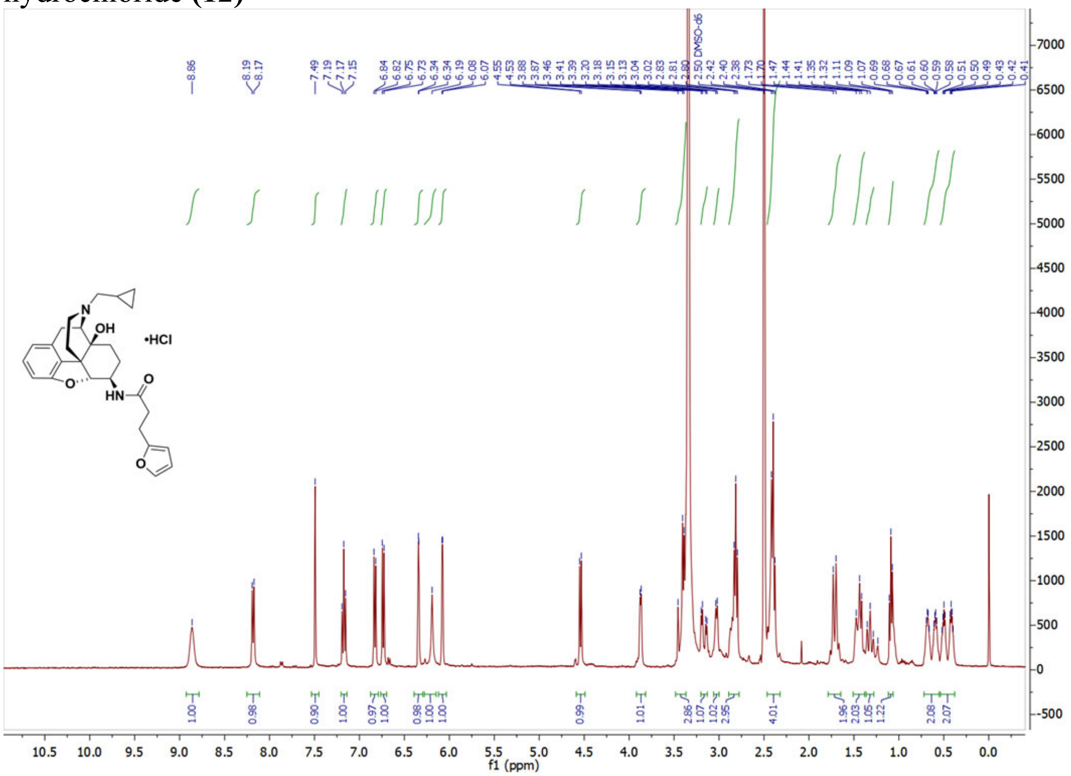

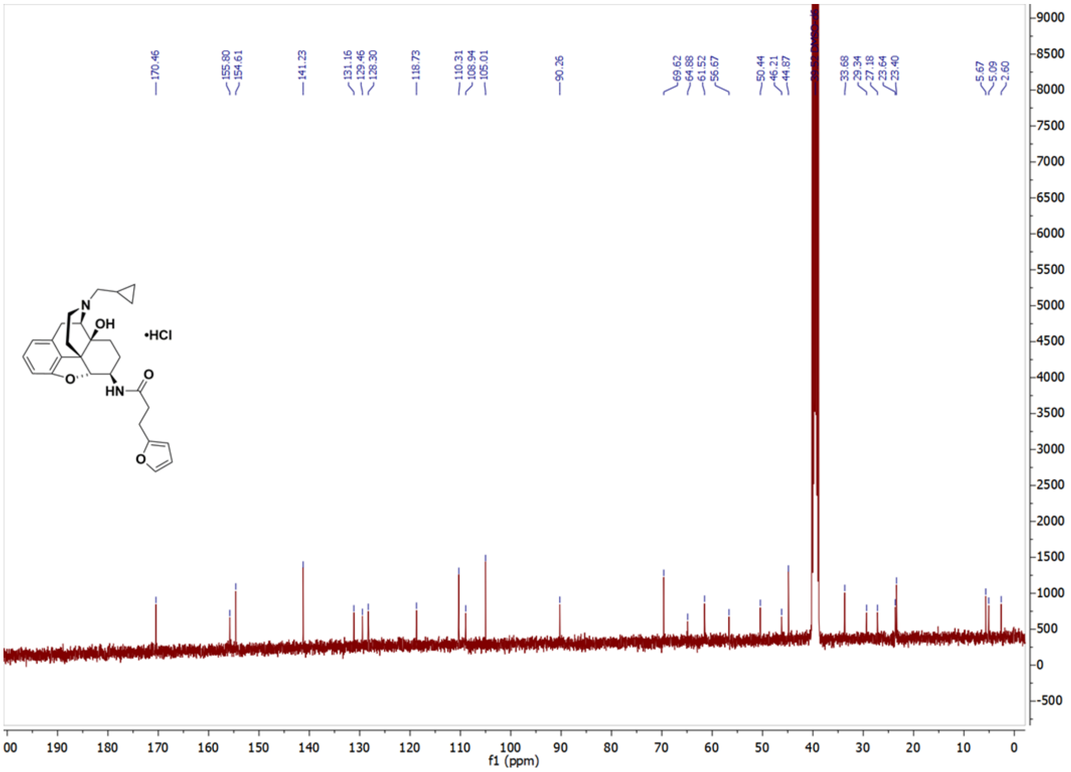

17-Cyclopropylmethyl-3,14β-dihydroxy-4,5α-epoxy-6α-[3'-(furan-2''-yl)-N-methylpropanamido]morphinan hydrochloride **(13)**

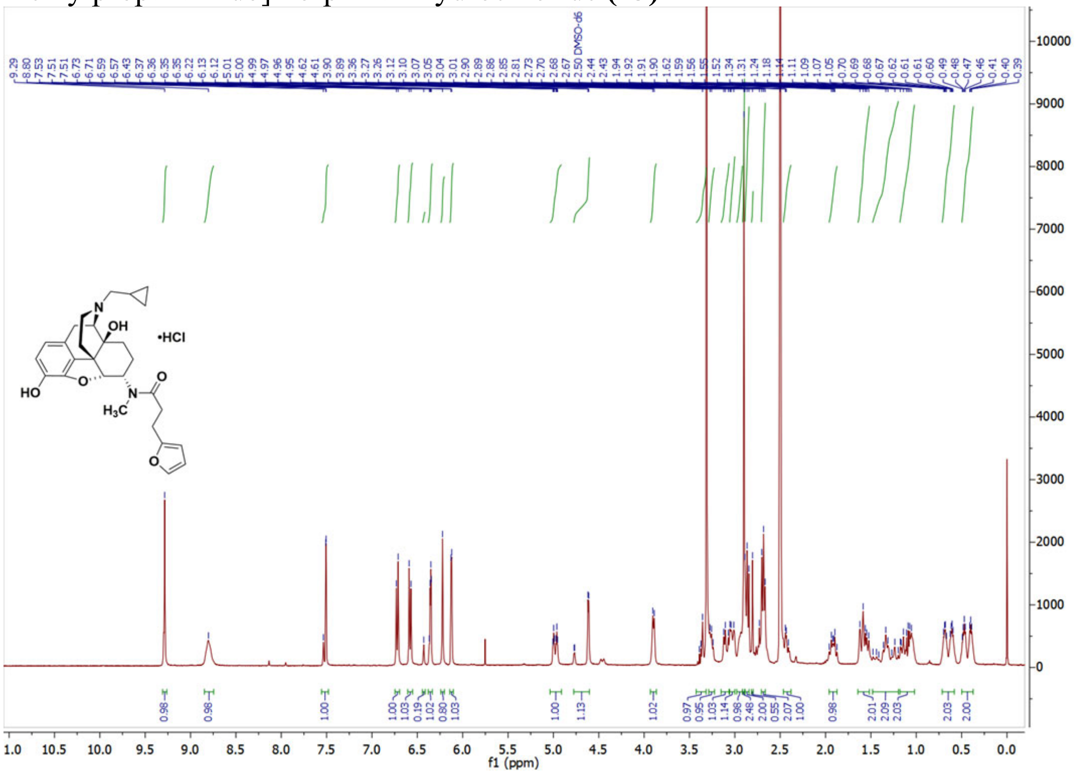

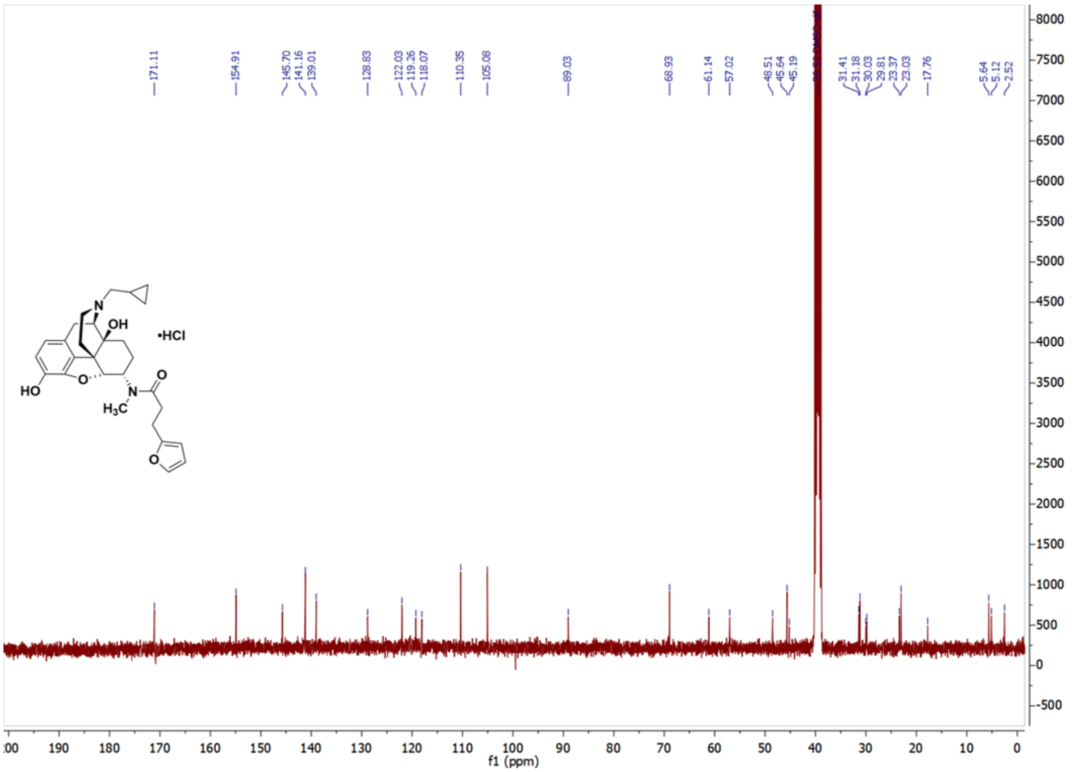

17-Cyclopropylmethyl-3,14β-dihydroxy-4,5α-epoxy-6α-[3'-(furan-2''-yl)propanamido]morphinan hydrochloride **(14)**

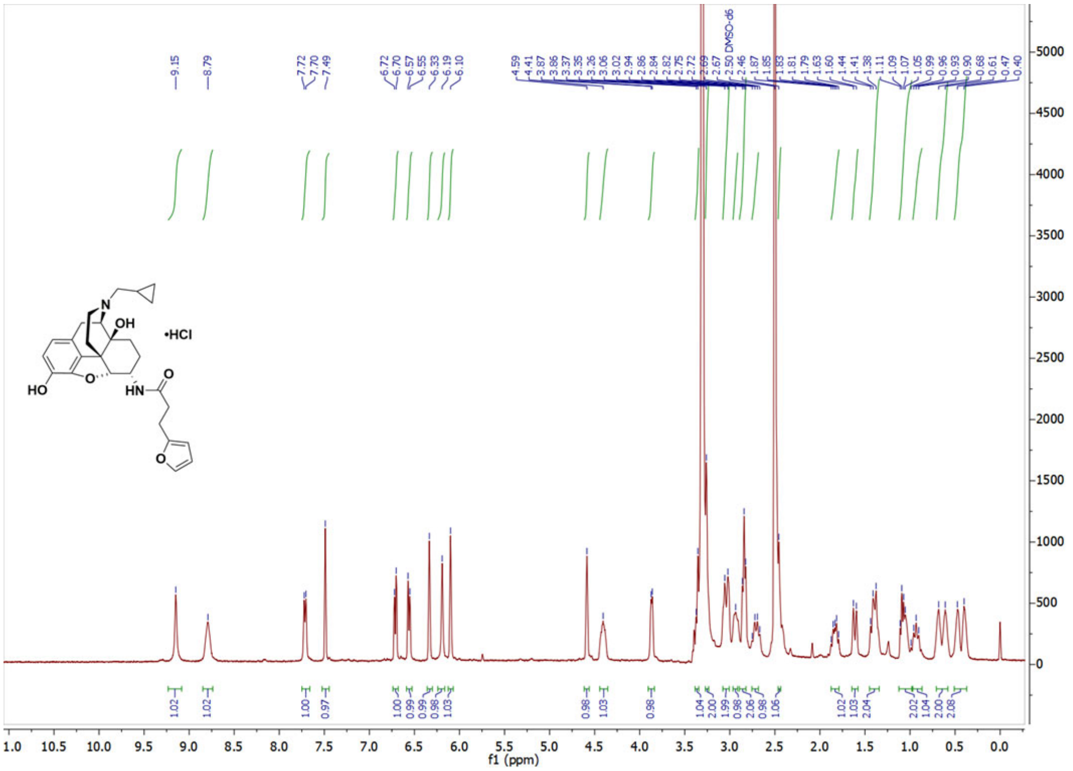

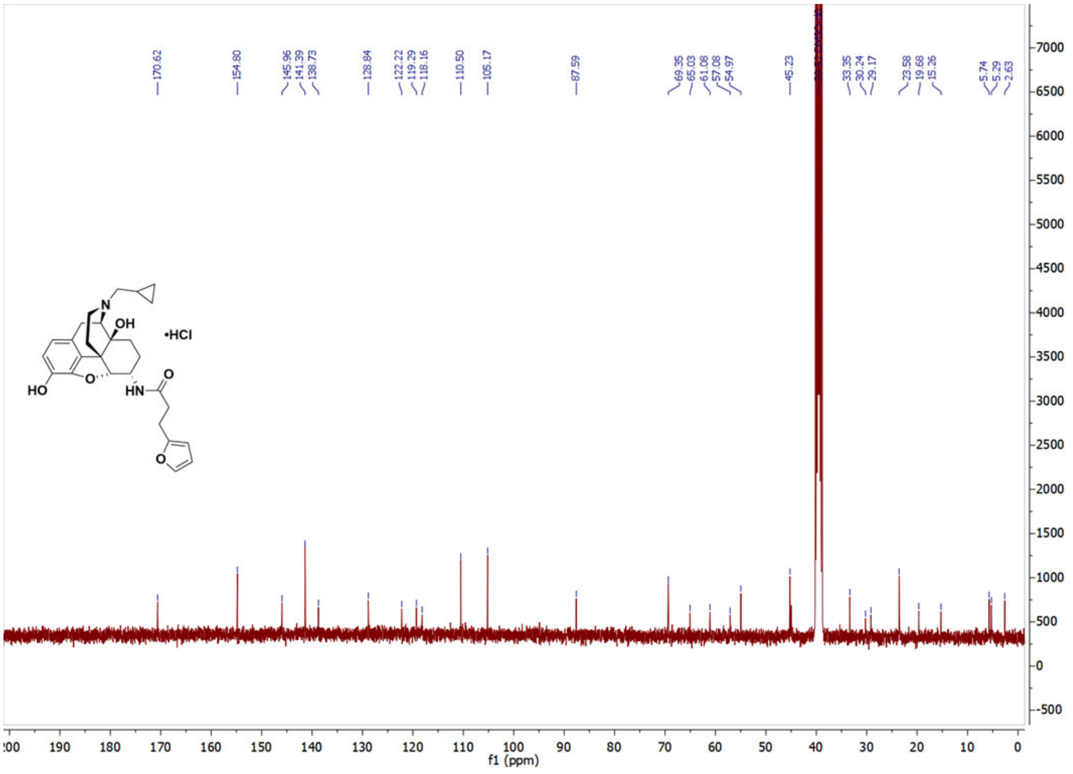

17-Cyclopropylmethyl-3,14β-dihydroxy-4,5α-epoxy-6β-[3'-(furan-2''-yl)-*N*-methylpropanamido]morphinan hydrochloride **(15)**

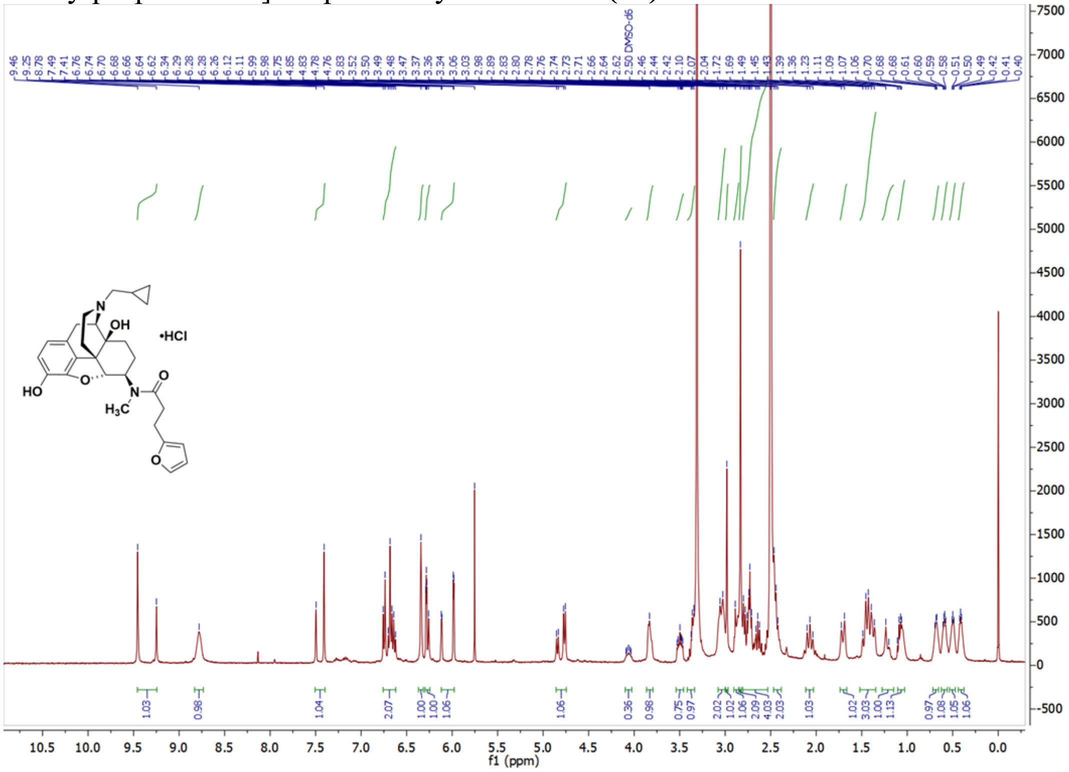

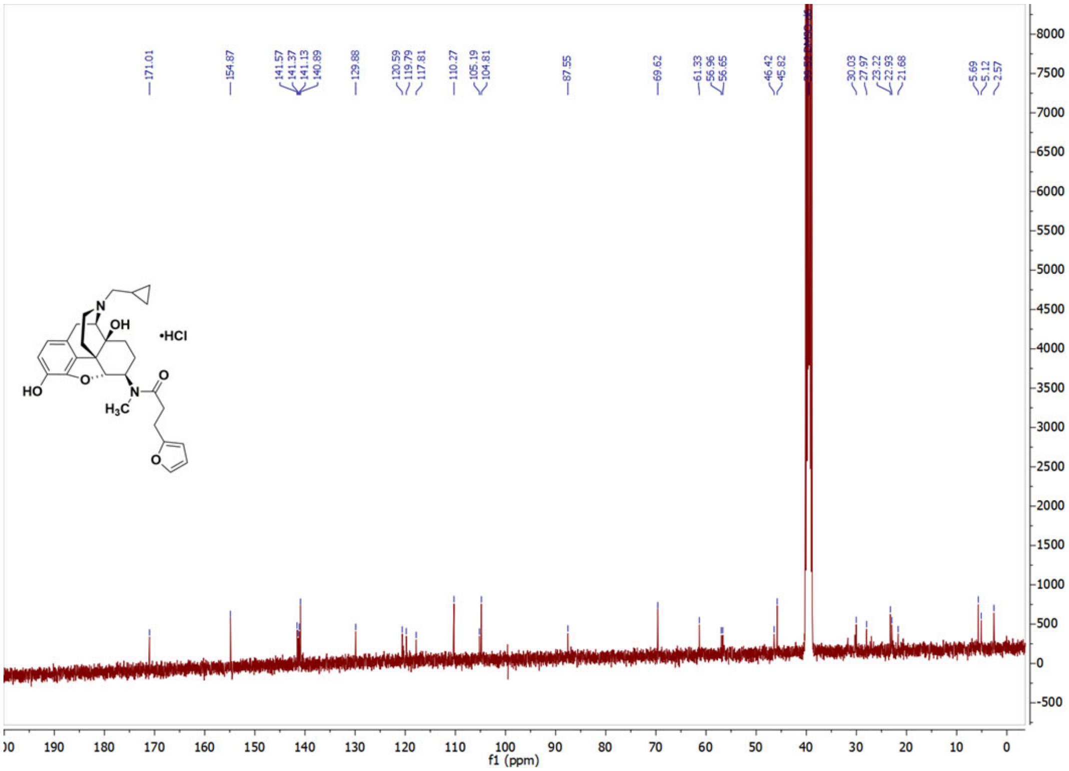

17-Cyclopropylmethyl-3,14*β*-dihydroxy-4,5*α*-epoxy-6*β*-[3’-(furan-2”-yl)propanamido]morphinan hydrochloride **(16)**

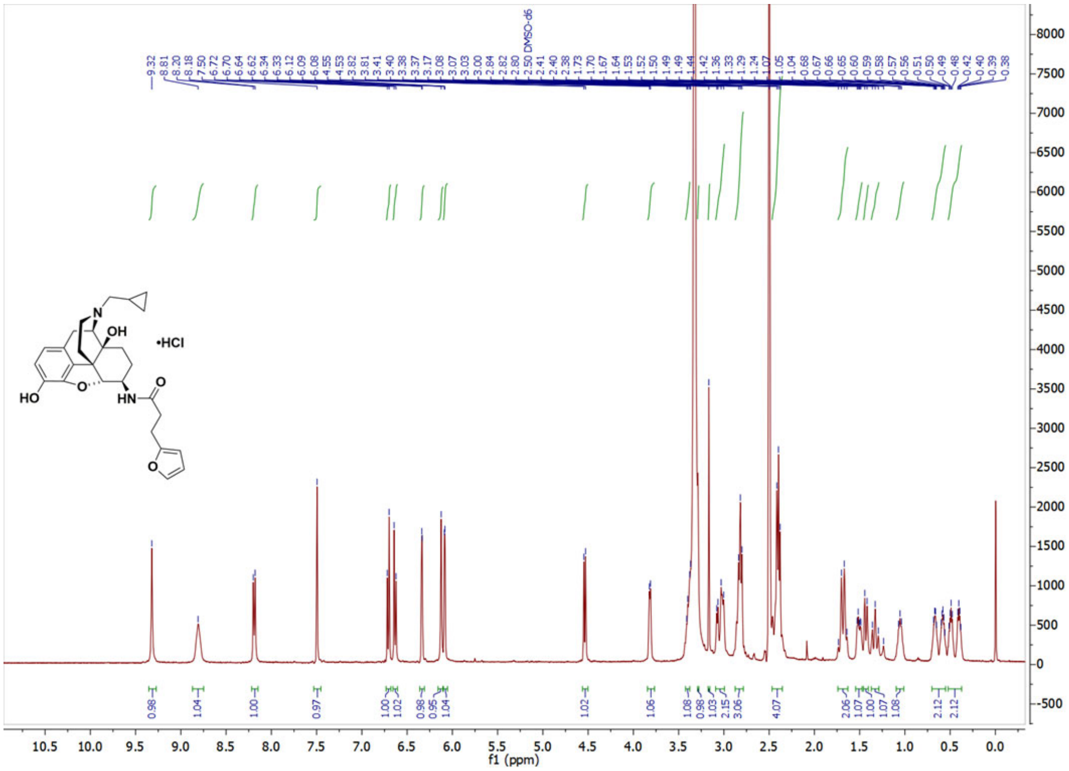

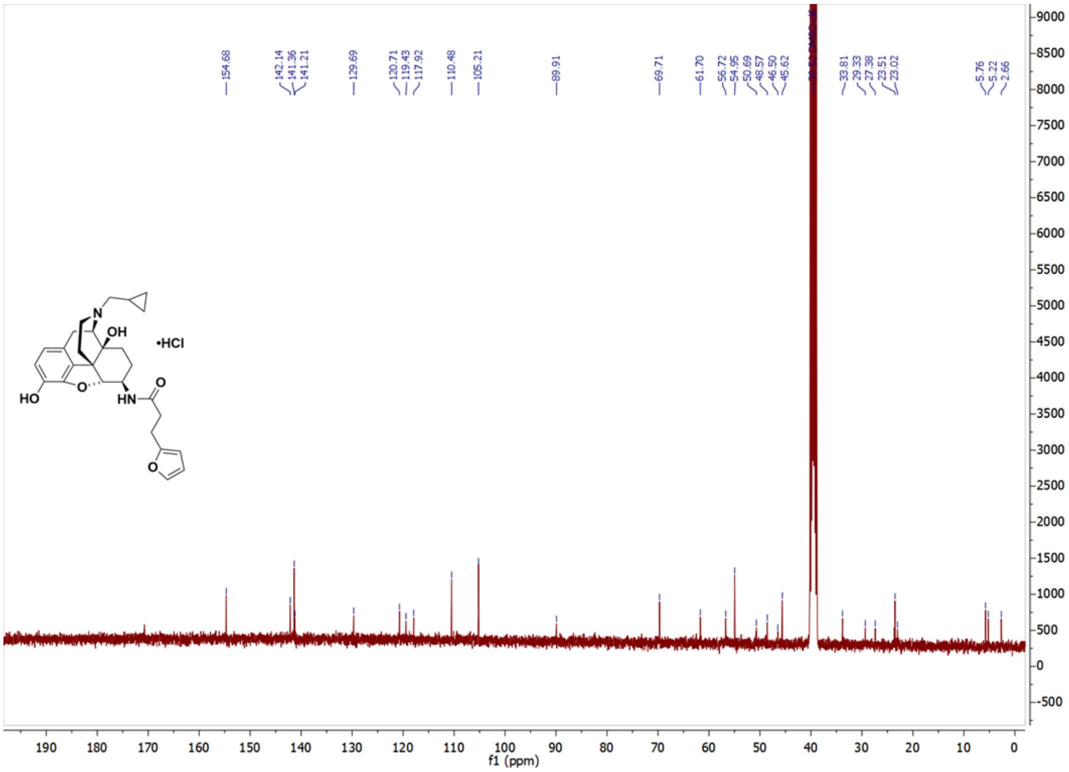

17-Cyclopropylmethyl-4,5*α*-epoxy-6*α*-[(2E)-3’-(furan-2”-yl)*N*-methylprop-2-enamido]-14*β*-hydroxymorphinan hydrochloride **(17)**

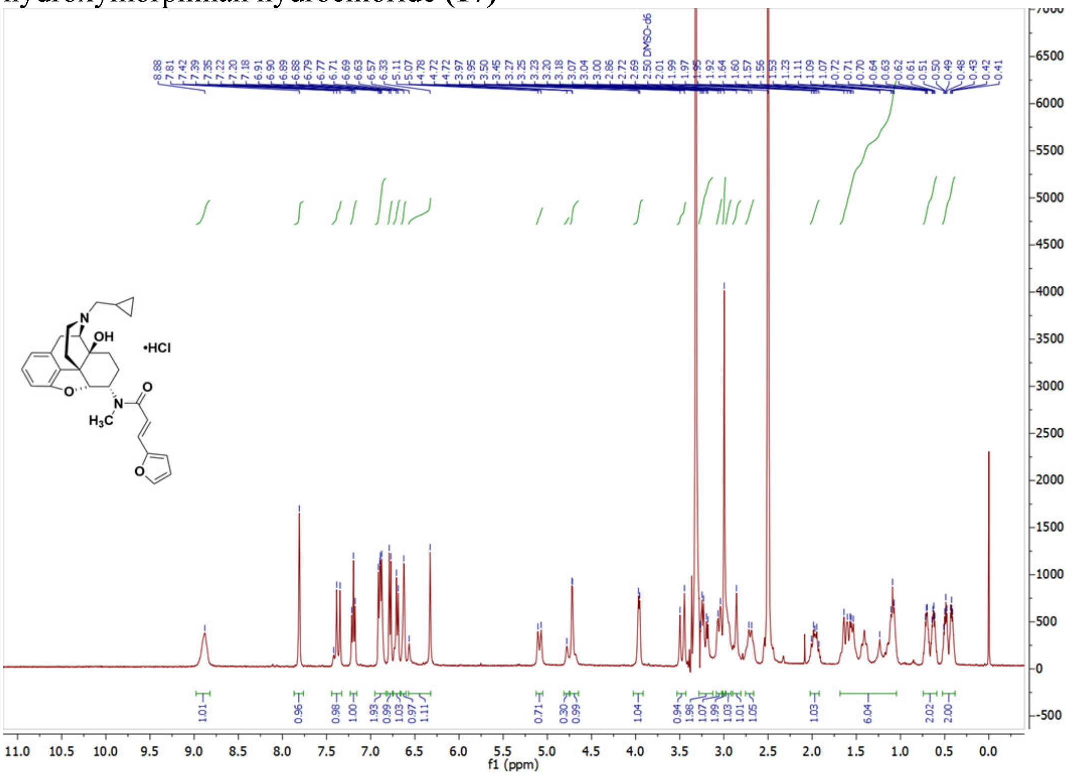

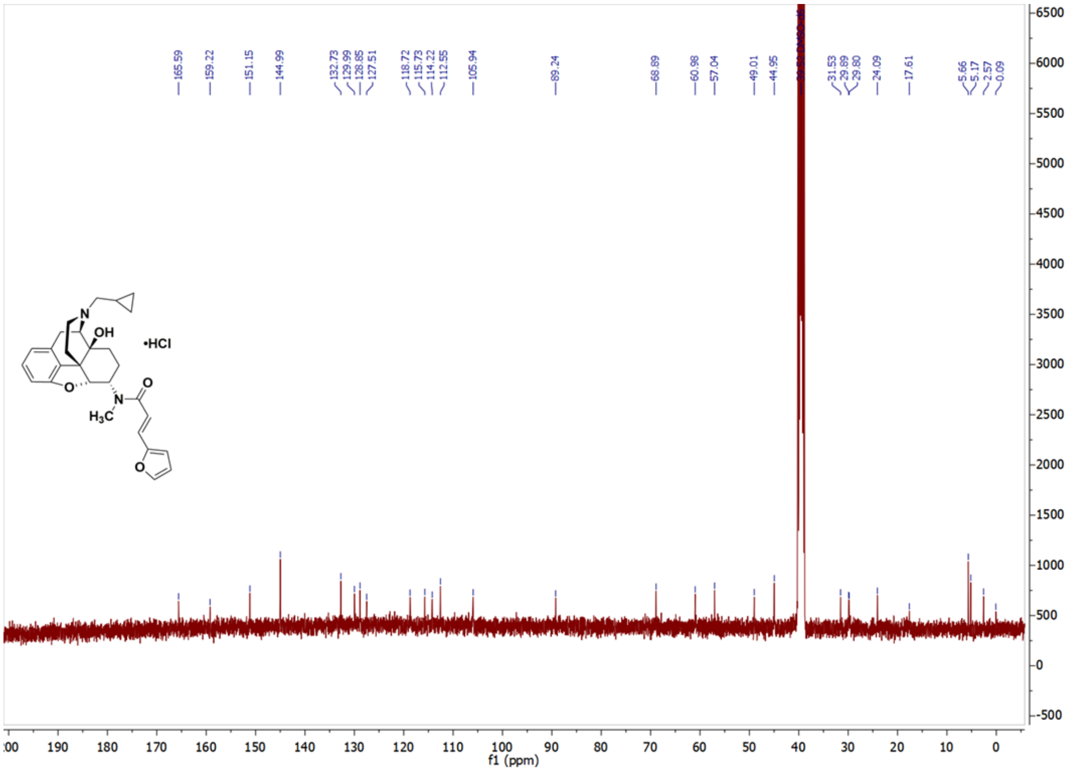

17-Cyclopropylmethyl-4,5α-epoxy-6α-[(2E)-3'-(furan-2''-yl)prop-2-enamido]-14β-hydroxymorphinan hydrochloride **(18)**

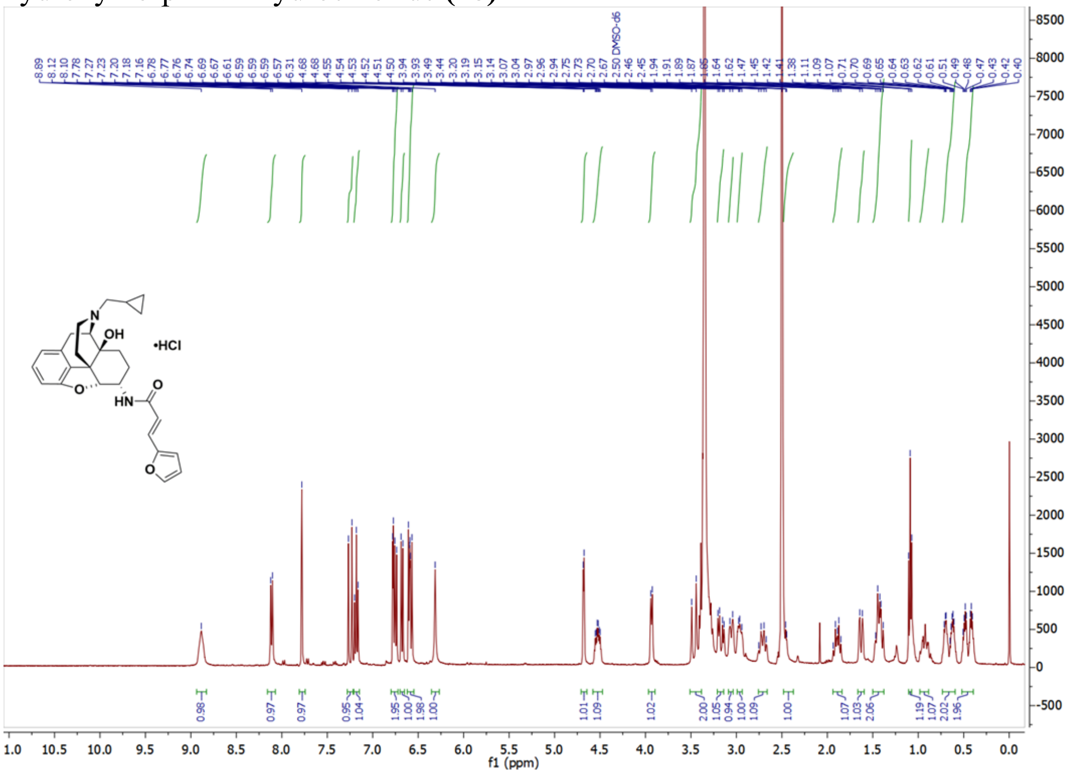

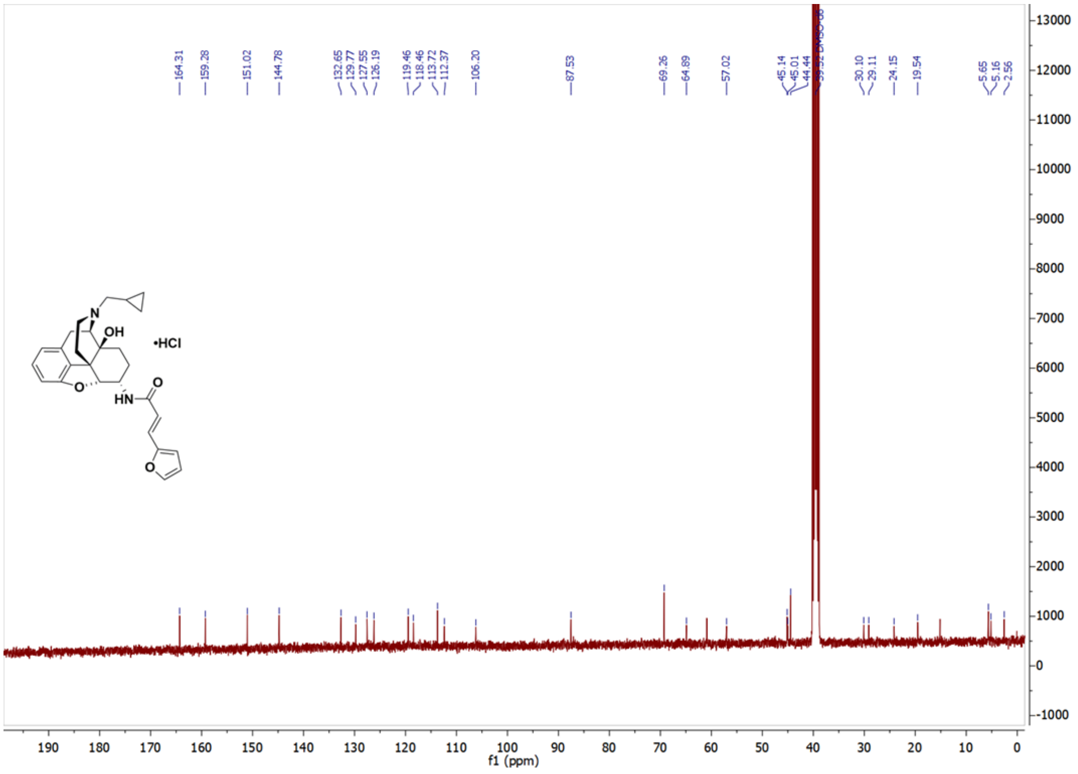

17-Cyclopropylmethyl-4,5α-epoxy-6β-[(2E)-3’-(furan-2”-yl)-*N*-methylprop-2-enamido]-14β-hydroxymorphinan hydrochloride **(19)**

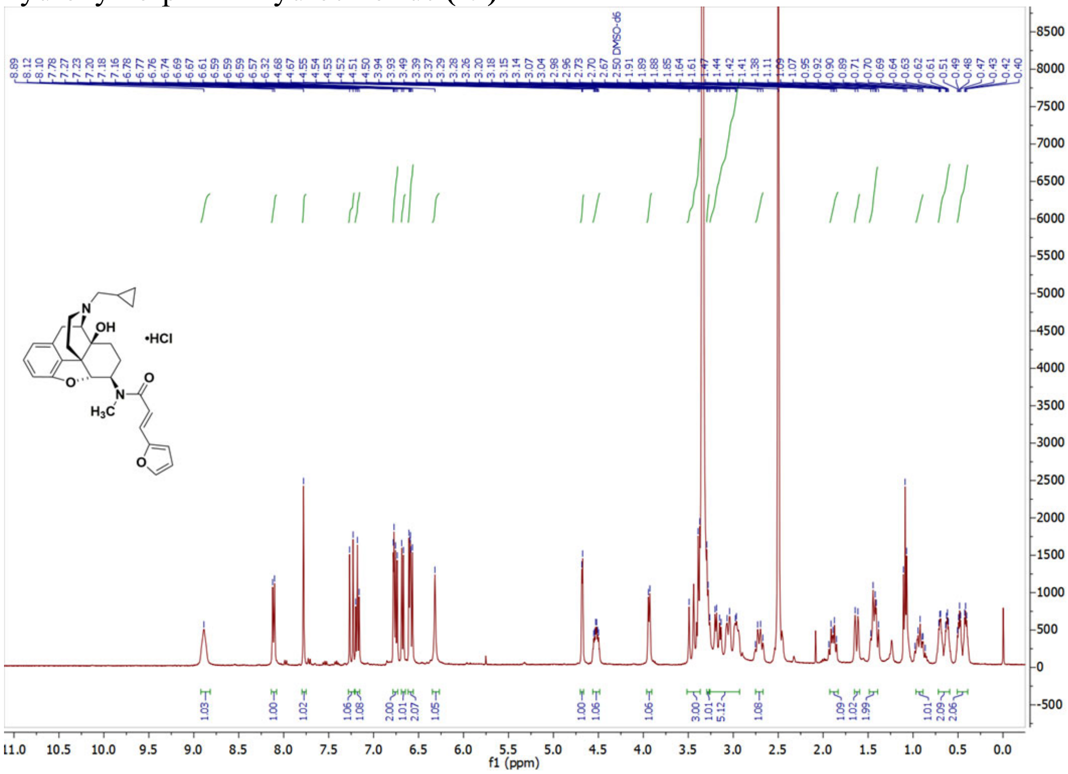

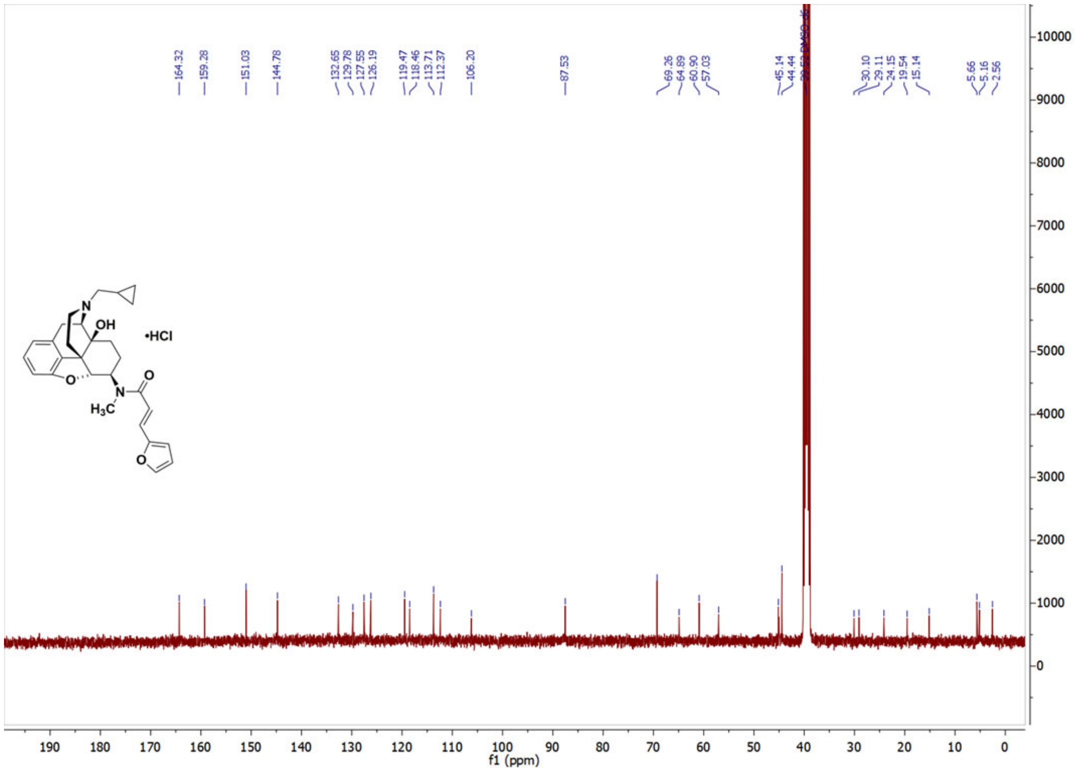

17-Cyclopropylmethyl-4,5α-epoxy-6β-[(2E)-3'-(furan-2''-yl)prop-2-enamido]-14β-hydroxymorphinan hydrochloride **(20)**

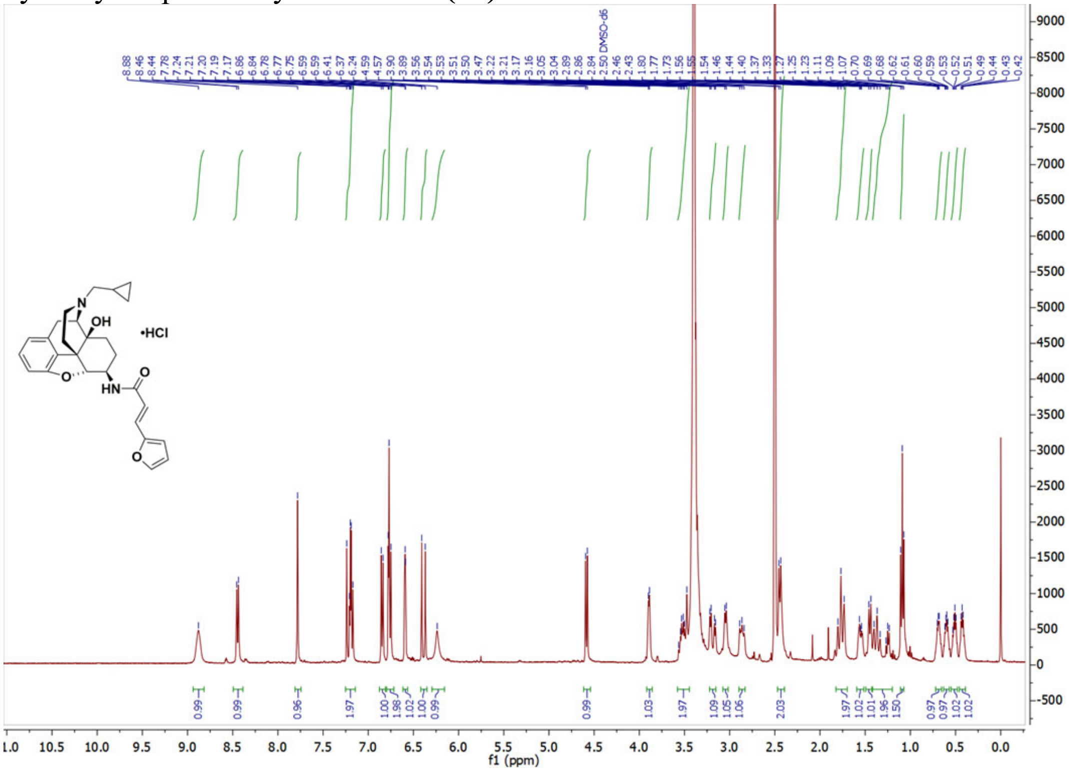

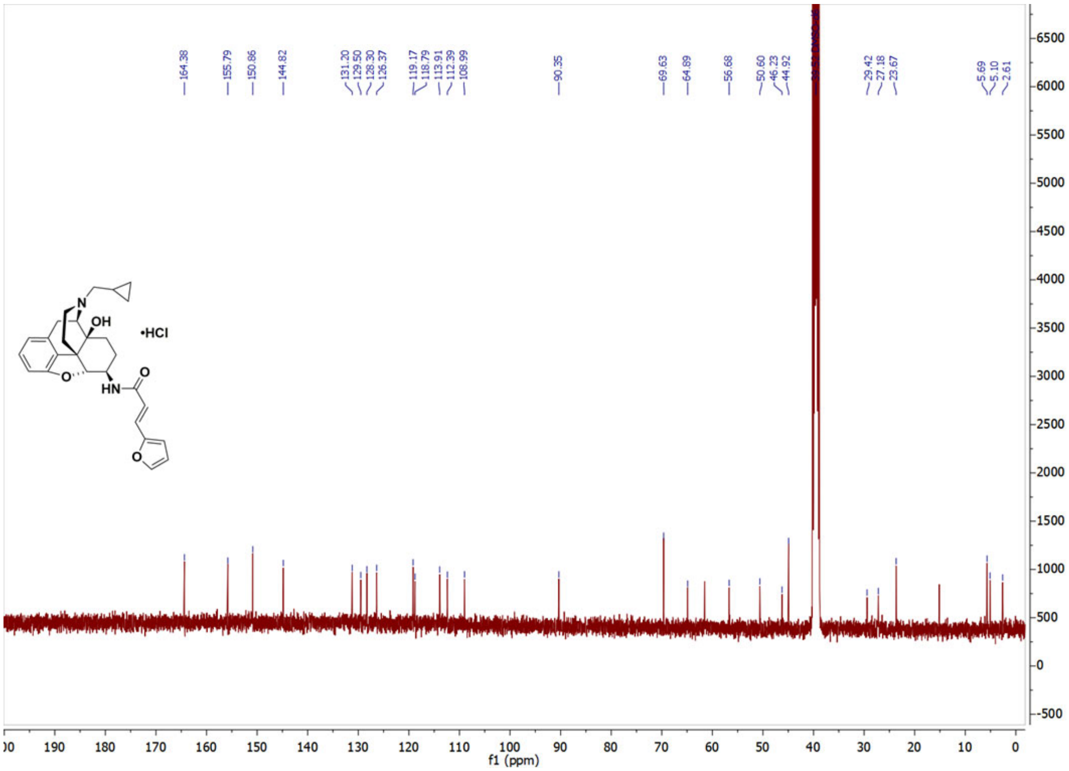

17-Cyclopropylmethyl-3,14β-dihydroxy-4,5α-epoxy-6α-[(2E)-3'-(furan-2''-yl)-N-methylprop-2-enamido]morphinan hydrochloride **(21)**

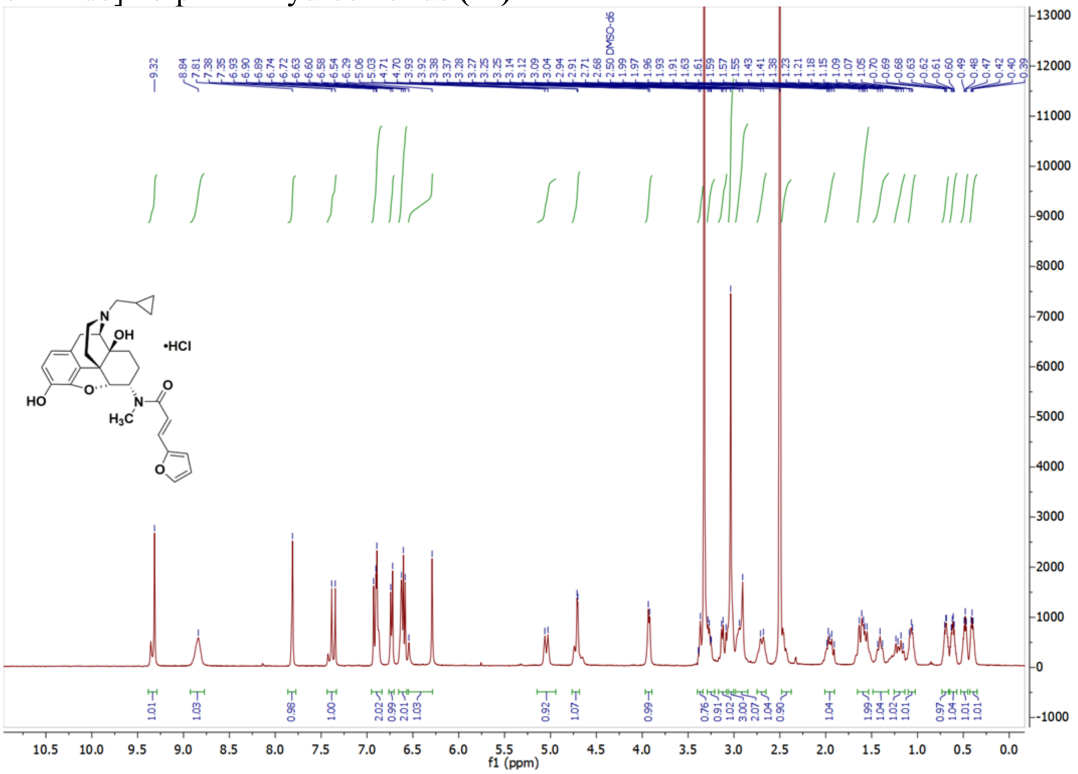

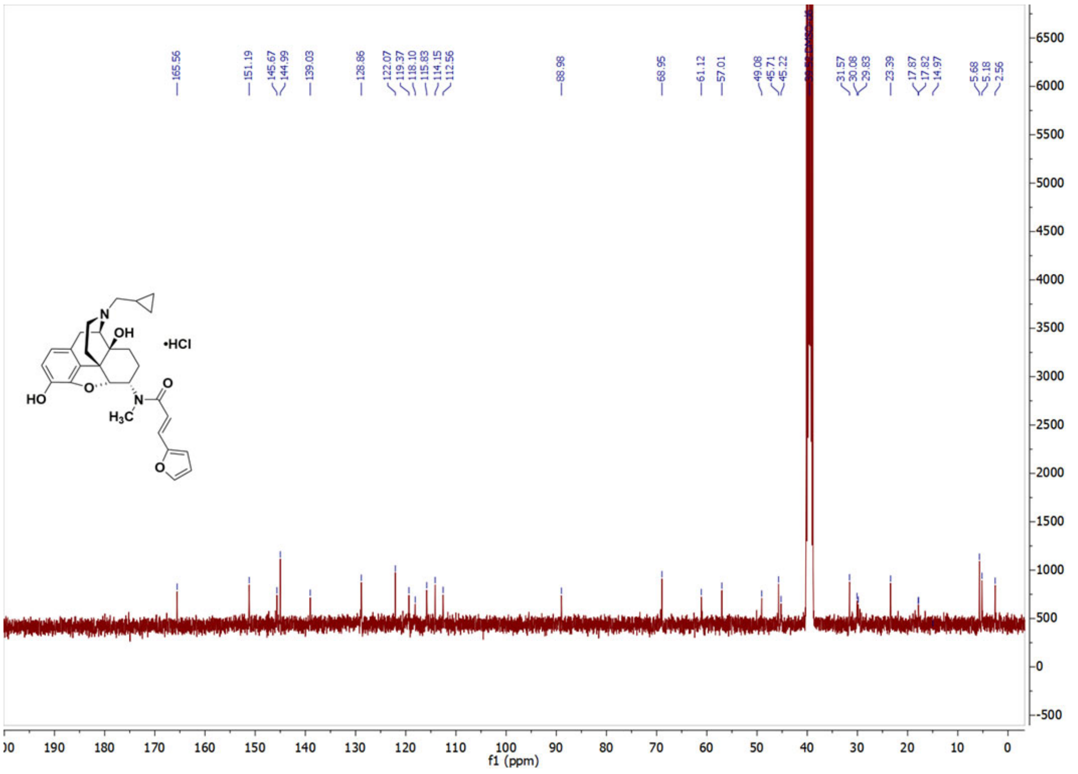

17-Cyclopropylmethyl-3,14*β*-dihydroxy-4,5*α*-epoxy-6*α*-[(2E)-3'-(furan-2''-yl)prop-2-enamido]morphinan hydrochloride **(22)**

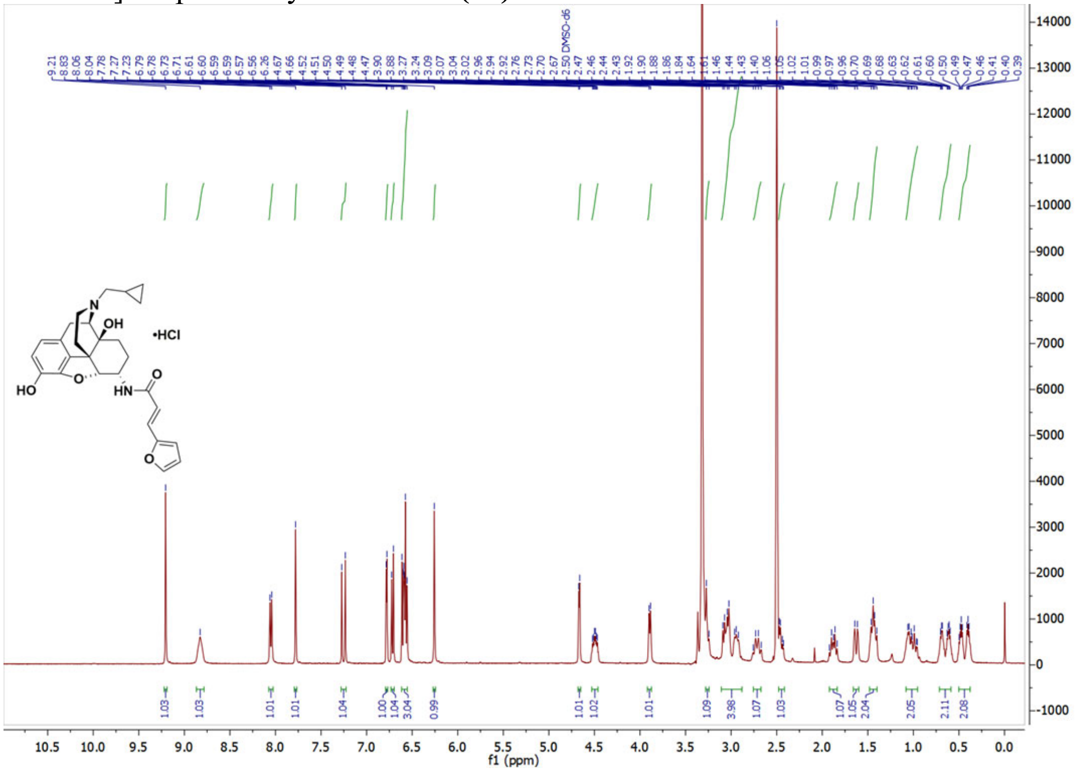

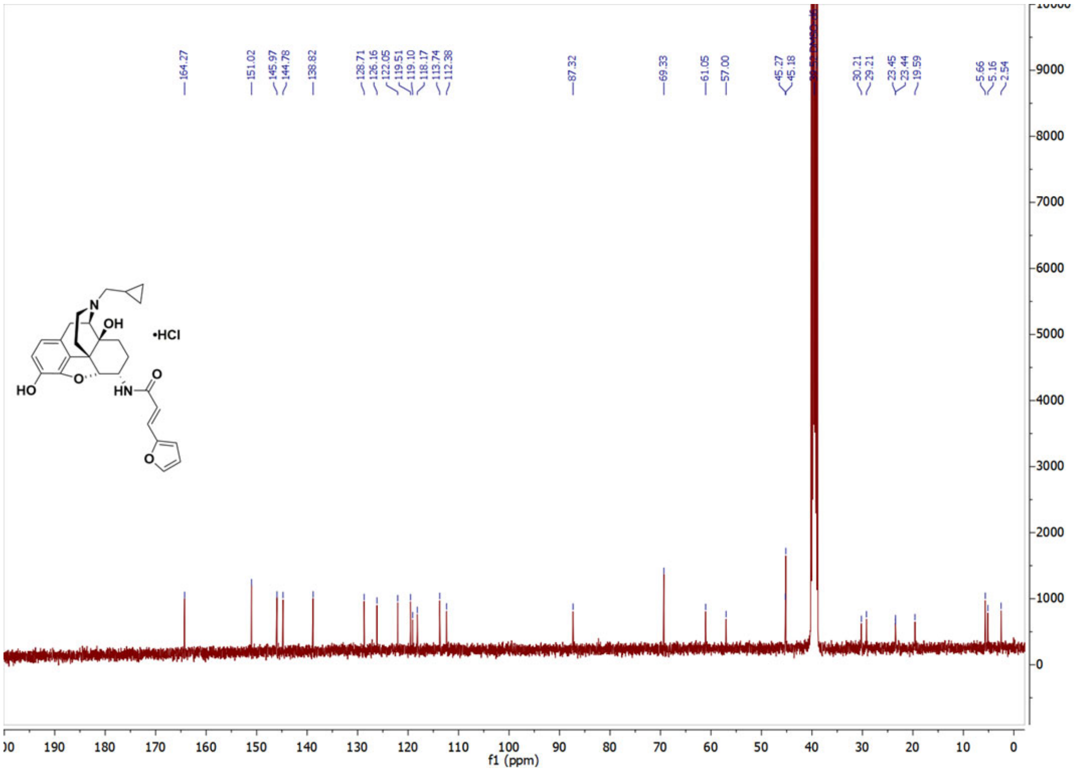

17-Cyclopropylmethyl-3,14β-dihydroxy-4,5α-epoxy-6β-[(2E)-3'-(furan-2"-yl)-*N*-methylprop-2-enamido]morphinan hydrochloride **(23)**

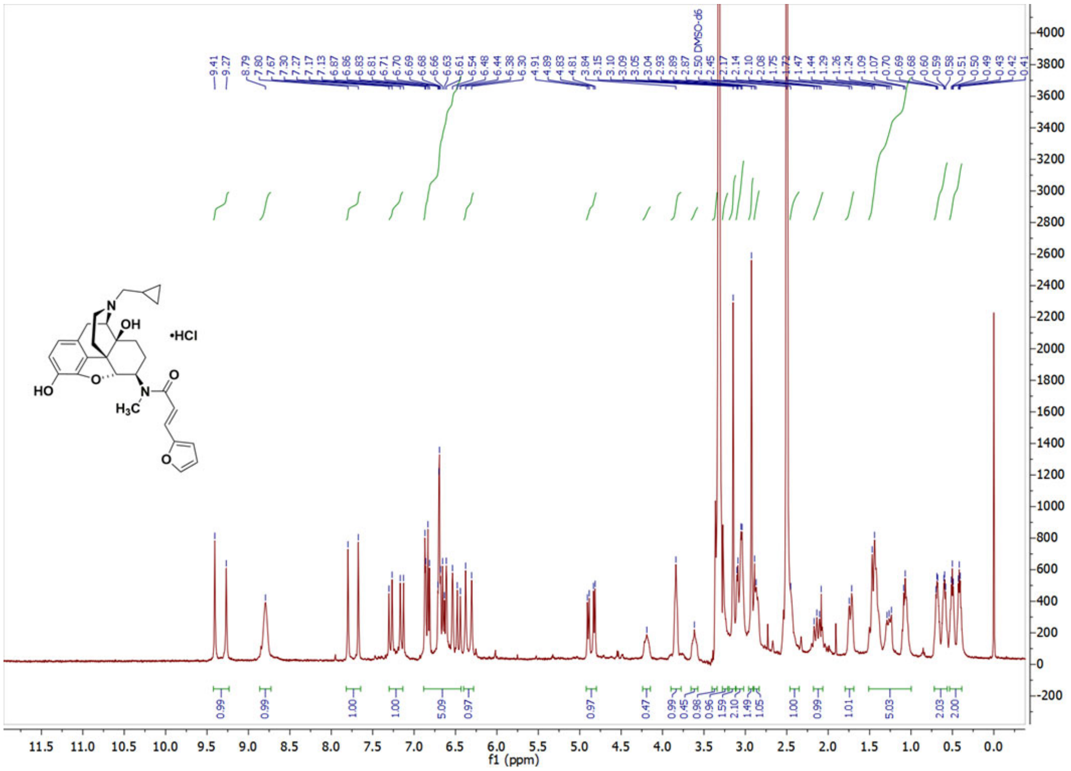

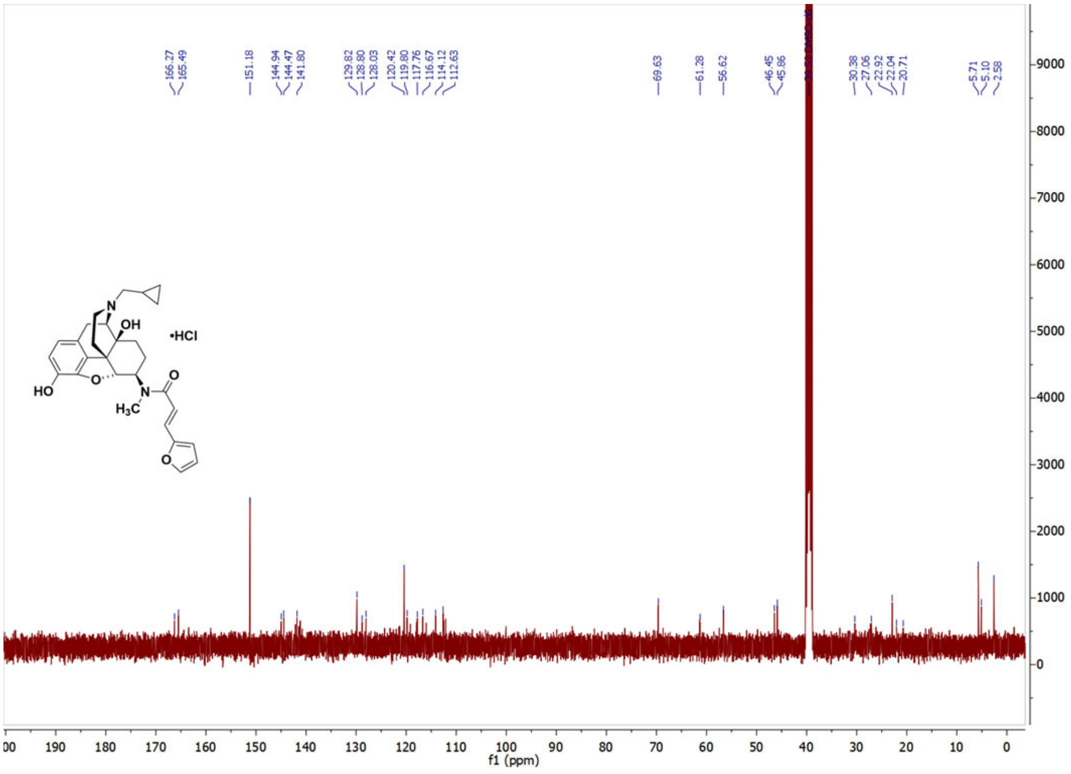

17-Cyclopropylmethyl-3,14*β*-dihydroxy-4,5*α*-epoxy-6*β*-[(2E)-3'-(furan-2"-yl)prop-2-enamido]morphinan hydrochloride **(24)**

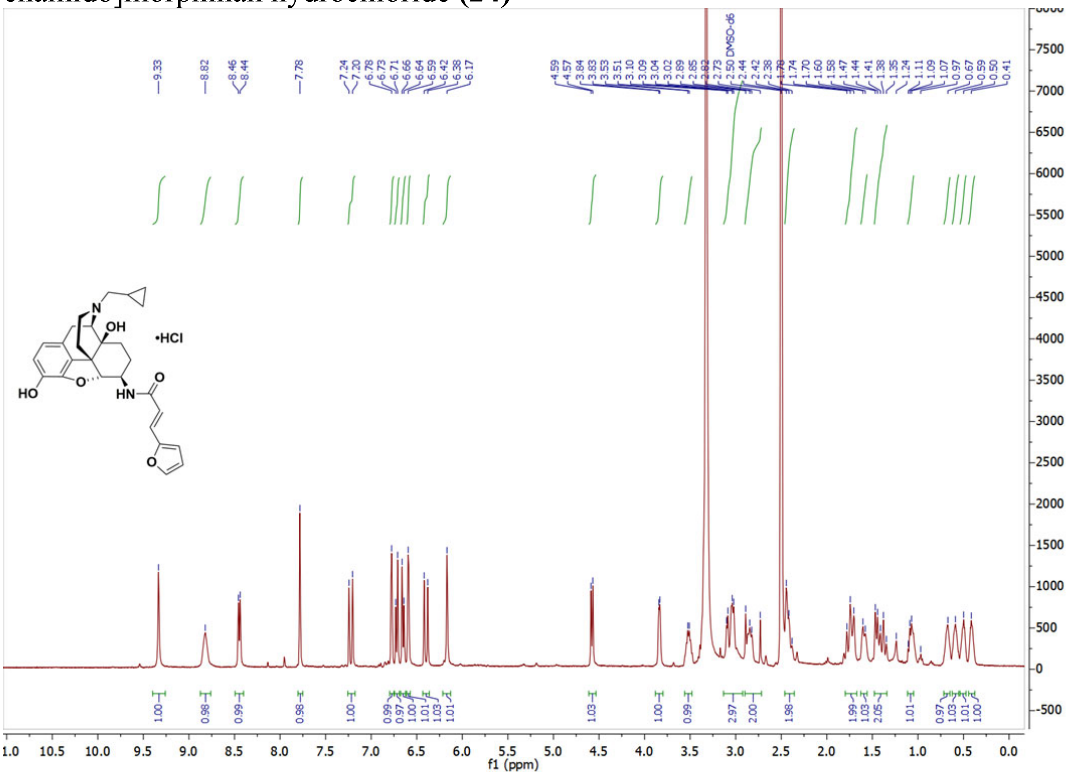

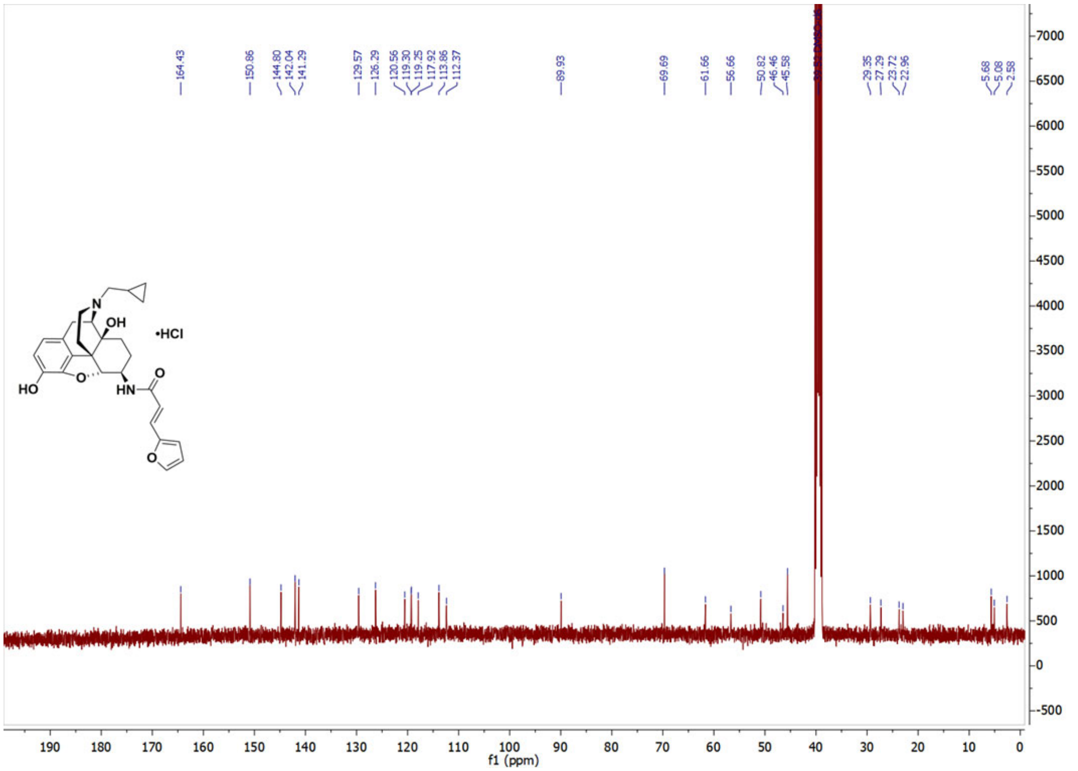

3. **Table S2.** Purity of final compounds.

HPLC System: Waters Arc HPLC
Column: XBridge™ C_{18} 3.5 µm (4.6 x 50 mm)
Injection Volume: 5 µL
Isocratic Mobile Phase:
20% Mobile Phase A – 0.1% Trifluoroacetic acid in water
80% Mobile Phase B – Acetonitrile
Flow Rate: 0.2 mL/min
Single Wavelength: 210 nm
Run time: 10 min

Compound	Retention Time (min)	Purity (%)
1[a]	2.522	95.09
2[a]	2.493	99.25
3[a]	2.493	100.00
4[a]	2.480	97.60
5[b]	2.502	98.17
6[b]	2.522	96.66
7[b]	2.478	97.24
8[b]	2.503	96.65
9[a]	2.522	95.08
10[a]	2.498	97.72
11[a]	2.498	97.05
12[a]	2.485	98.88
13[b]	2.532	99.35
14[b]	2.477	96.17
15[b]	2.497	97.81
16[b]	2.460	99.00
17[a]	2.527	99.72
18[a]	2.495	95.81
19[a]	2.488	99.08
20[a]	2.483	97.35
21[b]	2.515	99.73
22[b]	2.458	99.72
23[b]	2.488	99.00
24[b]	2.460	96.36

[a] 0.25 mg/mL in ACN
[b] 0.125 mg/mL in 20% H_2O/80% ACN

4. HPLC chromatograms of final compounds.

17-Cyclopropylmethyl-4,5*α*-epoxy-6*α*-[3'-(furan-3"-yl)*N*-methylpropanamido]-14*β*-hydroxymorphinan hydrochloride **(1)**

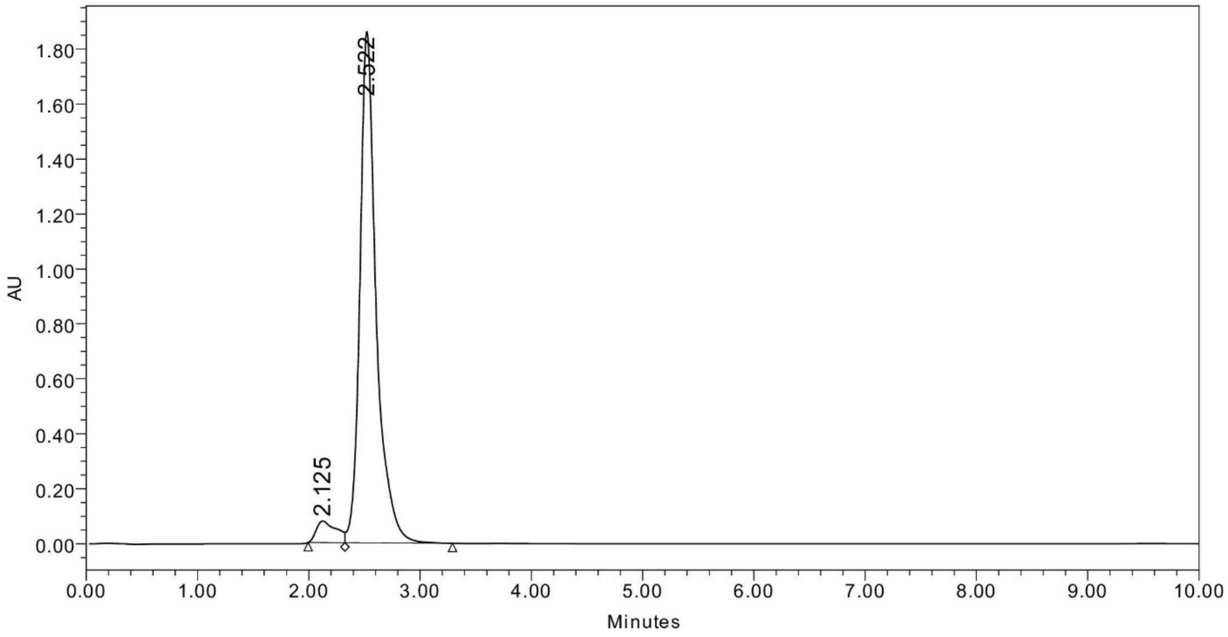

17-Cyclopropylmethyl-4,5*α*-epoxy-6*α*-[3'-(furan-3"-yl)propanamido]-14*β*-hydroxymorphinan hydrochloride **(2)**

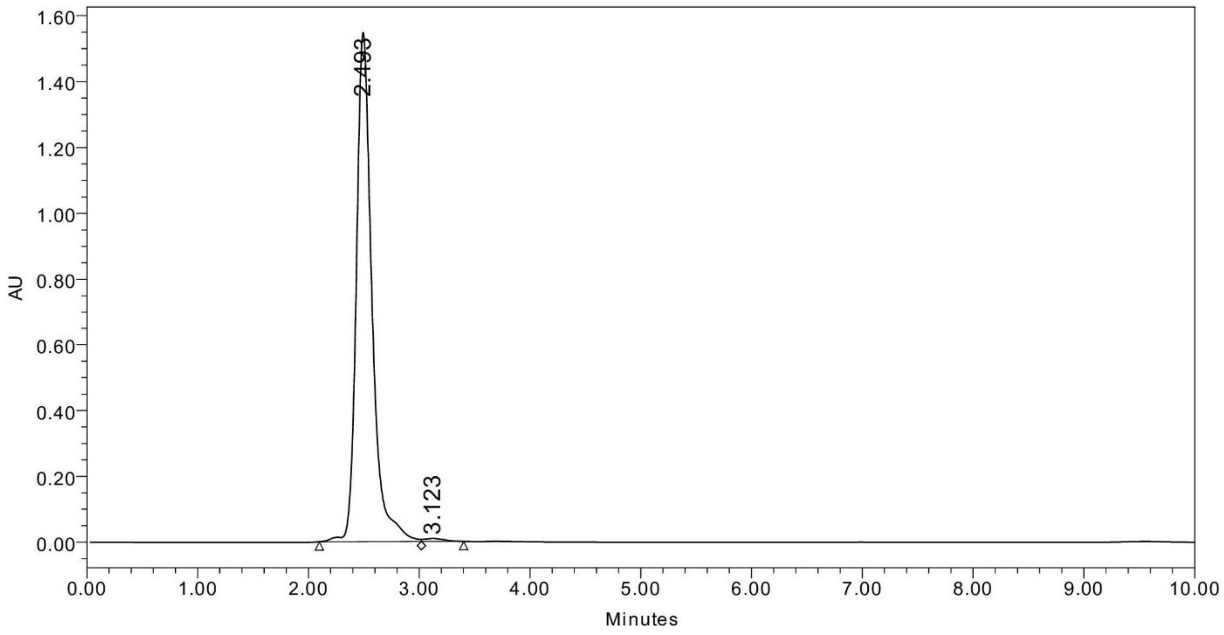

17-Cyclopropylmethyl-4,5α-epoxy-6β-[3'-(furan-3''-yl)-*N*-methylpropanamido]-14β-hydroxymorphinan hydrochloride **(3)**

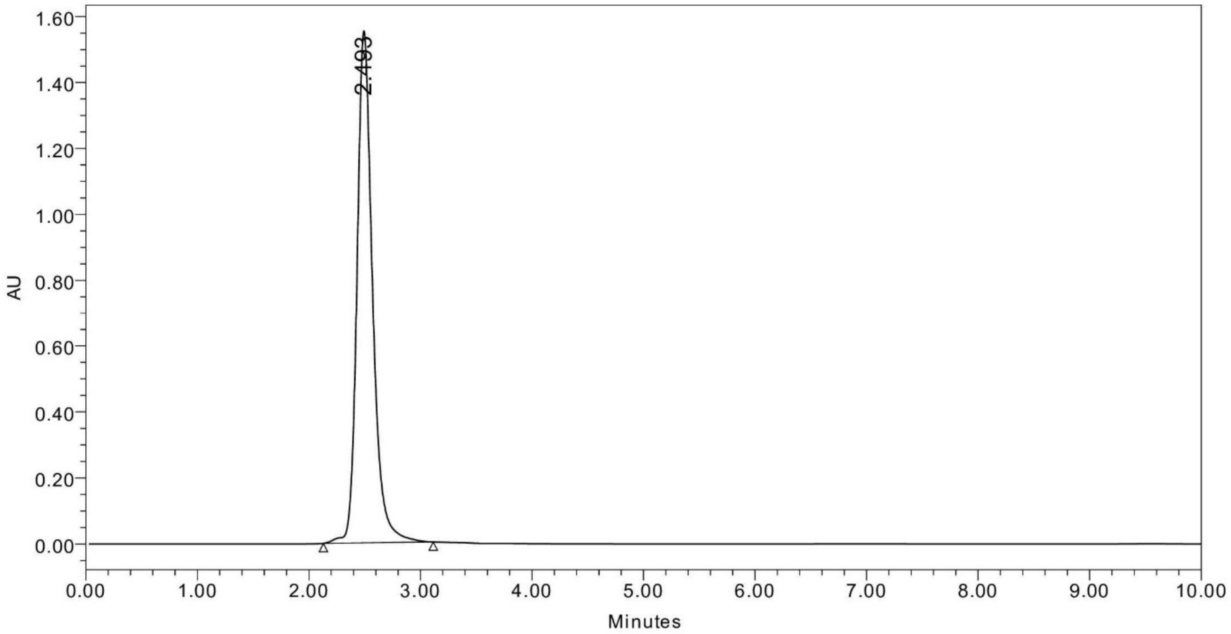

17-Cyclopropylmethyl-4,5α-epoxy-6β-[3'-(furan-3''-yl)propanamido]-14β-hydroxymorphinan hydrochloride **(4)**

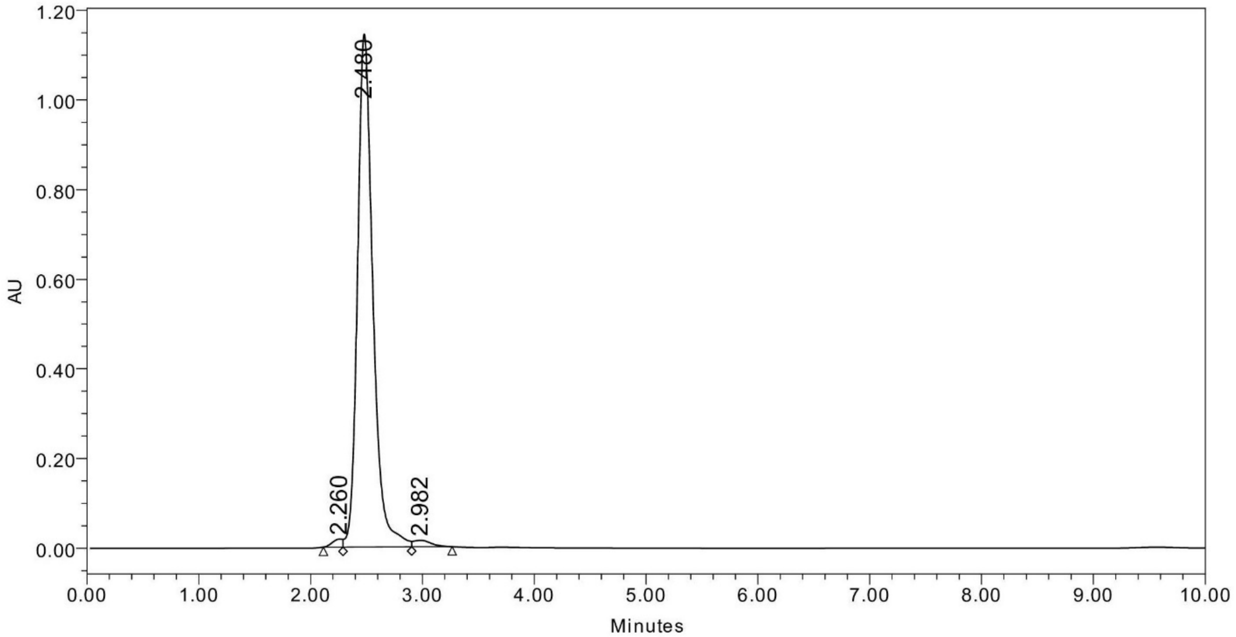

17-Cyclopropylmethyl-3,14*β*-dihydroxy-4,5*α*-epoxy-6*α*-[3'-(furan-3''-yl)-N-methylpropanamido]morphinan hydrochloride **(5)**

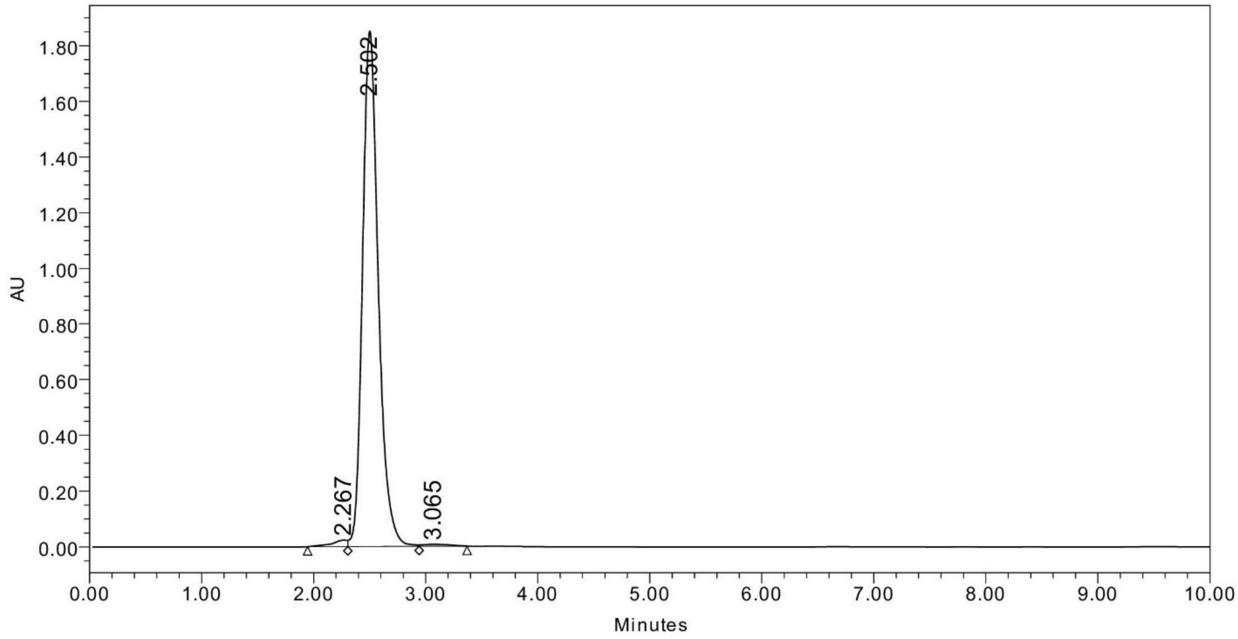

17-Cyclopropylmethyl-3,14*β*-dihydroxy-4,5*α*-epoxy-6*α*-[3'-(furan-3''-yl)propanamido]morphinan hydrochloride **(6)**

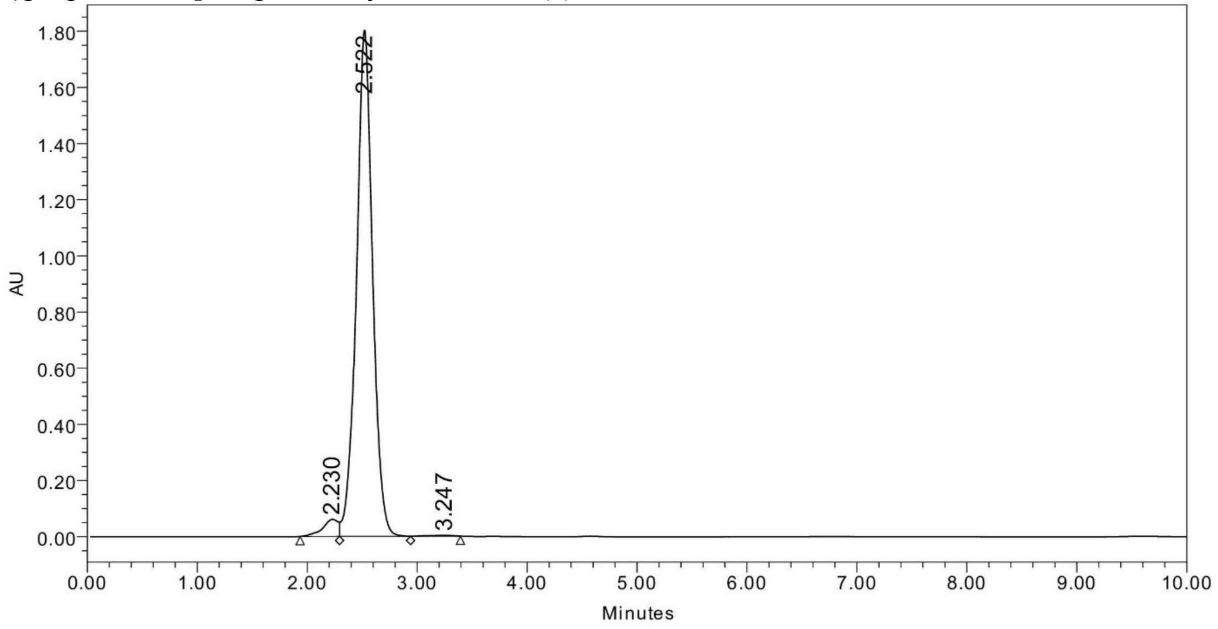

17-Cyclopropylmethyl-3,14β-dihydroxy-4,5α-epoxy-6β-[3'-(furan-3''-yl)-*N*-methylpropanamido]morphinan hydrochloride **(7)**

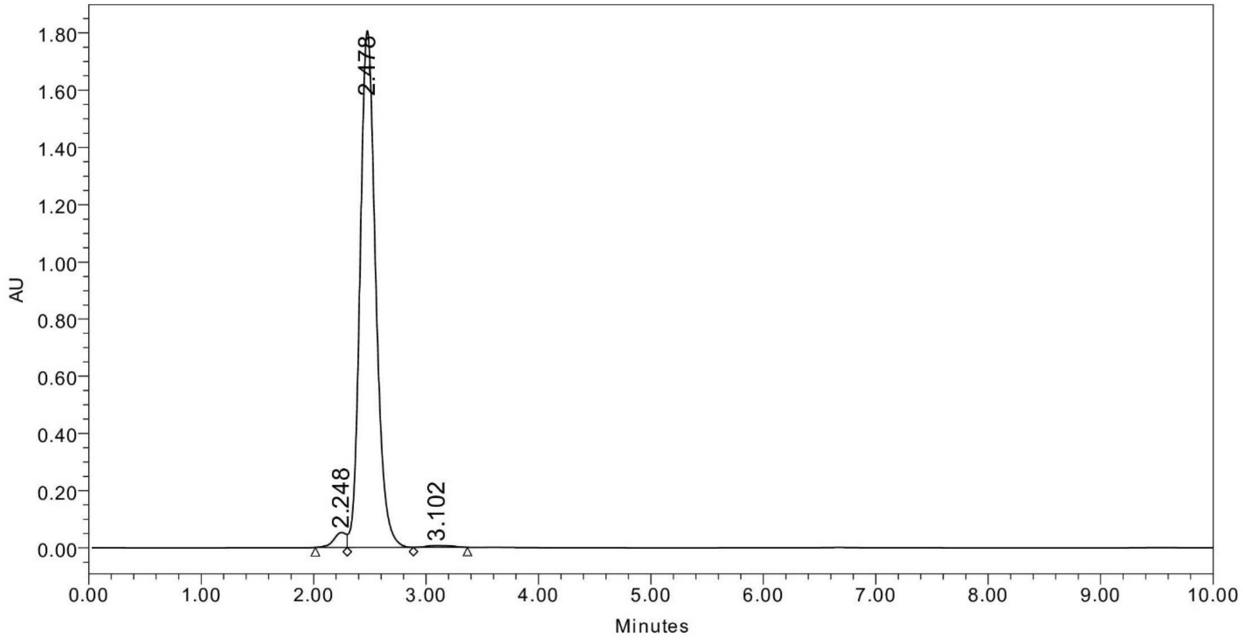

17-Cyclopropylmethyl-3,14β-dihydroxy-4,5α-epoxy-6β-[3'-(furan-3''-yl)propanamido]morphinan hydrochloride **(8)**

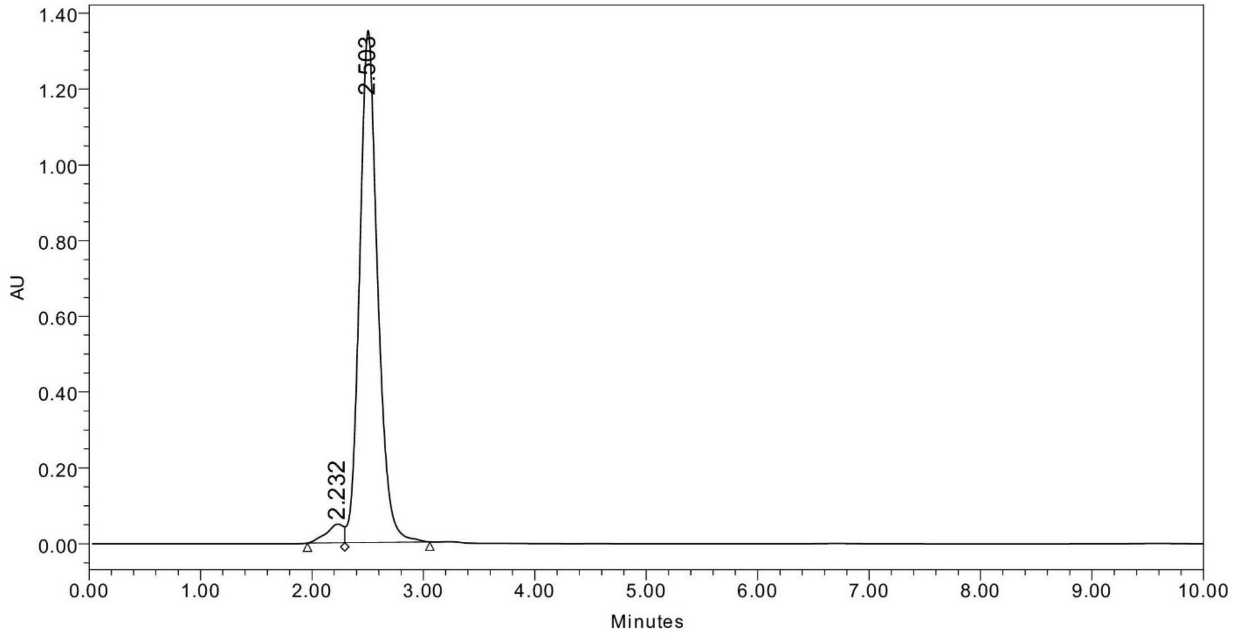

17-Cyclopropylmethyl-4,5α-epoxy-6α-[3'-(furan-2"-yl)*N*-methylpropanamido]-14β-hydroxymorphinan hydrochloride **(9)**

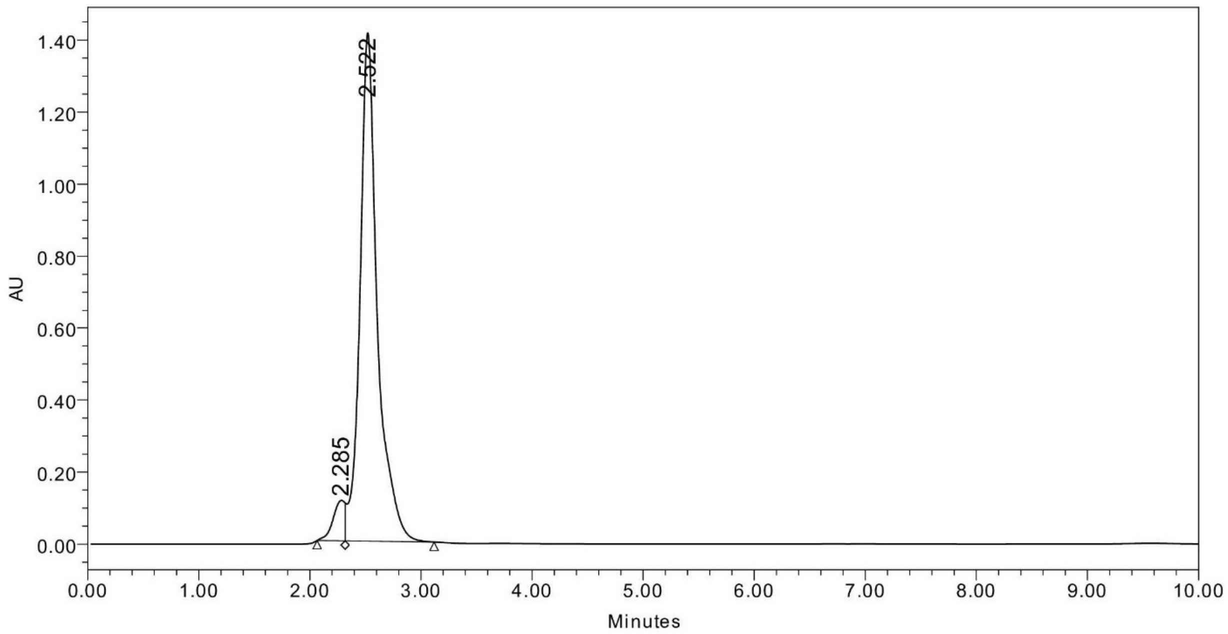

17-Cyclopropylmethyl-4,5α-epoxy-6α-[3'-(furan-2"-yl)propanamido]-14β-hydroxymorphinan hydrochloride **(10)**

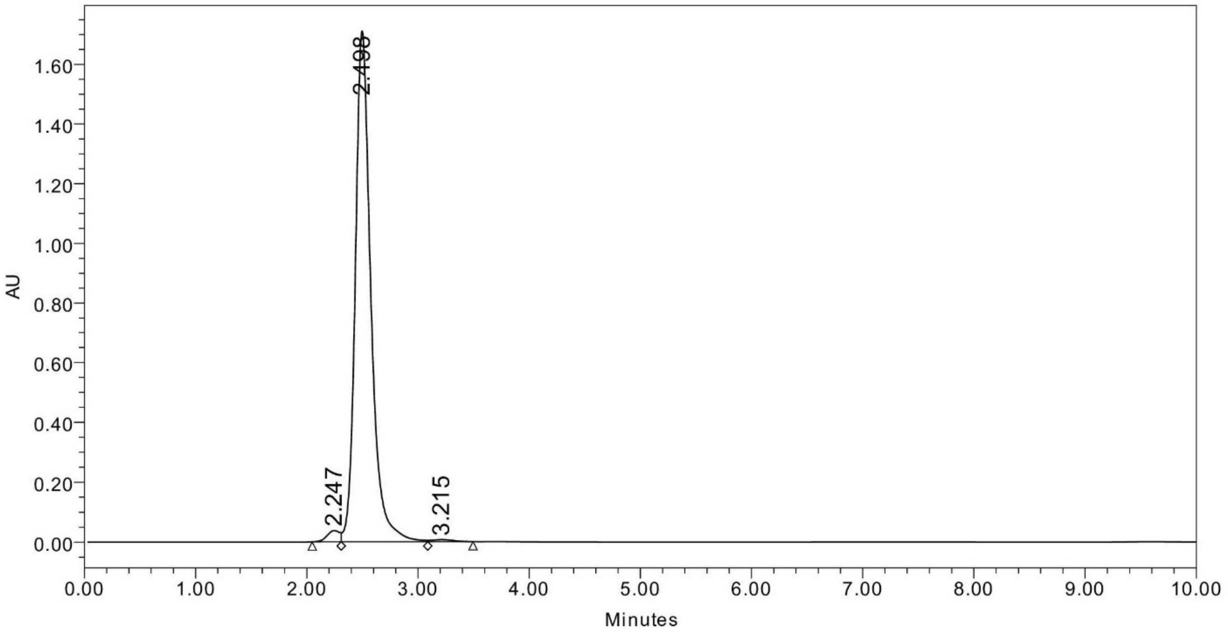

17-Cyclopropylmethyl-4,5α-epoxy-6β-[3'-(furan-2"-yl)-*N*-methylpropanamido]-14β-hydroxymorphinan hydrochloride **(11)**

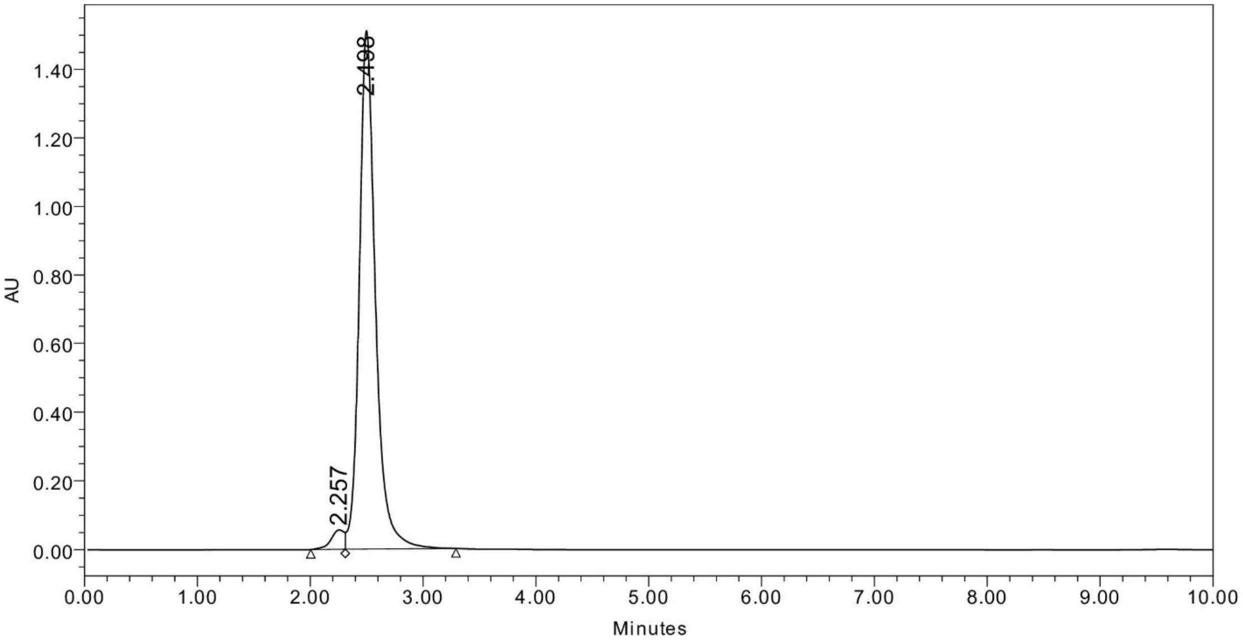

17-Cyclopropylmethyl-4,5α-epoxy-6β-[3'-(furan-2"-yl)propanamido]-14β-hydroxymorphinan hydrochloride **(12)**

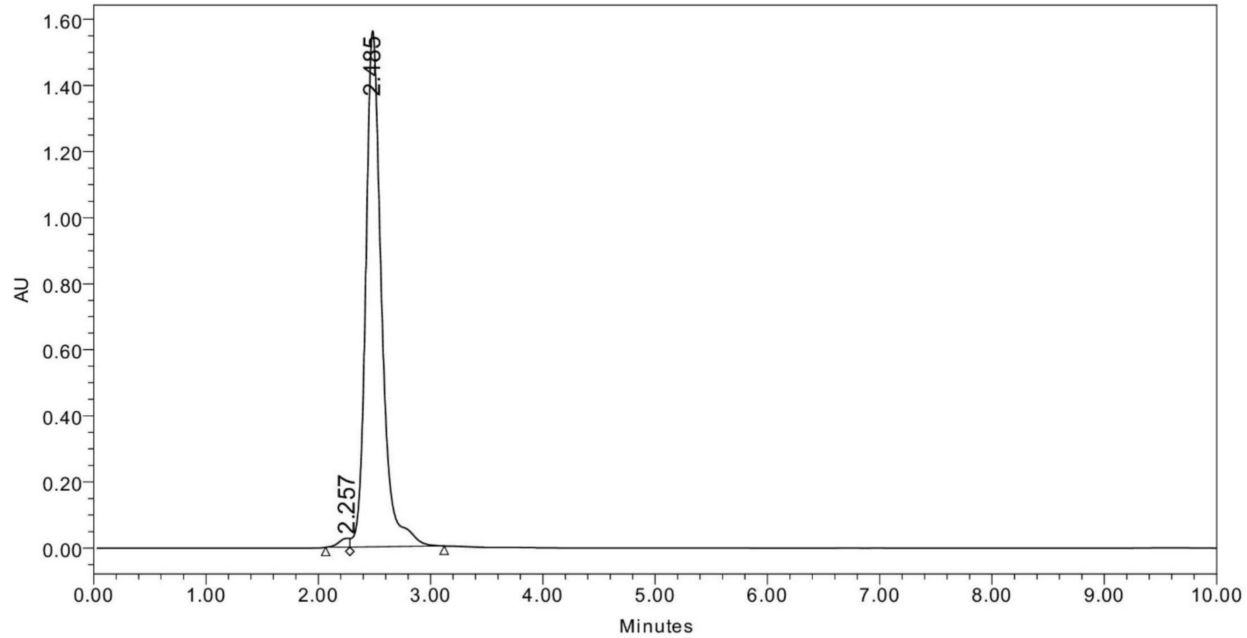

17-Cyclopropylmethyl-3,14*β*-dihydroxy-4,5*α*-epoxy-6*α*-[3'-(furan-2"-yl)-N-methylpropanamido]morphinan hydrochloride **(13)**

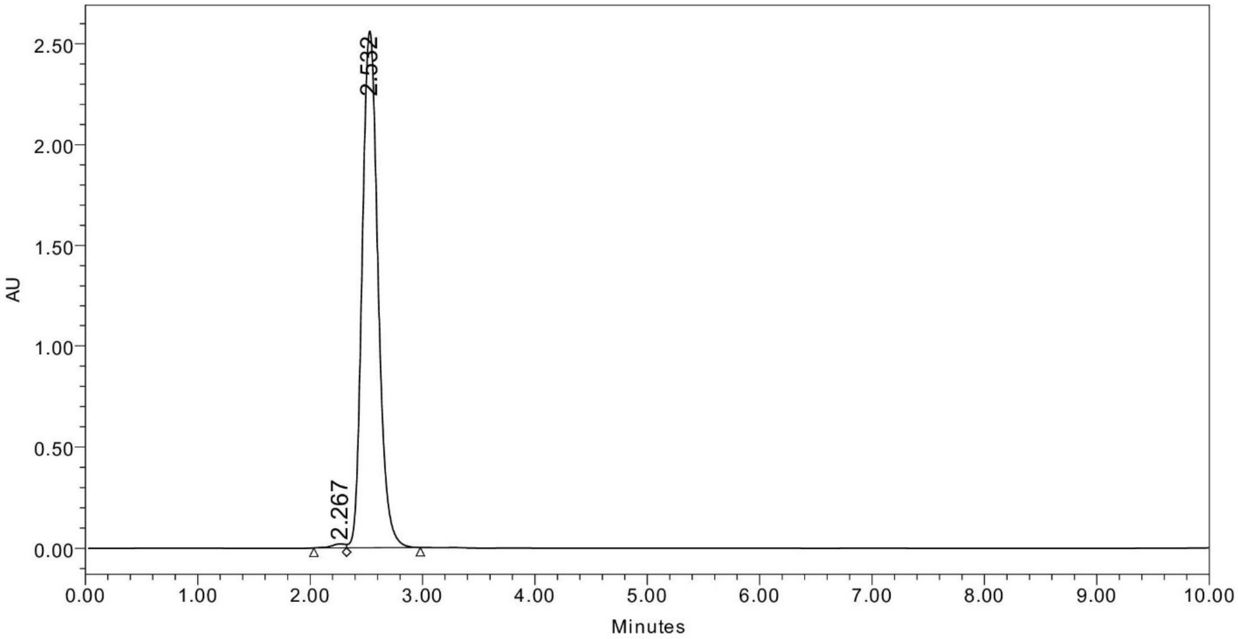

17-Cyclopropylmethyl-3,14*β*-dihydroxy-4,5*α*-epoxy-6*α*-[3'-(furan-2"-yl)propanamido]morphinan hydrochloride **(14)**

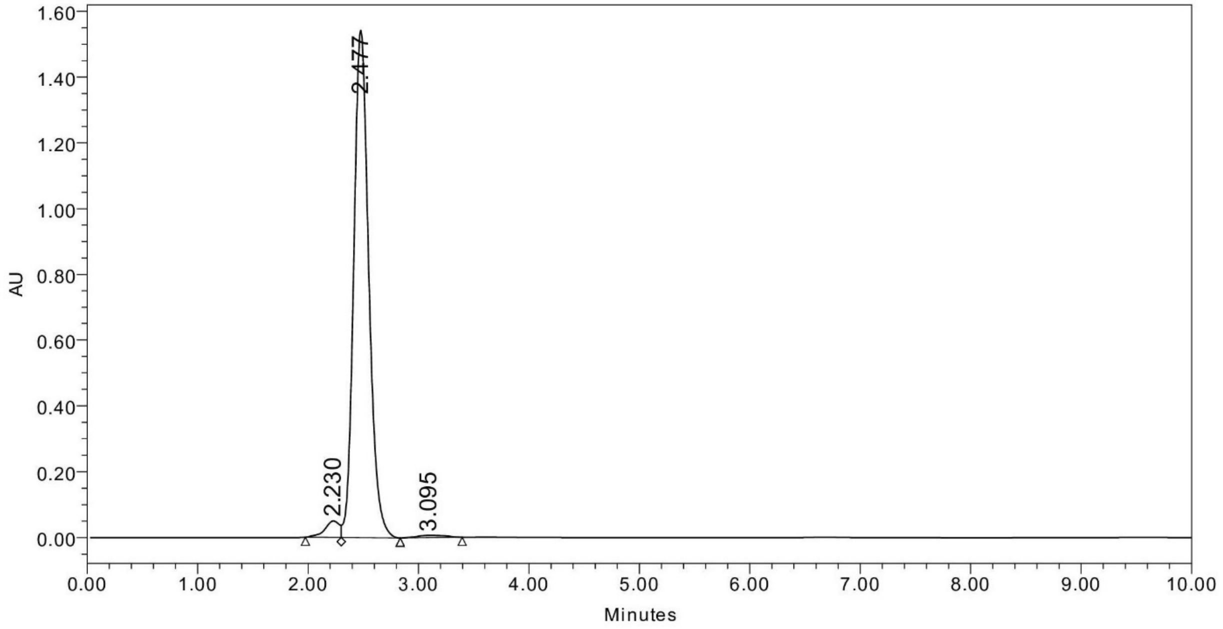

17-Cyclopropylmethyl-3,14*β*-dihydroxy-4,5*α*-epoxy-6*β*-[3'-(furan-2"-yl)-*N*-methylpropanamido]morphinan hydrochloride **(15)**

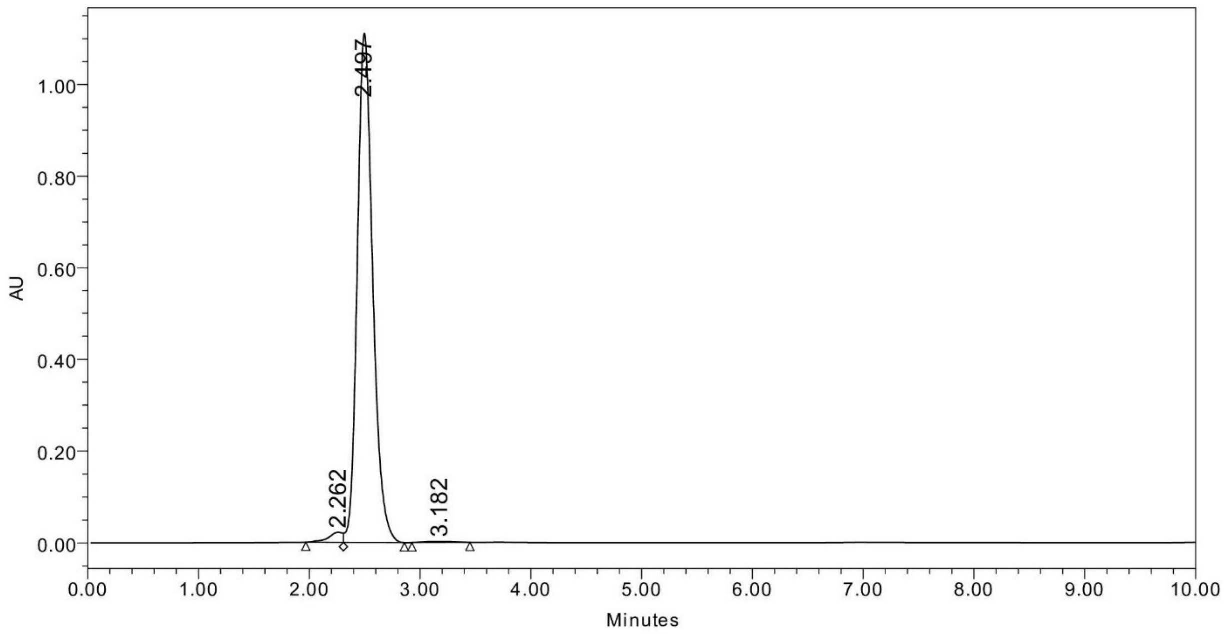

17-Cyclopropylmethyl-3,14*β*-dihydroxy-4,5*α*-epoxy-6*β*-[3'-(furan-2"-yl)propanamido]morphinan hydrochloride **(16)**

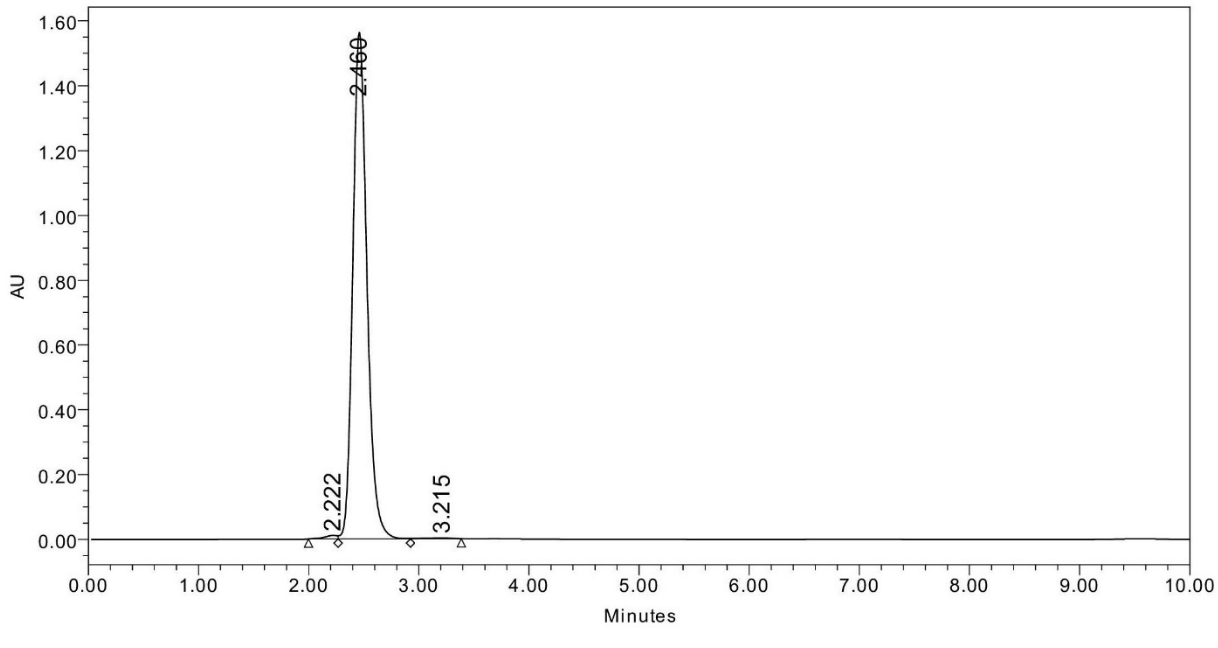

17-Cyclopropylmethyl-4,5*α*-epoxy-6*α*-[(2E)-3’-(furan-2”-yl)*N*-methylprop-2-enamido]-14*β*-hydroxymorphinan hydrochloride **(17)**

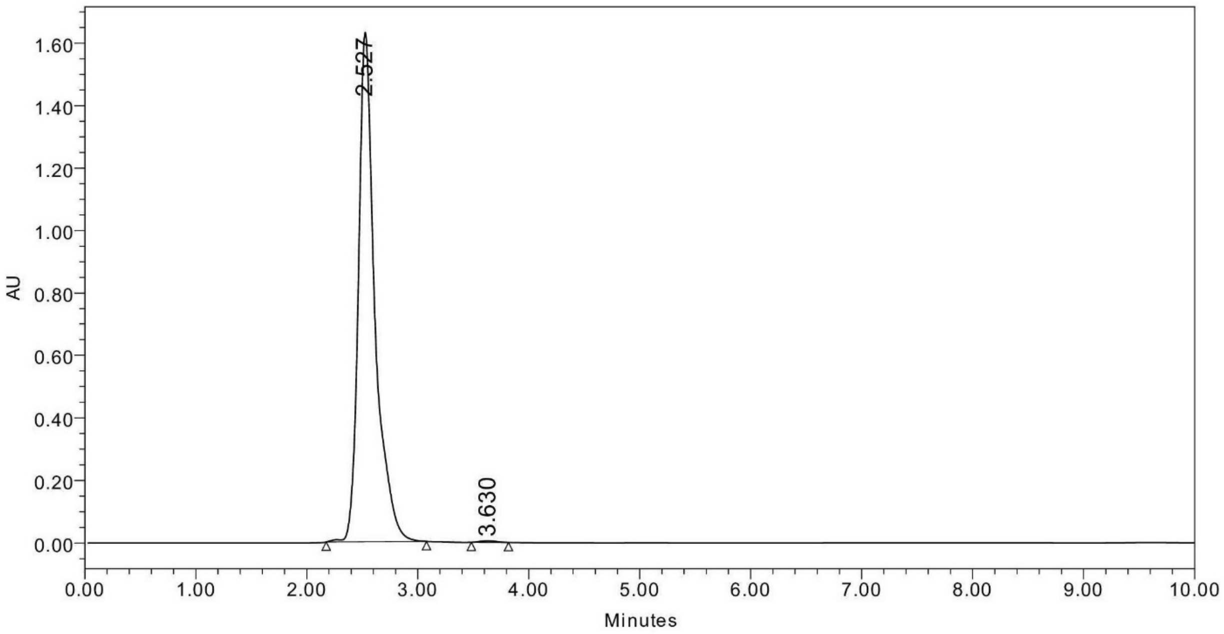

17-Cyclopropylmethyl-4,5*α*-epoxy-6*α*-[(2E)-3’-(furan-2”-yl)prop-2-enamido]-14*β*-hydroxymorphinan hydrochloride **(18)**

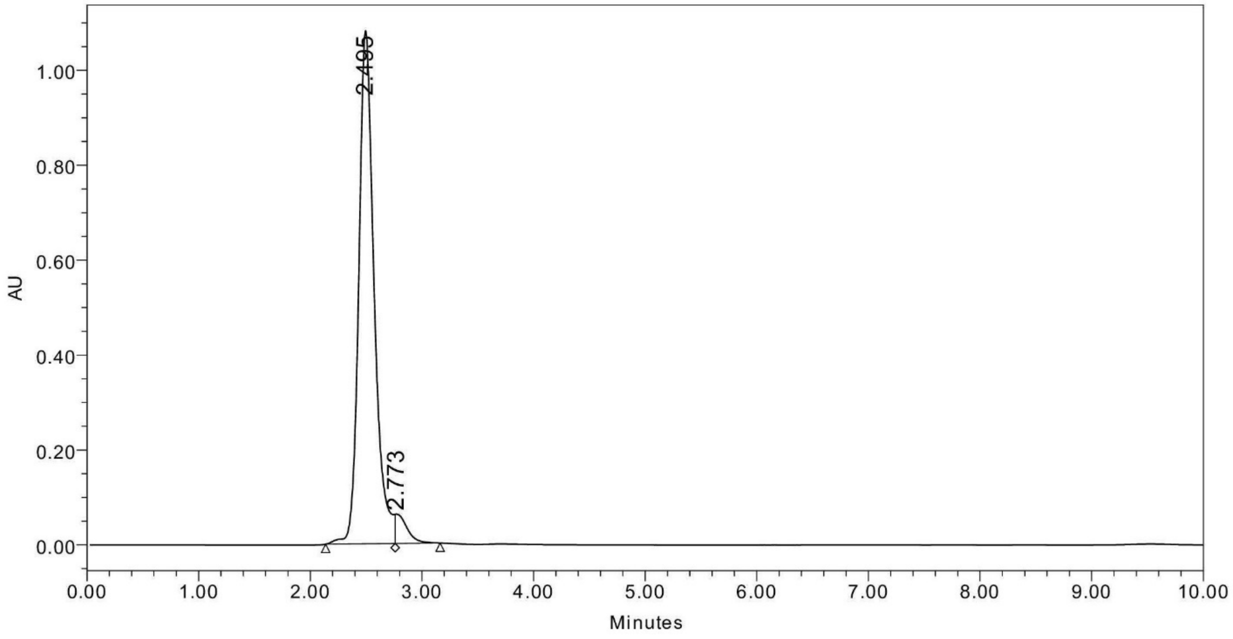

17-Cyclopropylmethyl-4,5*α*-epoxy-6*β*-[(2E)-3'-(furan-2''-yl)-*N*-methylprop-2-enamido]-14*β*-hydroxymorphinan hydrochloride **(19)**

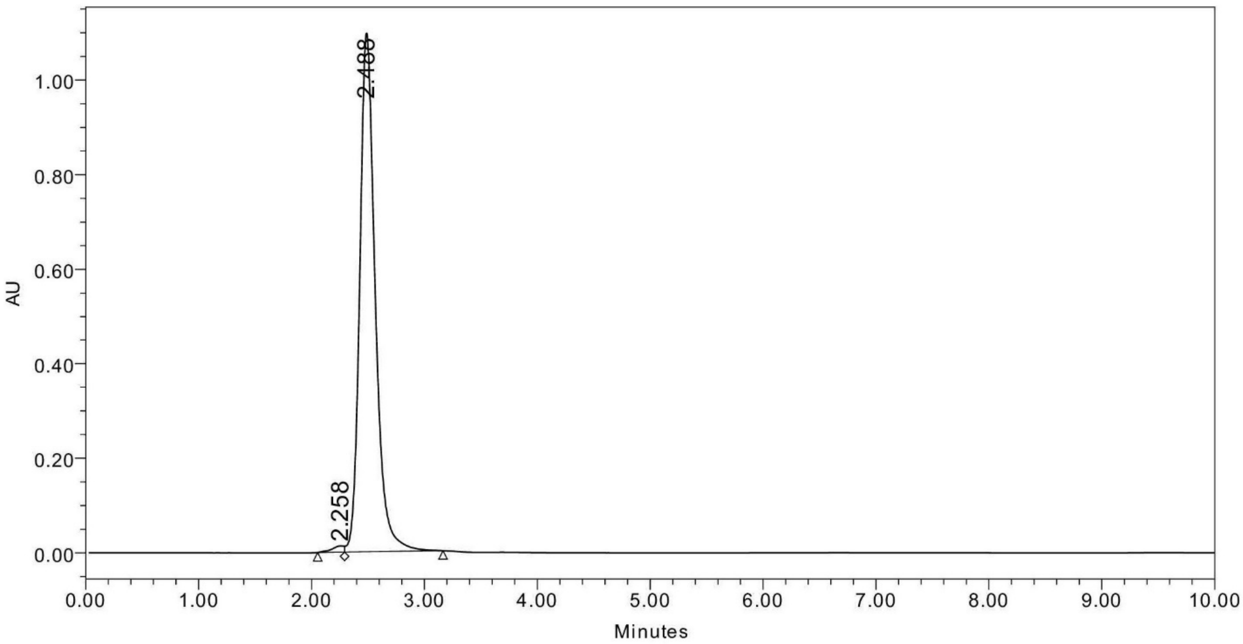

17-Cyclopropylmethyl-4,5*α*-epoxy-6*β*-[(2E)-3'-(furan-2''-yl)prop-2-enamido]-14*β*-hydroxymorphinan hydrochloride **(20)**

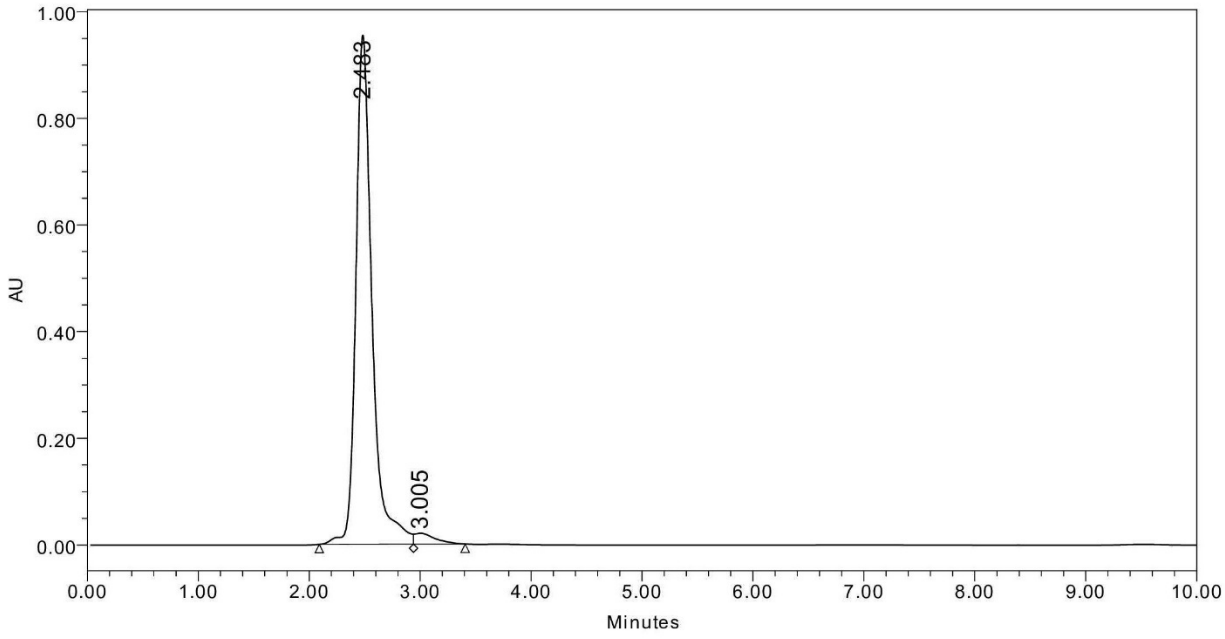

17-Cyclopropylmethyl-3,14β-dihydroxy-4,5α-epoxy-6α-[(2E)-3’-(furan-2”-yl)-N-methylprop-2-enamido]morphinan hydrochloride **(21)**

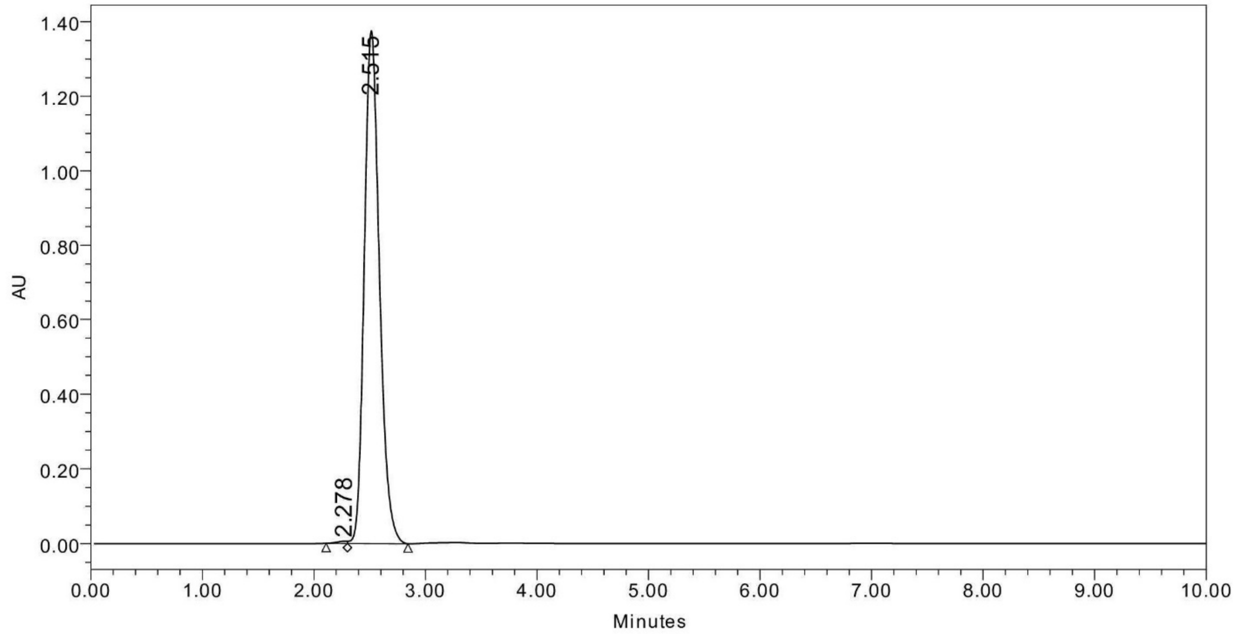

17-Cyclopropylmethyl-3,14β-dihydroxy-4,5α-epoxy-6α-[(2E)-3’-(furan-2”-yl)prop-2-enamido]morphinan hydrochloride **(22)**

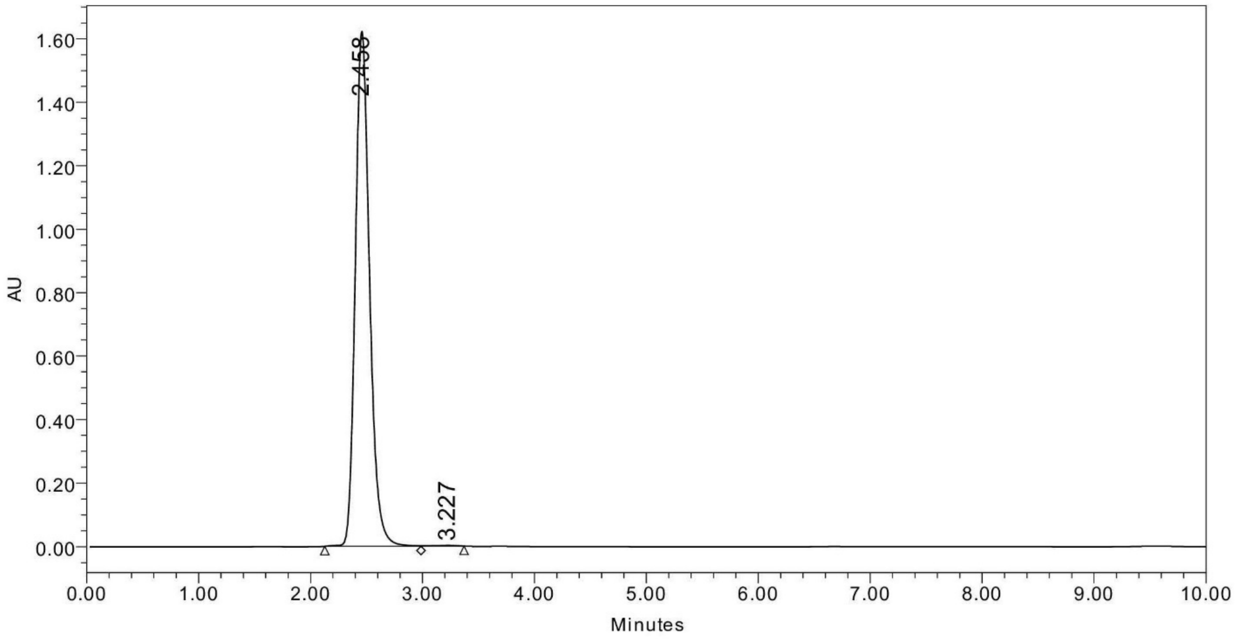

17-Cyclopropylmethyl-3,14β-dihydroxy-4,5α-epoxy-6β-[(2E)-3'-(furan-2"-yl)-*N*-methylprop-2-enamido]morphinan hydrochloride **(23)**

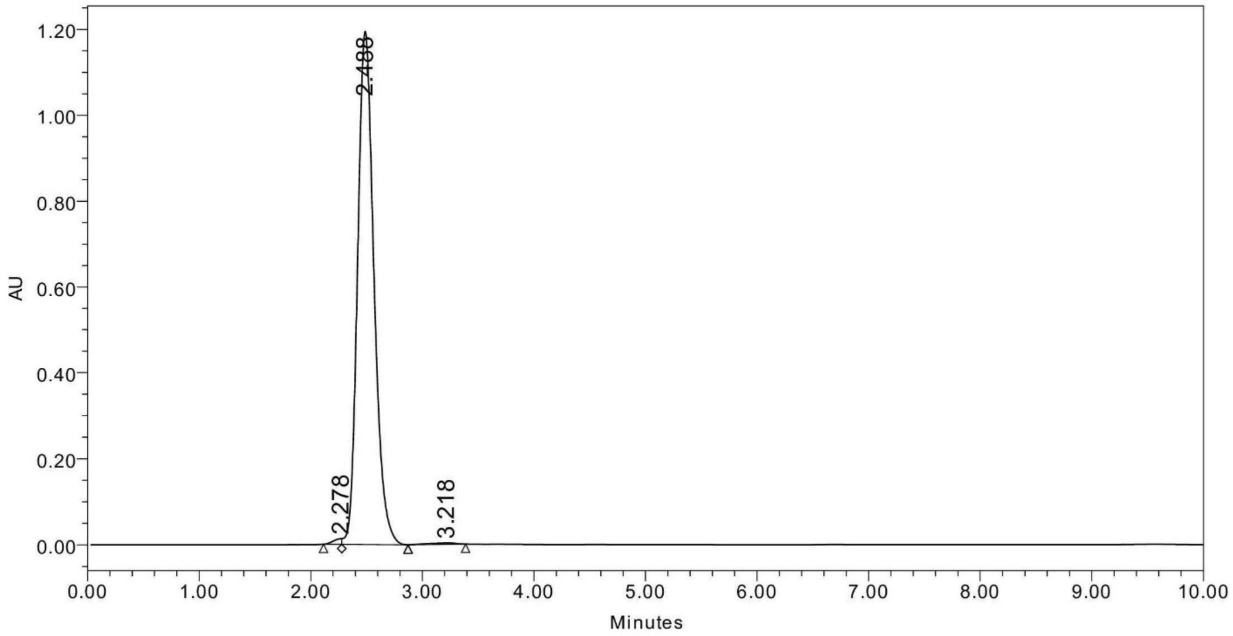

17-Cyclopropylmethyl-3,14β-dihydroxy-4,5α-epoxy-6β-[(2E)-3'-(furan-2"-yl)prop-2-enamido]morphinan hydrochloride **(24)**

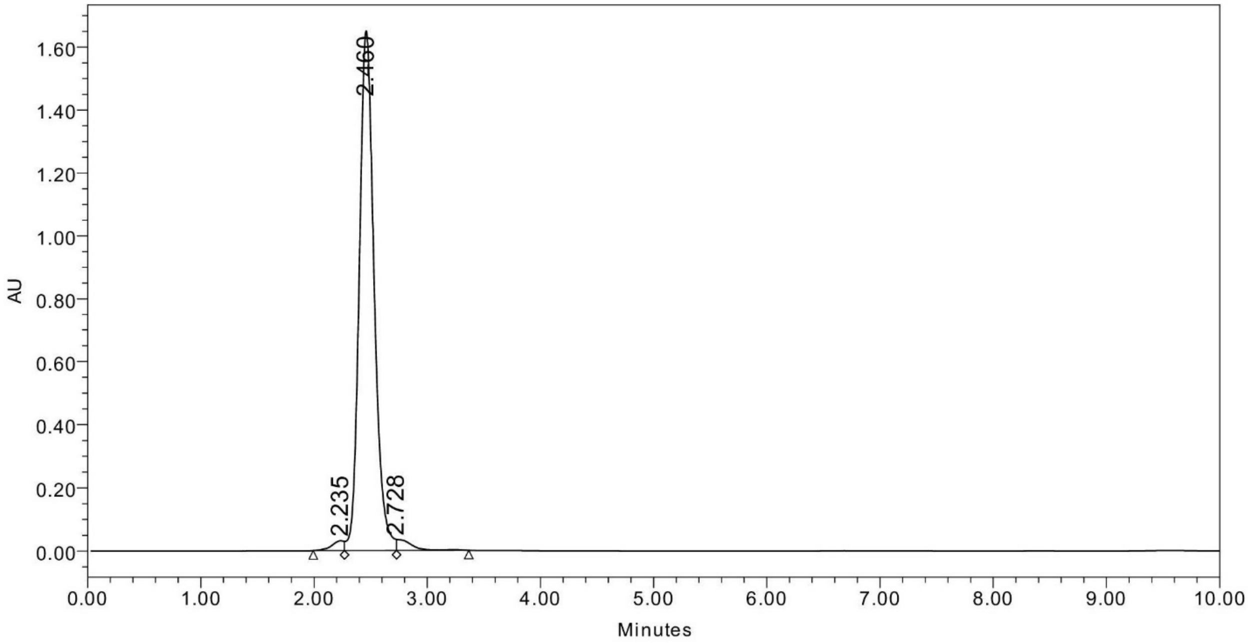

Supplement: Supplementary file 1 — jm4c00646_si_001.pdf [file jm4c00646_si_001.pdf]
